# Building a DNA barcode reference collection of Hymenoptera in New Zealand

**DOI:** 10.3897/BDJ.12.e131701

**Published:** 2024-09-26

**Authors:** Darren F Ward

**Affiliations:** 1 New Zealand Arthropod Collection (NZAC) - Landcare Research, Auckland, New Zealand New Zealand Arthropod Collection (NZAC) - Landcare Research Auckland New Zealand; 2 School of Biological Sciences, University of Auckland, Auckland, New Zealand School of Biological Sciences, University of Auckland Auckland New Zealand

**Keywords:** eDNA, molecular, monitoring, sequences, taxonomy

## Abstract

Molecular tools used for the identification of species are heavily reliant on reference DNA sequences and taxonomic annotation. Despite this, there are large gaps in the availability of DNA sequences for many taxonomic groups and for different parts of the globe. Here, a DNA barcode library for the Hymenoptera of New Zealand is presented, based on the COI region for 3,145 sequences assigned to 837 BINs and which represent 231 genera and 236 species. This study provides a DNA barcode for approximately 25% of species and 42% of genera of Hymenoptera in New Zealand. However, when combined with sequences previously deposited in BOLD (a further 170 genera), DNA barcodes are available for 73% of New Zealand Hymenopteran genera. To further increase coverage, future efforts need to focus predominantly on taxa from seven families (Encyrtidae, Pteromalidae s.l., Mymaridae, Eulophidae, Diapriidae, Braconidae and Platygastridae). This database facilitates DNA-based identification of taxa for use in both taxonomic revisions and biodiversity monitoring.

## Introduction

Molecular tools are now an indispensable part of biodiversity science and management for understanding biodiversity and ecology of species and communities, detecting threatened or invasive species, assessing environmental change and for taxonomy and systematics (Gwiazdowski et al. 2015, Hartop et al. 2021, Dopheide et al. 2022, Keck et al. 2022, Blackman et al. 2024, Lo et al. 2024, Macher et al. 2024). Indeed, large-scale biodiversity inventory projects are once again becoming popular because DNA barcoding can provide a fast and efficient tool for species identification (Butcher et al. 2012, Schmidt et al. 2017, Quicke et al. 2023). Additionally, environmental DNA (eDNA) has emerged relatively recently as a transformative tool for detecting and analysing the presence of species in the surrounding environment (Blackman et al. 2024, Leandro et al. 2024). However, the utility of all these molecular methods is heavily reliant on the availability of reference DNA sequences and their taxonomic annotation.

Molecular databases, such as GenBank [www.ncbi.nlm.nih.gov/genbank/] and the Barcode of Life (BOLD) database [www.boldsystems.org/] serve as repositories of reference DNA barcodes derived from specimens identified by taxonomists, against which DNA sequences can be compared and assigned to known taxa (Keck et al. 2022). Recently, the progress and needs for monitoring using eDNA in six key areas was reviewed (Blackman et al. 2024) and reference database development was strongly highlighted by the community survey as requiring the most attention. However, despite technological advancements and large increases in available sequences over the last decade, reference sequence databases still exhibit notable gaps in coverage, particularly for understudied taxonomic groups and different regions across the globe (Keck et al. 2022, Blackman et al. 2024, Leandro et al. 2024, Lo et al. 2024, Macher et al. 2024). Terrestrial arthropods are under-represented in sequence databases in comparison to other terrestrial animals (such as mammals and birds) (Leandro et al. 2024). For example, GenBank lacked sequences for ~ 83% of described invertebrate taxa, 83% for fungi, 62% for plants and 32% for vertebrates (Schoch et al. 2020). This limitation can restrict the usefulness of molecular approaches because it poses a significant challenge to the accurate identification of species and the subsequent interpretation of ecological patterns derived from sequence data (Blackman et al. 2024, Macher et al. 2024). For example, the lack of completeness in sequence databases can lead to the omission of whole phylogenetic clades and limits the use of these data to assess global biodiversity patterns of richness and turnover (Lo et al. 2024). The incompleteness of reference databases is also often used as an explanation for the non-detection of species that are present in a location (Keck et al. 2022).

Hymenoptera (ants, bees, wasps and sawflies) are one of the globally megadiverse orders of insects. They include some damaging pest species (e.g. social ants (Holway et al. 2002), social wasps (Beggs et al. 2011), plant damaging woodwasps and sawflies (Bain et al. 2011, Ward and Goulet 2021), but also many economically beneficial species, such as pollinators and species used for biocontrol (Horrocks et al. 2019, Ward et al. 2020). The identification of Hymenoptera is often very challenging, with relatively few regional identification guides, historically less taxonomic effort relative to other insect orders and, in many groups, the great majority of species are still undescribed (Forbes et al. 2018). Consequently, molecular approaches are ideal for studying Hymenoptera and, indeed, DNA sequences and barcoding have been used to transform our understanding of the higher level phylogeny of many groups and to discriminate between cryptic species as part of taxonomic revisionary work (Johnson et al. 2013, Praz et al. 2022, Santos and Brady 2023).

The current classification of Hymenoptera in New Zealand recognises 947 species in 546 genera from 52 families (Ward 2024). The non-native (exotic) fauna is well studied, with a large number of species that have been both accidentally and deliberately introduced (Ward and Edney-Browne 2015). However, a very large portion of the native fauna remains undescribed and has a high number of endemic species and genera and isolated lineages (e.g. Bouček and Noyes (1987), Fernandez-Triana et al. (2011), Quicke et al. (2019b), Quicke et al. (2019a)). This paper provides an overview of an authoritative DNA barcode sequence (COI) database for Hymenoptera in New Zealand and reports on taxon coverage and gaps. Such a database aims to enable DNA-based identification of taxa for use in both taxonomic revisions and biodiversity monitoring.

## Methods

**Sampling and Specimen Records**

Overall the DNA barcodes have accumulated since 2010 with specimens coming from three sources:

1. Field-based sampling from 2010 - 2023, using Malaise traps and sweep nets. This sampling has predominantly occurred within five regions: Auckland, Central Otago, Dunedin, Fiordland and the West Coast. This sampling was undertaken to obtain ‘fresh’ specimens specifically for DNA sequencing and this approach contributed 57% of all sequences.

2. Existing specimens in the New Zealand Arthropod Collection (NZAC) from either pinned or ethanol-based material (Ward and Malysheva 2022). The collection dates of specimens were typically from the 1980s-2000s, but the earliest record was 1929. This work was undertaken to increase the taxonomic and geographic coverage of sequences, and also to obtain sequences from named species (especially type specimens). This contributed 35% of sequences.

3. Specimens from queries sent to the NZAC for identification since 2010. This work was opportunistic, but helped to increase the taxonomic and geographic coverage of sequences and contributed 8% of sequences.

**Specimen Identification**

Specimens were morphologically examined and identified by comparing them to previously identified specimens in the NZAC, using taxonomic keys and expert knowledge. Sometimes specimens were identified before DNA processing and sequencing, which was typical of those specimens that came from the NZAC or from identification queries. However, the identification of many specimens was confirmed post-DNA extraction, based on building taxon trees in BOLD and then physical examination of the specimen (by the author). The BLAST function (Basic Local Alignment Search Tool) was not used as an identification tool because, whilst the DNA reference collection was being built, a BLAST provided either no taxonomic name, or an ambiguous or unlikely name. This is typical of databases where there is incomplete coverage, particularly of diverse and highly regionalised taxa (such as Hymenoptera in New Zealand). Taxon coverage and gaps were compared to an online checklist of taxa present in New Zealand (Ward 2024). The ‘taxonomy’ search function in BOLD was used to search for genera that were known to be present in New Zealand, but were not sequenced in this study and, if the genus were present in BOLD (with a barcode), then this increased the overall coverage *in addition* to the taxa identified in this study.

**DNA Processing and Data Accessibility**

From each specimen, one tissue sample (a leg, sometimes two legs depending on specimen size) was removed and stored in 95% ethanol for DNA extraction. Specimens were either processed at the Canadian Center for DNA Barcoding (www.ccdb.ca) or the Ecogene facility at Landcare Research, based on the COI-5P marker (https://www.landcareresearch.co.nz/partner-with-us/ecogene-dna-based-diagnostics/). Primers used were: LepF1/LepR1, MLepF1/LepR1 and LCO1490/HCO2198. All physical specimens are held in the NZAC and their details (e.g. collecting locality, dates etc.) are available through GBIF [www.gbif.org/] and specimen details, sequences and metadata are available in the laboratory information system in BOLD [www.boldsystems.org/] and more broadly (Ivanova et al. 2006).

## Results and Discussion

A total of 3145 sequences were obtained and assigned to 837 BINs, of which identifications were made for 236 named species and 231 genera (Suppl. materials 1, 2). Overall, this constitutes a DNA barcode library for 42% of Hymenoptera genera recognised as present in New Zealand (Table 1). Additionally, BOLD provides sequences for a further 166 genera, thus increasing the DNA coverage to 73% of all genera known to be present in New Zealand.

It is more challenging to obtain a “% coverage” at the species level. Approximately, 25% of named species are represented by a sequence (i.e. 236 named species in Supplementary Material 2 from an overall checklist of 947 species). Amongst the 871 BINS (Suppl. material 2), which are often used as a proxy for ‘species’ (Schmidt et al. 2015), there is evidence of both a large number of undescribed species and some named species that have not yet been matched to the sequence.

Coverage is higher for families with fewer genera and for groups that are well curated and revised (e.g. ‘Symphyta’ and Aculeata) or are part of current taxonomic projects (Braconidae, Ichneumonidae). However, a total of 148 genera still do not have a DNA barcode. The majority of these ‘gaps’ occur within seven families (Encyrtidae, Pteromalidae s.l., Mymaridae, Eulophidae, Diapriidae, Braconidae and Platygastridae).

It is well known that sequence databases exhibit notable taxonomic gaps in coverage (Keck et al. 2022, Blackman et al. 2024, Leandro et al. 2024). This study provides a major step forward in developing a comprehensive DNA reference collection for Hymenoptera in New Zealand. This is particularly important because New Zealand has a very high proportion of endemic taxa and are consequently less likely to be present in global databases without a focused and concerted effort. For example, there are 79 endemic genera of Hymenoptera in New Zealand and now 28 (35%) have a barcode; the remaining genera without a barcode fall mostly within the above families, have often been rarely collected and many should be considered "dark taxa” (sensu Page (2016)).

DNA barcode reference databases linked to voucher specimens create new opportunities for a range of future activities, including high-throughput identification and taxonomic revisions (Hartop et al. 2021), biogeographic and phylogenetic studies (Lo et al. 2024) and faster and cheaper biodiversity and biosecurity monitoring through eDNA (Leandro et al. 2024). Consequently, DNA barcode reference databases provide an important intergenerational resource, but one that should be still centred on authoritative identification and expertise and on open-data.

The development of DNA reference databases was recently highlighted by the community survey as requiring the most need (see fig. 2 in Blackman et al. (2024)). Ironically, the same survey also pointed out that publications on reference databases were consistently lower than for other key topic areas over time (see fig. 1 in Blackman et al. (2024)). It seems everyone wants a reference database, but few are willing to develop them.

Keck et al. (2022) considered that compiling reference databases was a time-consuming operation with many problems potentially affecting the results. Compiling the reference database in this paper for Hymenoptera was a time-consuming project involving multiple stages which were undertaken through an iterative process over ten years (e.g. field sampling, storage for preservation, accessing and curating existing specimens, identification, lab processing and sequencing and data curation). The ‘DNA lab stage’ was the easiest and quickest stage.

Several authors have suggested that addressing the challenge of ‘taxonomic gaps’ is urgently needed and requires a collaboration between ecologists, geneticists and taxonomic experts (Gwiazdowski et al. 2015, Keck et al. 2022, Blackman et al. 2024). Whilst true, supporting taxonomists (and taxonomic collections) is essential to provide the correct identification of voucher specimens (Ward et al. 2015, Santos and Brady 2023). Unfortunately, this very much remains a critically undervalued step.

## Supplementary Material

96ADBE64-E058-555A-BAD9-72C35ADD3798 10.3897/BDJ.12.e131701.suppl1 Supplementary material 1 Supplementary Material 1 Data type occurrences, lab Brief description Excel data file of information from BOLD-generated download of the metadata associated with DNA sequences, consisting of: lab information, sequence lengths, voucher codes, institution storing, taxonomy, specimen details and collection data (e.g. locality, dates etc.). File: oo_1130067.xlsx https://binary.pensoft.net/file/1130067 DF Ward

882DD20E-94F4-5D2E-A38B-A095AD88B46B 10.3897/BDJ.12.e131701.suppl2 Supplementary material 2 Supplementary Material 2 Data type taxonomic tree based on COI Brief description Nearest-neighbour taxonomic tree, based on COI sequences using the Kimura 2 Parameter Distance Model and aligned with the BOLD Aligner (amino acid based) with sequences only over 400 base pairs and without stop codons, contaminants or flagged as misidentifications or errors. File: oo_1082502.pdf https://binary.pensoft.net/file/1082502 DF Ward

## Figures and Tables

**Table 1.** Numbers of genera with (and without) a DNA barcode for each family. Table is organised alphabetically by family. Columns: #Genera in NZ (see https://en.wikipedia.org/wiki/Hymenoptera_in_New_Zealand); #Genera sequenced (see Supplementary material) from this study; Total #Genera available includes the combined information from this study and additional searches in BOLD.

Family	#Genera in NZ	#Genera sequenced (this study)	Total #Genera available (this study and BOLD)	%Coverage	#Genera to obtain
Agaonidae	1	1	1	100%	0
Aphelinidae	12	2	10	83%	2
Apidae	2	2	2	100%	0
Bembicidae	1	1	1	100%	0
Bethylidae	13	10	13	100%	0
Braconidae	78	55	66	85%	12
Ceraphronidae	2	1	2	100%	0
Chalcididae	3	0	3	100%	0
Colletidae	5	5	5	100%	0
Crabronidae	4	4	4	100%	0
Cynipidae	2	1	2	100%	0
Diapriidae	25	4	10	40%	15
Dryinidae	4	4	4	100%	0
Embolemidae	1	0	1	100%	0
Encyrtidae	39	4	14	36%	25
Eulophidae	50	10	34	68%	16
Eupelmidae	3	1	2	67%	1
Eurytomidae	5	2	3	60%	2
Figitidae	10	8	10	100%	0
Formicidae	27	24	27	100%	0
Gasteruptiidae	2	2	2	100%	0
Halictidae	2	1	2	100%	0
Ibaliidae	1	0	1	100%	0
Ichneumonidae	63	51	62	98%	1
Maamingidae	1	1	1	100%	0
Megachilidae	3	1	3	100%	0
Megaspilidae	4	2	3	75%	1
Megastigmidae	1	0	1	100%	0
Mutillidae	1	1	1	100%	0
Mymaridae	40	2	20	50%	20
Mymarommatidae	2	0	0	0%	2
Orussidae	1	0	1	100%	0
Pemphredonidae	1	1	1	100%	0
Pergidae	1	1	1	100%	0
Perilampidae	1	0	1	100%	0
Platygastridae	21	1	12	57%	9
Pompilidae	4	4	4	100%	0
Proctotrupidae	3	2	3	100%	0
Pteromalidae	53	7	30	57%	23
Rotoitidae	1	0	0	0%	1
Scelionidae	22	7	15	74%	7
Scolebythidae	1	0	0	0%	1
Scoliidae	1	1	1	100%	0
Signiphoridae	2	0	2	100%	0
Siricidae	1	0	1	100%	0
Sparasionidae	1	1	1	100%	0
Sphecidae	1	0	1	100%	0
Tenthredinidae	4	3	4	100%	0
Torymidae	5	0	3	60%	2
Trichogrammatidae	11	0	3	27%	8
Vespidae	3	3	3	100%	0
Xiphydriidae	1	1	1	100%	0
