# Supplementary material for: Building a DNA barcode reference collection of Hymenoptera in New Zealand
Source: Biodivers Data J. 2024 Sep 26;12:e131701. doi: 10.3897/BDJ.12.e131701 (PMC11450381; doi:10.3897/BDJ.12.e131701)
Supplement: Supplementary material 2 [file bdj-12-e131701-s002.pdf]

BOLD TaxonID Tree

Title : Tree Result - Search: Sample IDs; Include public records (3145 records returned) (3145 records selected)
Date : 14-May-2024
Data Type : Nucleotide
Distance Model : Kimura 2 Parameter
Marker : COI-5P
Colourization : Barcode Cluster (BIN)

Label : Sample ID
Label : Process ID
Label : Taxon
Label : Barcode Cluster (BIN)

Filter : length > 400bp only
Filter : exclude records flagged as misidentifications
Filter : exclude records with stop codons
Filter : exclude contaminants

Sequence Count : 3145
Species count : 236
Genus count : 231
Family count : 42
Unidentified : 2309

BIN Count : 837

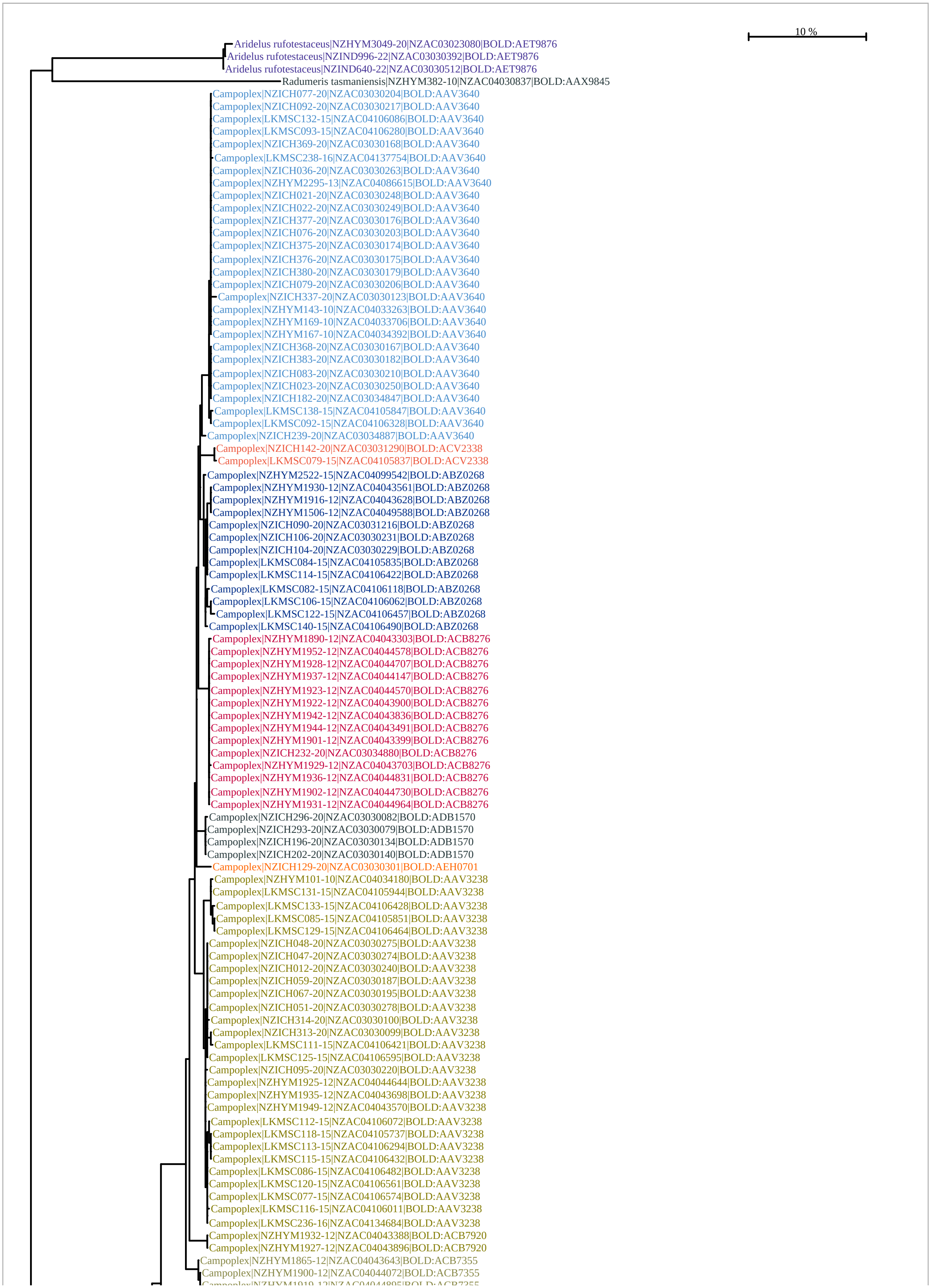

10 %
Aridelus rufotestaceus|NZHYM3049-20|NZAC03023080|BOLD:AET9876
Aridelus rufotestaceus|NZIND996-22|NZAC03030392|BOLD:AET9876
Aridelus rufotestaceus|NZIND640-22|NZAC03030512|BOLD:AET9876
Radumeris tasmaniensis|NZHYM382-10|NZAC04030837|BOLD:AAX9845
Campoplex|NZICH077-20|NZAC03030204|BOLD:AAV3640
Campoplex|NZICH092-20|NZAC03030217|BOLD:AAV3640
Campoplex|LKMSC132-15|NZAC04106086|BOLD:AAV3640
Campoplex|LKMSC093-15|NZAC04106280|BOLD:AAV3640
Campoplex|NZICH369-20|NZAC03030168|BOLD:AAV3640
Campoplex|LKMSC238-16|NZAC04137754|BOLD:AAV3640
Campoplex|NZICH036-20|NZAC03030263|BOLD:AAV3640
Campoplex|NZHYM2295-13|NZAC04086615|BOLD:AAV3640
Campoplex|NZICH021-20|NZAC03030248|BOLD:AAV3640
Campoplex|NZICH022-20|NZAC03030249|BOLD:AAV3640
Campoplex|NZICH377-20|NZAC03030176|BOLD:AAV3640
Campoplex|NZICH076-20|NZAC03030203|BOLD:AAV3640
Campoplex|NZICH375-20|NZAC03030174|BOLD:AAV3640
Campoplex|NZICH376-20|NZAC03030175|BOLD:AAV3640
Campoplex|NZICH380-20|NZAC03030179|BOLD:AAV3640
Campoplex|NZICH079-20|NZAC03030206|BOLD:AAV3640
Campoplex|NZICH337-20|NZAC03030123|BOLD:AAV3640
Campoplex|NZHYM143-10|NZAC04033263|BOLD:AAV3640
Campoplex|NZHYM169-10|NZAC04033706|BOLD:AAV3640
Campoplex|NZHYM167-10|NZAC04034392|BOLD:AAV3640
Campoplex|NZICH368-20|NZAC03030167|BOLD:AAV3640
Campoplex|NZICH383-20|NZAC03030182|BOLD:AAV3640
Campoplex|NZICH083-20|NZAC03030210|BOLD:AAV3640
Campoplex|NZICH023-20|NZAC03030250|BOLD:AAV3640
Campoplex|NZICH182-20|NZAC03034847|BOLD:AAV3640
Campoplex|LKMSC138-15|NZAC04105847|BOLD:AAV3640
Campoplex|LKMSC092-15|NZAC04106328|BOLD:AAV3640
Campoplex|NZICH239-20|NZAC03034887|BOLD:AAV3640
Campoplex|NZICH142-20|NZAC03031290|BOLD:ACV2338
Campoplex|LKMSC079-15|NZAC04105837|BOLD:ACV2338
Campoplex|NZHYM2522-15|NZAC04099542|BOLD:ABZ0268
Campoplex|NZHYM1930-12|NZAC04043561|BOLD:ABZ0268
Campoplex|NZHYM1916-12|NZAC04043628|BOLD:ABZ0268
Campoplex|NZHYM1506-12|NZAC04049588|BOLD:ABZ0268
Campoplex|NZICH090-20|NZAC03031216|BOLD:ABZ0268
Campoplex|NZICH106-20|NZAC03030231|BOLD:ABZ0268
Campoplex|NZICH104-20|NZAC03030229|BOLD:ABZ0268
Campoplex|LKMSC084-15|NZAC04105835|BOLD:ABZ0268
Campoplex|LKMSC114-15|NZAC04106422|BOLD:ABZ0268
Campoplex|LKMSC082-15|NZAC04106118|BOLD:ABZ0268
Campoplex|LKMSC106-15|NZAC04106062|BOLD:ABZ0268
Campoplex|LKMSC122-15|NZAC04106457|BOLD:ABZ0268
Campoplex|LKMSC140-15|NZAC04106490|BOLD:ABZ0268
Campoplex|NZHYM1890-12|NZAC04043303|BOLD:ACB8276
Campoplex|NZHYM1952-12|NZAC04044578|BOLD:ACB8276
Campoplex|NZHYM1928-12|NZAC04044707|BOLD:ACB8276
Campoplex|NZHYM1937-12|NZAC04044147|BOLD:ACB8276
Campoplex|NZHYM1923-12|NZAC04044570|BOLD:ACB8276
Campoplex|NZHYM1922-12|NZAC04043900|BOLD:ACB8276
Campoplex|NZHYM1942-12|NZAC04043836|BOLD:ACB8276
Campoplex|NZHYM1944-12|NZAC04043491|BOLD:ACB8276
Campoplex|NZHYM1901-12|NZAC04043399|BOLD:ACB8276
Campoplex|NZICH232-20|NZAC03034880|BOLD:ACB8276
Campoplex|NZHYM1929-12|NZAC04043703|BOLD:ACB8276
Campoplex|NZHYM1936-12|NZAC04044831|BOLD:ACB8276
Campoplex|NZHYM1902-12|NZAC04044730|BOLD:ACB8276
Campoplex|NZHYM1931-12|NZAC04044964|BOLD:ACB8276
Campoplex|NZICH296-20|NZAC03030082|BOLD:ADB1570
Campoplex|NZICH293-20|NZAC03030079|BOLD:ADB1570
Campoplex|NZICH196-20|NZAC03030134|BOLD:ADB1570
Campoplex|NZICH202-20|NZAC03030140|BOLD:ADB1570
Campoplex|NZICH129-20|NZAC03030301|BOLD:AEH0701
Campoplex|NZHYM101-10|NZAC04034180|BOLD:AAV3238
Campoplex|LKMSC131-15|NZAC04105944|BOLD:AAV3238
Campoplex|LKMSC133-15|NZAC04106428|BOLD:AAV3238
Campoplex|LKMSC085-15|NZAC04105851|BOLD:AAV3238
Campoplex|LKMSC129-15|NZAC04106464|BOLD:AAV3238
Campoplex|NZICH048-20|NZAC03030275|BOLD:AAV3238
Campoplex|NZICH047-20|NZAC03030274|BOLD:AAV3238
Campoplex|NZICH012-20|NZAC03030240|BOLD:AAV3238
Campoplex|NZICH059-20|NZAC03030187|BOLD:AAV3238
Campoplex|NZICH067-20|NZAC03030195|BOLD:AAV3238
Campoplex|NZICH051-20|NZAC03030278|BOLD:AAV3238
Campoplex|NZICH314-20|NZAC03030100|BOLD:AAV3238
Campoplex|NZICH313-20|NZAC03030099|BOLD:AAV3238
Campoplex|LKMSC111-15|NZAC04106421|BOLD:AAV3238
Campoplex|LKMSC125-15|NZAC04106595|BOLD:AAV3238
Campoplex|NZICH095-20|NZAC03030220|BOLD:AAV3238
Campoplex|NZHYM1925-12|NZAC04044644|BOLD:AAV3238
Campoplex|NZHYM1935-12|NZAC04043698|BOLD:AAV3238
Campoplex|NZHYM1949-12|NZAC04043570|BOLD:AAV3238
Campoplex|LKMSC112-15|NZAC04106072|BOLD:AAV3238
Campoplex|LKMSC118-15|NZAC04105737|BOLD:AAV3238
Campoplex|LKMSC113-15|NZAC04106294|BOLD:AAV3238
Campoplex|LKMSC115-15|NZAC04106432|BOLD:AAV3238
Campoplex|LKMSC086-15|NZAC04106482|BOLD:AAV3238
Campoplex|LKMSC120-15|NZAC04106561|BOLD:AAV3238
Campoplex|LKMSC077-15|NZAC04106574|BOLD:AAV3238
Campoplex|LKMSC116-15|NZAC04106011|BOLD:AAV3238
Campoplex|LKMSC236-16|NZAC04134684|BOLD:AAV3238
Campoplex|NZHYM1932-12|NZAC04043388|BOLD:ACB7920
Campoplex|NZHYM1927-12|NZAC04043896|BOLD:ACB7920
Campoplex|NZHYM1865-12|NZAC04043643|BOLD:ACB7355
Campoplex|NZHYM1900-12|NZAC04044072|BOLD:ACB7355

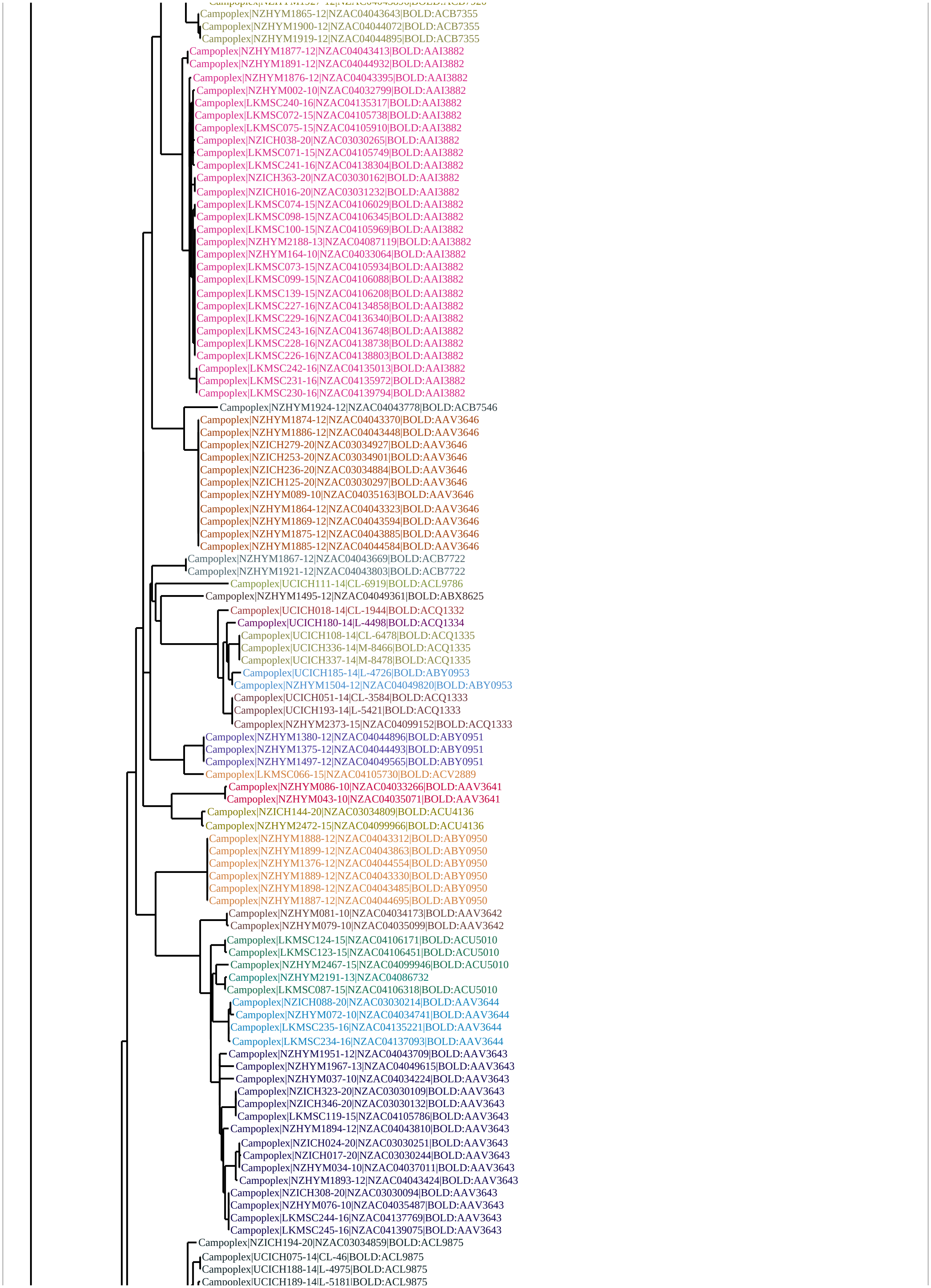
Campoplex|NZHYM1865-12|NZAC04043643|BOLD:ACB7355
Campoplex|NZHYM1900-12|NZAC04044072|BOLD:ACB7355
Campoplex|NZHYM1919-12|NZAC04044895|BOLD:ACB7355
Campoplex|NZHYM1877-12|NZAC04043413|BOLD:AAI3882
Campoplex|NZHYM1891-12|NZAC04044932|BOLD:AAI3882
Campoplex|NZHYM1876-12|NZAC04043395|BOLD:AAI3882
Campoplex|NZHYM002-10|NZAC04032799|BOLD:AAI3882
Campoplex|LKMSC240-16|NZAC04135317|BOLD:AAI3882
Campoplex|LKMSC072-15|NZAC04105738|BOLD:AAI3882
Campoplex|LKMSC075-15|NZAC04105910|BOLD:AAI3882
Campoplex|NZICH038-20|NZAC03030265|BOLD:AAI3882
Campoplex|LKMSC071-15|NZAC04105749|BOLD:AAI3882
Campoplex|LKMSC241-16|NZAC04138304|BOLD:AAI3882
Campoplex|NZICH363-20|NZAC03030162|BOLD:AAI3882
Campoplex|NZICH016-20|NZAC03031232|BOLD:AAI3882
Campoplex|LKMSC074-15|NZAC04106029|BOLD:AAI3882
Campoplex|LKMSC098-15|NZAC04106345|BOLD:AAI3882
Campoplex|LKMSC100-15|NZAC04105969|BOLD:AAI3882
Campoplex|NZHYM2188-13|NZAC04087119|BOLD:AAI3882
Campoplex|NZHYM164-10|NZAC04033064|BOLD:AAI3882
Campoplex|LKMSC073-15|NZAC04105934|BOLD:AAI3882
Campoplex|LKMSC099-15|NZAC04106088|BOLD:AAI3882
Campoplex|LKMSC139-15|NZAC04106208|BOLD:AAI3882
Campoplex|LKMSC227-16|NZAC04134858|BOLD:AAI3882
Campoplex|LKMSC229-16|NZAC04136340|BOLD:AAI3882
Campoplex|LKMSC243-16|NZAC04136748|BOLD:AAI3882
Campoplex|LKMSC228-16|NZAC04138738|BOLD:AAI3882
Campoplex|LKMSC226-16|NZAC04138803|BOLD:AAI3882
Campoplex|LKMSC242-16|NZAC04135013|BOLD:AAI3882
Campoplex|LKMSC231-16|NZAC04135972|BOLD:AAI3882
Campoplex|LKMSC230-16|NZAC04139794|BOLD:AAI3882
Campoplex|NZHYM1924-12|NZAC04043778|BOLD:ACB7546
Campoplex|NZHYM1874-12|NZAC04043370|BOLD:AAV3646
Campoplex|NZHYM1886-12|NZAC04043448|BOLD:AAV3646
Campoplex|NZICH279-20|NZAC03034927|BOLD:AAV3646
Campoplex|NZICH253-20|NZAC03034901|BOLD:AAV3646
Campoplex|NZICH236-20|NZAC03034884|BOLD:AAV3646
Campoplex|NZICH125-20|NZAC03030297|BOLD:AAV3646
Campoplex|NZHYM089-10|NZAC04035163|BOLD:AAV3646
Campoplex|NZHYM1864-12|NZAC04043323|BOLD:AAV3646
Campoplex|NZHYM1869-12|NZAC04043594|BOLD:AAV3646
Campoplex|NZHYM1875-12|NZAC04043885|BOLD:AAV3646
Campoplex|NZHYM1885-12|NZAC04044584|BOLD:AAV3646
Campoplex|NZHYM1867-12|NZAC04043669|BOLD:ACB7722
Campoplex|NZHYM1921-12|NZAC04043803|BOLD:ACB7722
Campoplex|UCICH111-14|CL-6919|BOLD:ACL9786
Campoplex|NZHYM1495-12|NZAC04049361|BOLD:ABX8625
Campoplex|UCICH018-14|CL-1944|BOLD:ACQ1332
Campoplex|UCICH180-14|L-4498|BOLD:ACQ1334
Campoplex|UCICH108-14|CL-6478|BOLD:ACQ1335
Campoplex|UCICH336-14|M-8466|BOLD:ACQ1335
Campoplex|UCICH337-14|M-8478|BOLD:ACQ1335
Campoplex|UCICH185-14|L-4726|BOLD:ABY0953
Campoplex|NZHYM1504-12|NZAC04049820|BOLD:ABY0953
Campoplex|UCICH051-14|CL-3584|BOLD:ACQ1333
Campoplex|UCICH193-14|L-5421|BOLD:ACQ1333
Campoplex|NZHYM2373-15|NZAC04099152|BOLD:ACQ1333
Campoplex|NZHYM1380-12|NZAC04044896|BOLD:ABY0951
Campoplex|NZHYM1375-12|NZAC04044493|BOLD:ABY0951
Campoplex|NZHYM1497-12|NZAC04049565|BOLD:ABY0951
Campoplex|LKMSC066-15|NZAC04105730|BOLD:ACV2889
Campoplex|NZHYM086-10|NZAC04033266|BOLD:AAV3641
Campoplex|NZHYM043-10|NZAC04035071|BOLD:AAV3641
Campoplex|NZICH144-20|NZAC03034809|BOLD:ACU4136
Campoplex|NZHYM2472-15|NZAC04099966|BOLD:ACU4136
Campoplex|NZHYM1888-12|NZAC04043312|BOLD:ABY0950
Campoplex|NZHYM1899-12|NZAC04043863|BOLD:ABY0950
Campoplex|NZHYM1376-12|NZAC04044554|BOLD:ABY0950
Campoplex|NZHYM1889-12|NZAC04043330|BOLD:ABY0950
Campoplex|NZHYM1898-12|NZAC04043485|BOLD:ABY0950
Campoplex|NZHYM1887-12|NZAC04044695|BOLD:ABY0950
Campoplex|NZHYM081-10|NZAC04034173|BOLD:AAV3642
Campoplex|NZHYM079-10|NZAC04035099|BOLD:AAV3642
Campoplex|LKMSC124-15|NZAC04106171|BOLD:ACU5010
Campoplex|LKMSC123-15|NZAC04106451|BOLD:ACU5010
Campoplex|NZHYM2467-15|NZAC04099946|BOLD:ACU5010
Campoplex|NZHYM2191-13|NZAC04086732
Campoplex|LKMSC087-15|NZAC04106318|BOLD:ACU5010
Campoplex|NZICH088-20|NZAC03030214|BOLD:AAV3644
Campoplex|NZHYM072-10|NZAC04034741|BOLD:AAV3644
Campoplex|LKMSC235-16|NZAC04135221|BOLD:AAV3644
Campoplex|LKMSC234-16|NZAC04137093|BOLD:AAV3644
Campoplex|NZHYM1951-12|NZAC04043709|BOLD:AAV3643
Campoplex|NZHYM1967-13|NZAC04049615|BOLD:AAV3643
Campoplex|NZHYM037-10|NZAC04034224|BOLD:AAV3643
Campoplex|NZICH323-20|NZAC03030109|BOLD:AAV3643
Campoplex|NZICH346-20|NZAC03030132|BOLD:AAV3643
Campoplex|LKMSC119-15|NZAC04105786|BOLD:AAV3643
Campoplex|NZHYM1894-12|NZAC04043810|BOLD:AAV3643
Campoplex|NZICH024-20|NZAC03030251|BOLD:AAV3643
Campoplex|NZICH017-20|NZAC03030244|BOLD:AAV3643
Campoplex|NZHYM034-10|NZAC04037011|BOLD:AAV3643
Campoplex|NZHYM1893-12|NZAC04043424|BOLD:AAV3643
Campoplex|NZICH308-20|NZAC03030094|BOLD:AAV3643
Campoplex|NZHYM076-10|NZAC04035487|BOLD:AAV3643
Campoplex|LKMSC244-16|NZAC04137769|BOLD:AAV3643
Campoplex|LKMSC245-16|NZAC04139075|BOLD:AAV3643
Campoplex|NZICH194-20|NZAC03034859|BOLD:ACL9875
Campoplex|UCICH075-14|CL-46|BOLD:ACL9875
Campoplex|UCICH188-14|L-4975|BOLD:ACL9875
Campoplex|UCICH189-14|L-5181|BOLD:ACL9875

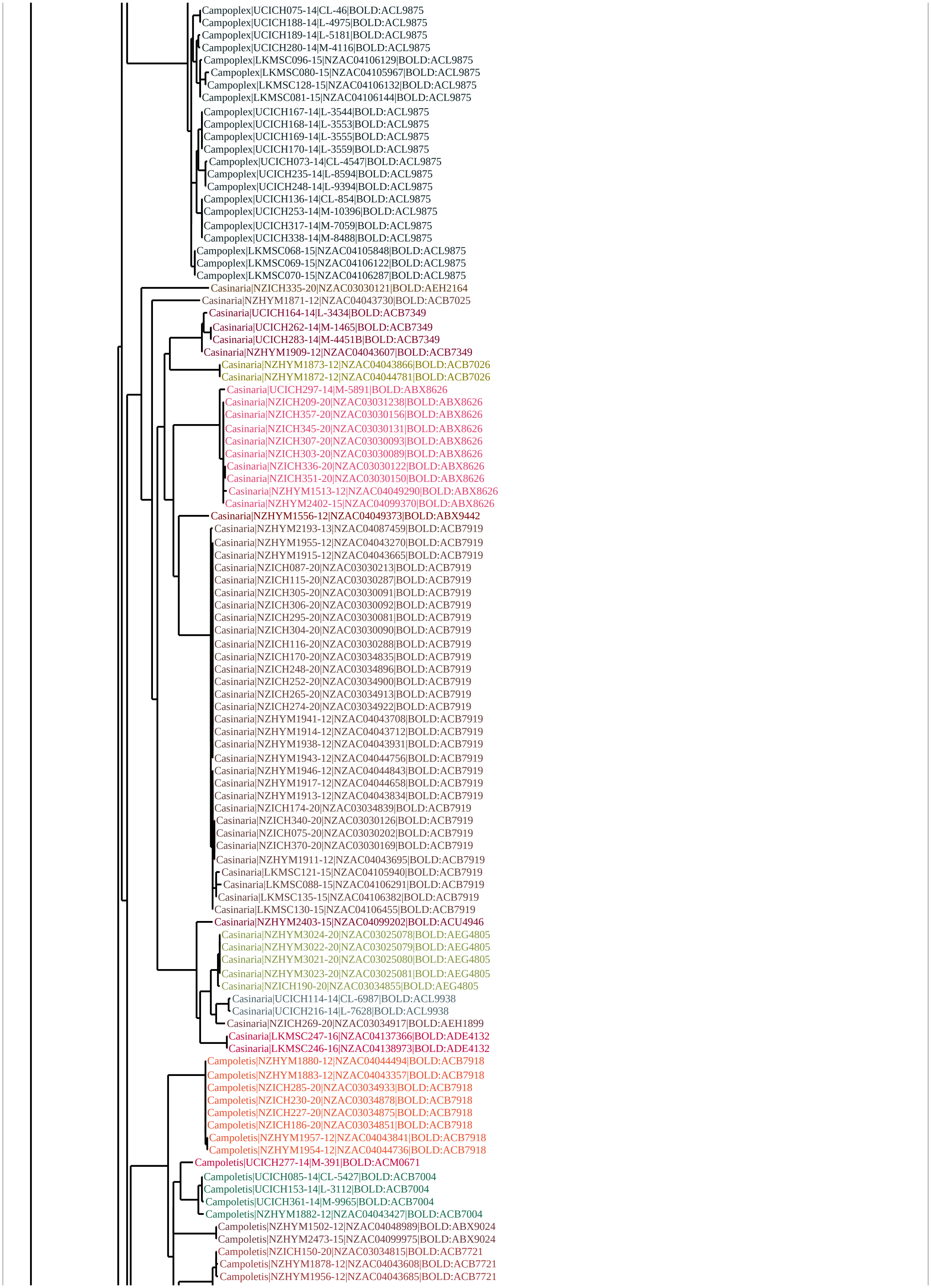
Campoplex|UCICH075-14|CL-46|BOLD:ACL9875
Campoplex|UCICH188-14|L-4975|BOLD:ACL9875
Campoplex|UCICH189-14|L-5181|BOLD:ACL9875
Campoplex|UCICH280-14|M-4116|BOLD:ACL9875
Campoplex|LKMSC096-15|NZAC04106129|BOLD:ACL9875
Campoplex|LKMSC080-15|NZAC04105967|BOLD:ACL9875
Campoplex|LKMSC128-15|NZAC04106132|BOLD:ACL9875
Campoplex|LKMSC081-15|NZAC04106144|BOLD:ACL9875
Campoplex|UCICH167-14|L-3544|BOLD:ACL9875
Campoplex|UCICH168-14|L-3553|BOLD:ACL9875
Campoplex|UCICH169-14|L-3555|BOLD:ACL9875
Campoplex|UCICH170-14|L-3559|BOLD:ACL9875
Campoplex|UCICH073-14|CL-4547|BOLD:ACL9875
Campoplex|UCICH235-14|L-8594|BOLD:ACL9875
Campoplex|UCICH248-14|L-9394|BOLD:ACL9875
Campoplex|UCICH136-14|CL-854|BOLD:ACL9875
Campoplex|UCICH253-14|M-10396|BOLD:ACL9875
Campoplex|UCICH317-14|M-7059|BOLD:ACL9875
Campoplex|UCICH338-14|M-8488|BOLD:ACL9875
Campoplex|LKMSC068-15|NZAC04105848|BOLD:ACL9875
Campoplex|LKMSC069-15|NZAC04106122|BOLD:ACL9875
Campoplex|LKMSC070-15|NZAC04106287|BOLD:ACL9875
Casinaria|NZICH335-20|NZAC03030121|BOLD:AEH2164
Casinaria|NZHYM1871-12|NZAC04043730|BOLD:ACB7025
Casinaria|UCICH164-14|L-3434|BOLD:ACB7349
Casinaria|UCICH262-14|M-1465|BOLD:ACB7349
Casinaria|UCICH283-14|M-4451B|BOLD:ACB7349
Casinaria|NZHYM1909-12|NZAC04043607|BOLD:ACB7349
Casinaria|NZHYM1873-12|NZAC04043866|BOLD:ACB7026
Casinaria|NZHYM1872-12|NZAC04044781|BOLD:ACB7026
Casinaria|UCICH297-14|M-5891|BOLD:ABX8626
Casinaria|NZICH209-20|NZAC03031238|BOLD:ABX8626
Casinaria|NZICH357-20|NZAC03030156|BOLD:ABX8626
Casinaria|NZICH345-20|NZAC03030131|BOLD:ABX8626
Casinaria|NZICH307-20|NZAC03030093|BOLD:ABX8626
Casinaria|NZICH303-20|NZAC03030089|BOLD:ABX8626
Casinaria|NZICH336-20|NZAC03030122|BOLD:ABX8626
Casinaria|NZICH351-20|NZAC03030150|BOLD:ABX8626
Casinaria|NZHYM1513-12|NZAC04049290|BOLD:ABX8626
Casinaria|NZHYM2402-15|NZAC04099370|BOLD:ABX8626
Casinaria|NZHYM1556-12|NZAC04049373|BOLD:ABX9442
Casinaria|NZHYM2193-13|NZAC04087459|BOLD:ACB7919
Casinaria|NZHYM1955-12|NZAC04043270|BOLD:ACB7919
Casinaria|NZHYM1915-12|NZAC04043665|BOLD:ACB7919
Casinaria|NZICH087-20|NZAC03030213|BOLD:ACB7919
Casinaria|NZICH115-20|NZAC03030287|BOLD:ACB7919
Casinaria|NZICH305-20|NZAC03030091|BOLD:ACB7919
Casinaria|NZICH306-20|NZAC03030092|BOLD:ACB7919
Casinaria|NZICH295-20|NZAC03030081|BOLD:ACB7919
Casinaria|NZICH304-20|NZAC03030090|BOLD:ACB7919
Casinaria|NZICH116-20|NZAC03030288|BOLD:ACB7919
Casinaria|NZICH170-20|NZAC03034835|BOLD:ACB7919
Casinaria|NZICH248-20|NZAC03034896|BOLD:ACB7919
Casinaria|NZICH252-20|NZAC03034900|BOLD:ACB7919
Casinaria|NZICH265-20|NZAC03034913|BOLD:ACB7919
Casinaria|NZICH274-20|NZAC03034922|BOLD:ACB7919
Casinaria|NZHYM1941-12|NZAC04043708|BOLD:ACB7919
Casinaria|NZHYM1914-12|NZAC04043712|BOLD:ACB7919
Casinaria|NZHYM1938-12|NZAC04043931|BOLD:ACB7919
Casinaria|NZHYM1943-12|NZAC04044756|BOLD:ACB7919
Casinaria|NZHYM1946-12|NZAC04044843|BOLD:ACB7919
Casinaria|NZHYM1917-12|NZAC04044658|BOLD:ACB7919
Casinaria|NZHYM1913-12|NZAC04043834|BOLD:ACB7919
Casinaria|NZICH174-20|NZAC03034839|BOLD:ACB7919
Casinaria|NZICH340-20|NZAC03030126|BOLD:ACB7919
Casinaria|NZICH075-20|NZAC03030202|BOLD:ACB7919
Casinaria|NZICH370-20|NZAC03030169|BOLD:ACB7919
Casinaria|NZHYM1911-12|NZAC04043695|BOLD:ACB7919
Casinaria|LKMSC121-15|NZAC04105940|BOLD:ACB7919
Casinaria|LKMSC088-15|NZAC04106291|BOLD:ACB7919
Casinaria|LKMSC135-15|NZAC04106382|BOLD:ACB7919
Casinaria|LKMSC130-15|NZAC04106455|BOLD:ACB7919
Casinaria|NZHYM2403-15|NZAC04099202|BOLD:ACU4946
Casinaria|NZHYM3024-20|NZAC03025078|BOLD:AEG4805
Casinaria|NZHYM3022-20|NZAC03025079|BOLD:AEG4805
Casinaria|NZHYM3021-20|NZAC03025080|BOLD:AEG4805
Casinaria|NZHYM3023-20|NZAC03025081|BOLD:AEG4805
Casinaria|NZICH190-20|NZAC03034855|BOLD:AEG4805
Casinaria|UCICH114-14|CL-6987|BOLD:ACL9938
Casinaria|UCICH216-14|L-7628|BOLD:ACL9938
Casinaria|NZICH269-20|NZAC03034917|BOLD:AEH1899
Casinaria|LKMSC247-16|NZAC04137366|BOLD:ADE4132
Casinaria|LKMSC246-16|NZAC04138973|BOLD:ADE4132
Campoletis|NZHYM1880-12|NZAC04044494|BOLD:ACB7918
Campoletis|NZHYM1883-12|NZAC04043357|BOLD:ACB7918
Campoletis|NZICH285-20|NZAC03034933|BOLD:ACB7918
Campoletis|NZICH230-20|NZAC03034878|BOLD:ACB7918
Campoletis|NZICH227-20|NZAC03034875|BOLD:ACB7918
Campoletis|NZICH186-20|NZAC03034851|BOLD:ACB7918
Campoletis|NZHYM1957-12|NZAC04043841|BOLD:ACB7918
Campoletis|NZHYM1954-12|NZAC04044736|BOLD:ACB7918
Campoletis|UCICH277-14|M-391|BOLD:ACM0671
Campoletis|UCICH085-14|CL-5427|BOLD:ACB7004
Campoletis|UCICH153-14|L-3112|BOLD:ACB7004
Campoletis|UCICH361-14|M-9965|BOLD:ACB7004
Campoletis|NZHYM1882-12|NZAC04043427|BOLD:ACB7004
Campoletis|NZHYM1502-12|NZAC04048989|BOLD:ABX9024
Campoletis|NZHYM2473-15|NZAC04099975|BOLD:ABX9024
Campoletis|NZICH150-20|NZAC03034815|BOLD:ACB7721
Campoletis|NZHYM1878-12|NZAC04043608|BOLD:ACB7721
Campoletis|NZHYM1956-12|NZAC04043685|BOLD:ACB7721

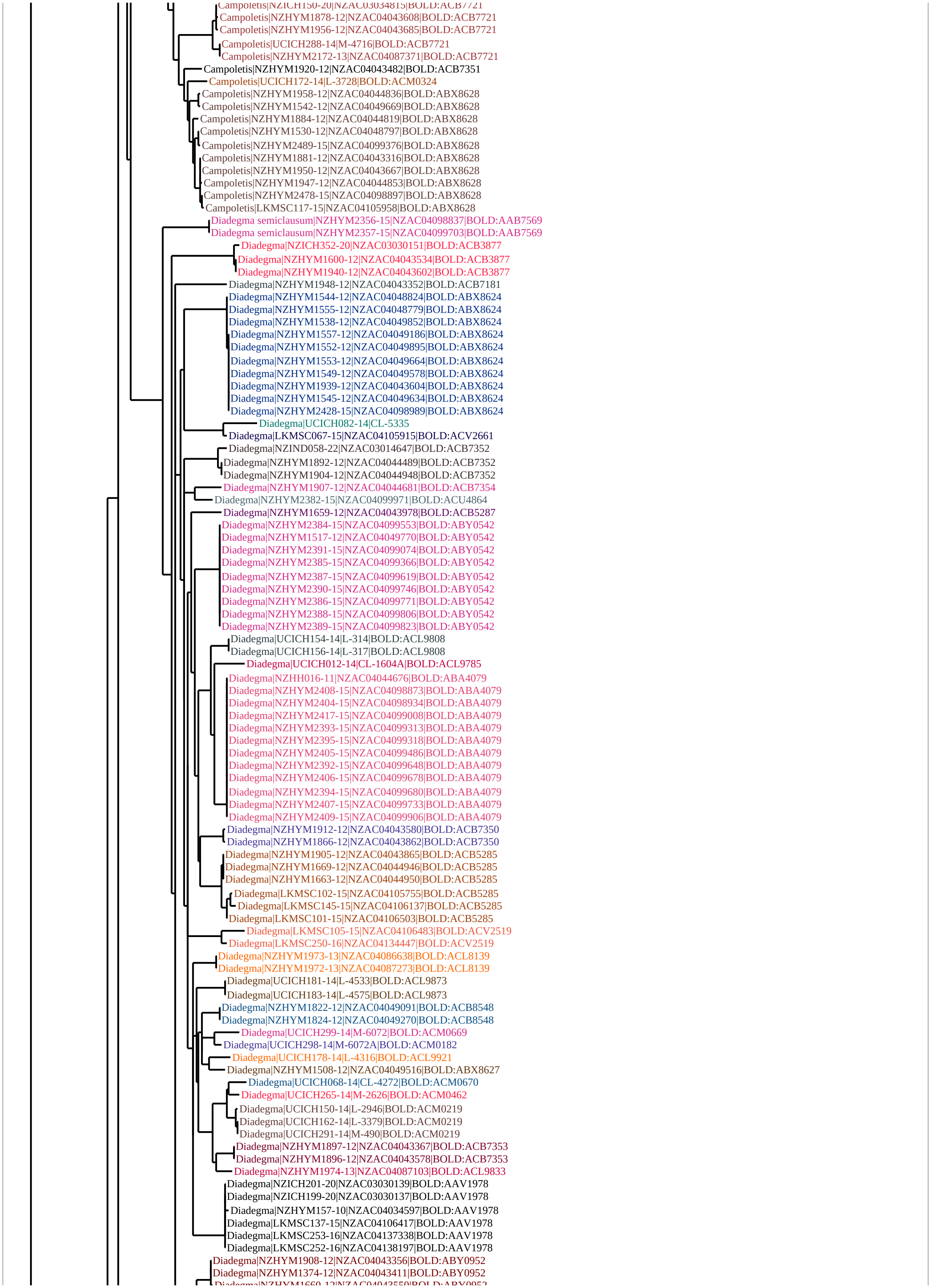
Campoletis|NZICH150-20|NZAC03034815|BOLD:ACB7721
Campoletis|NZHYM1878-12|NZAC04043608|BOLD:ACB7721
Campoletis|NZHYM1956-12|NZAC04043685|BOLD:ACB7721
Campoletis|UCICH288-14|M-4716|BOLD:ACB7721
Campoletis|NZHYM2172-13|NZAC04087371|BOLD:ACB7721
Campoletis|NZHYM1920-12|NZAC04043482|BOLD:ACB7351
Campoletis|UCICH172-14|L-3728|BOLD:ACM0324
Campoletis|NZHYM1958-12|NZAC04044836|BOLD:ABX8628
Campoletis|NZHYM1542-12|NZAC04049669|BOLD:ABX8628
Campoletis|NZHYM1884-12|NZAC04044819|BOLD:ABX8628
Campoletis|NZHYM1530-12|NZAC04048797|BOLD:ABX8628
Campoletis|NZHYM2489-15|NZAC04099376|BOLD:ABX8628
Campoletis|NZHYM1881-12|NZAC04043316|BOLD:ABX8628
Campoletis|NZHYM1950-12|NZAC04043667|BOLD:ABX8628
Campoletis|NZHYM1947-12|NZAC04044853|BOLD:ABX8628
Campoletis|NZHYM2478-15|NZAC04098897|BOLD:ABX8628
Campoletis|LKMSC117-15|NZAC04105958|BOLD:ABX8628
Diadegma semiclausum|NZHYM2356-15|NZAC04098837|BOLD:AAB7569
Diadegma semiclausum|NZHYM2357-15|NZAC04099703|BOLD:AAB7569
Diadegma|NZICH352-20|NZAC03030151|BOLD:ACB3877
Diadegma|NZHYM1600-12|NZAC04043534|BOLD:ACB3877
Diadegma|NZHYM1940-12|NZAC04043602|BOLD:ACB3877
Diadegma|NZHYM1948-12|NZAC04043352|BOLD:ACB7181
Diadegma|NZHYM1544-12|NZAC04048824|BOLD:ABX8624
Diadegma|NZHYM1555-12|NZAC04048779|BOLD:ABX8624
Diadegma|NZHYM1538-12|NZAC04049852|BOLD:ABX8624
Diadegma|NZHYM1557-12|NZAC04049186|BOLD:ABX8624
Diadegma|NZHYM1552-12|NZAC04049895|BOLD:ABX8624
Diadegma|NZHYM1553-12|NZAC04049664|BOLD:ABX8624
Diadegma|NZHYM1549-12|NZAC04049578|BOLD:ABX8624
Diadegma|NZHYM1939-12|NZAC04043604|BOLD:ABX8624
Diadegma|NZHYM1545-12|NZAC04049634|BOLD:ABX8624
Diadegma|NZHYM2428-15|NZAC04098989|BOLD:ABX8624
Diadegma|UCICH082-14|CL-5335
Diadegma|LKMSC067-15|NZAC04105915|BOLD:ACV2661
Diadegma|NZIND058-22|NZAC03014647|BOLD:ACB7352
Diadegma|NZHYM1892-12|NZAC04044489|BOLD:ACB7352
Diadegma|NZHYM1904-12|NZAC04044948|BOLD:ACB7352
Diadegma|NZHYM1907-12|NZAC04044681|BOLD:ACB7354
Diadegma|NZHYM2382-15|NZAC04099971|BOLD:ACU4864
Diadegma|NZHYM1659-12|NZAC04043978|BOLD:ACB5287
Diadegma|NZHYM2384-15|NZAC04099553|BOLD:ABY0542
Diadegma|NZHYM1517-12|NZAC04049770|BOLD:ABY0542
Diadegma|NZHYM2391-15|NZAC04099074|BOLD:ABY0542
Diadegma|NZHYM2385-15|NZAC04099366|BOLD:ABY0542
Diadegma|NZHYM2387-15|NZAC04099619|BOLD:ABY0542
Diadegma|NZHYM2390-15|NZAC04099746|BOLD:ABY0542
Diadegma|NZHYM2386-15|NZAC04099771|BOLD:ABY0542
Diadegma|NZHYM2388-15|NZAC04099806|BOLD:ABY0542
Diadegma|NZHYM2389-15|NZAC04099823|BOLD:ABY0542
Diadegma|UCICH154-14|L-314|BOLD:ACL9808
Diadegma|UCICH156-14|L-317|BOLD:ACL9808
Diadegma|UCICH012-14|CL-1604A|BOLD:ACL9785
Diadegma|NZHH016-11|NZAC04044676|BOLD:ABA4079
Diadegma|NZHYM2408-15|NZAC04098873|BOLD:ABA4079
Diadegma|NZHYM2404-15|NZAC04098934|BOLD:ABA4079
Diadegma|NZHYM2417-15|NZAC04099008|BOLD:ABA4079
Diadegma|NZHYM2393-15|NZAC04099313|BOLD:ABA4079
Diadegma|NZHYM2395-15|NZAC04099318|BOLD:ABA4079
Diadegma|NZHYM2405-15|NZAC04099486|BOLD:ABA4079
Diadegma|NZHYM2392-15|NZAC04099648|BOLD:ABA4079
Diadegma|NZHYM2406-15|NZAC04099678|BOLD:ABA4079
Diadegma|NZHYM2394-15|NZAC04099680|BOLD:ABA4079
Diadegma|NZHYM2407-15|NZAC04099733|BOLD:ABA4079
Diadegma|NZHYM2409-15|NZAC04099906|BOLD:ABA4079
Diadegma|NZHYM1912-12|NZAC04043580|BOLD:ACB7350
Diadegma|NZHYM1866-12|NZAC04043862|BOLD:ACB7350
Diadegma|NZHYM1905-12|NZAC04043865|BOLD:ACB5285
Diadegma|NZHYM1669-12|NZAC04044946|BOLD:ACB5285
Diadegma|NZHYM1663-12|NZAC04044950|BOLD:ACB5285
Diadegma|LKMSC102-15|NZAC04105755|BOLD:ACB5285
Diadegma|LKMSC145-15|NZAC04106137|BOLD:ACB5285
Diadegma|LKMSC101-15|NZAC04106503|BOLD:ACB5285
Diadegma|LKMSC105-15|NZAC04106483|BOLD:ACV2519
Diadegma|LKMSC250-16|NZAC04134447|BOLD:ACV2519
Diadegma|NZHYM1973-13|NZAC04086638|BOLD:ACL8139
Diadegma|NZHYM1972-13|NZAC04087273|BOLD:ACL8139
Diadegma|UCICH181-14|L-4533|BOLD:ACL9873
Diadegma|UCICH183-14|L-4575|BOLD:ACL9873
Diadegma|NZHYM1822-12|NZAC04049091|BOLD:ACB8548
Diadegma|NZHYM1824-12|NZAC04049270|BOLD:ACB8548
Diadegma|UCICH299-14|M-6072|BOLD:ACM0669
Diadegma|UCICH298-14|M-6072A|BOLD:ACM0182
Diadegma|UCICH178-14|L-4316|BOLD:ACL9921
Diadegma|NZHYM1508-12|NZAC04049516|BOLD:ABX8627
Diadegma|UCICH068-14|CL-4272|BOLD:ACM0670
Diadegma|UCICH265-14|M-2626|BOLD:ACM0462
Diadegma|UCICH150-14|L-2946|BOLD:ACM0219
Diadegma|UCICH162-14|L-3379|BOLD:ACM0219
Diadegma|UCICH291-14|M-490|BOLD:ACM0219
Diadegma|NZHYM1897-12|NZAC04043367|BOLD:ACB7353
Diadegma|NZHYM1896-12|NZAC04043578|BOLD:ACB7353
Diadegma|NZHYM1974-13|NZAC04087103|BOLD:ACL9833
Diadegma|NZICH201-20|NZAC03030139|BOLD:AAV1978
Diadegma|NZICH199-20|NZAC03030137|BOLD:AAV1978
Diadegma|NZHYM157-10|NZAC04034597|BOLD:AAV1978
Diadegma|LKMSC137-15|NZAC04106417|BOLD:AAV1978
Diadegma|LKMSC253-16|NZAC04137338|BOLD:AAV1978
Diadegma|LKMSC252-16|NZAC04138197|BOLD:AAV1978
Diadegma|NZHYM1908-12|NZAC04043356|BOLD:ABY0952
Diadegma|NZHYM1374-12|NZAC04043411|BOLD:ABY0952
Diadegma|NZHYM1660-12|NZAC04043550|BOLD:ABY0952

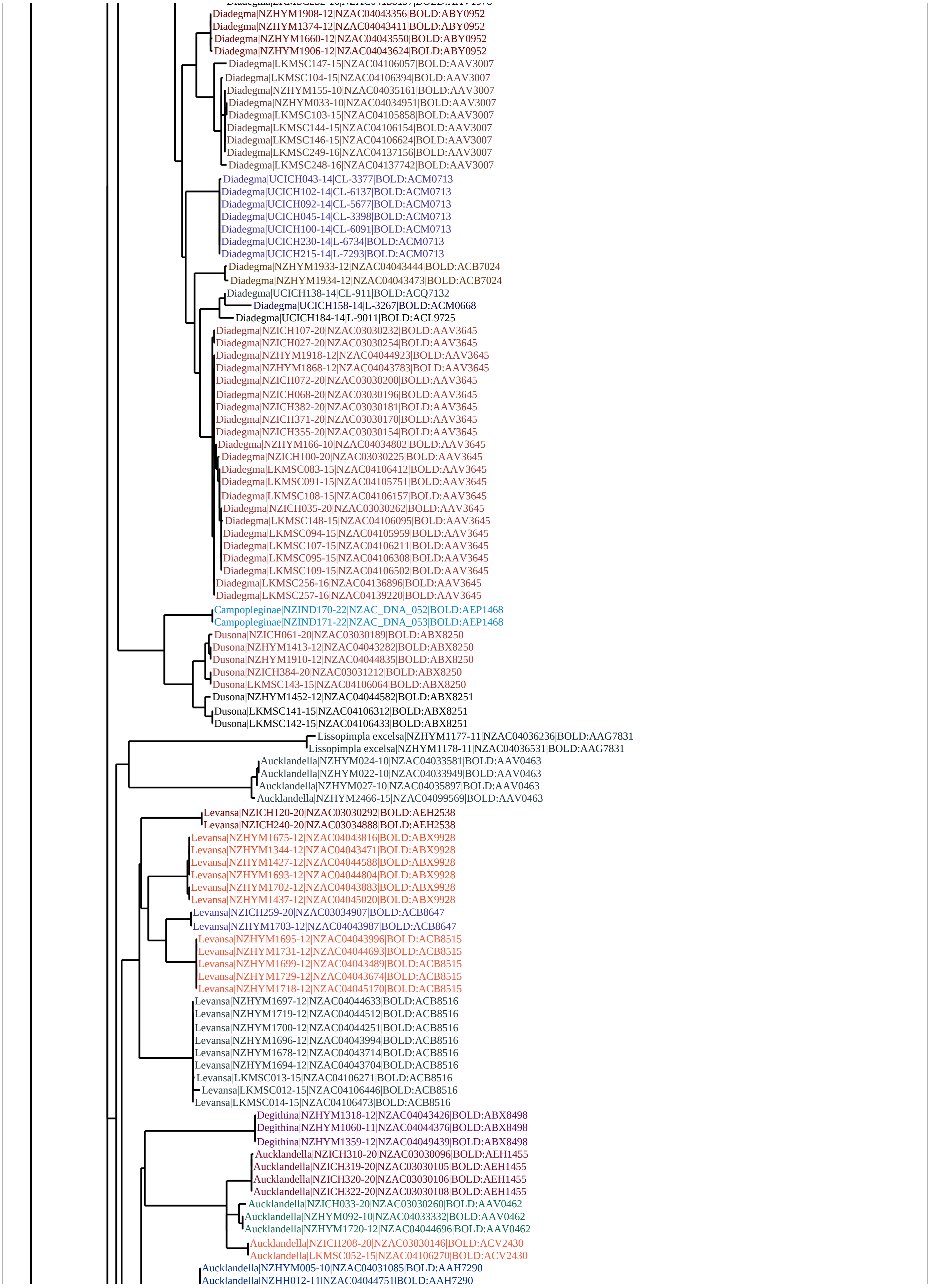
Diadegma|NZHYM1908-12|NZAC04043356|BOLD:ABY0952
Diadegma|NZHYM1374-12|NZAC04043411|BOLD:ABY0952
Diadegma|NZHYM1660-12|NZAC04043550|BOLD:ABY0952
Diadegma|NZHYM1906-12|NZAC04043624|BOLD:ABY0952
Diadegma|LKMSC147-15|NZAC04106057|BOLD:AAV3007
Diadegma|LKMSC104-15|NZAC04106394|BOLD:AAV3007
Diadegma|NZHYM155-10|NZAC04035161|BOLD:AAV3007
Diadegma|NZHYM033-10|NZAC04034951|BOLD:AAV3007
Diadegma|LKMSC103-15|NZAC04105858|BOLD:AAV3007
Diadegma|LKMSC144-15|NZAC04106154|BOLD:AAV3007
Diadegma|LKMSC146-15|NZAC04106624|BOLD:AAV3007
Diadegma|LKMSC249-16|NZAC04137156|BOLD:AAV3007
Diadegma|LKMSC248-16|NZAC04137742|BOLD:AAV3007
Diadegma|UCICH043-14|CL-3377|BOLD:ACM0713
Diadegma|UCICH102-14|CL-6137|BOLD:ACM0713
Diadegma|UCICH092-14|CL-5677|BOLD:ACM0713
Diadegma|UCICH045-14|CL-3398|BOLD:ACM0713
Diadegma|UCICH100-14|CL-6091|BOLD:ACM0713
Diadegma|UCICH230-14|L-6734|BOLD:ACM0713
Diadegma|UCICH215-14|L-7293|BOLD:ACM0713
Diadegma|NZHYM1933-12|NZAC04043444|BOLD:ACB7024
Diadegma|NZHYM1934-12|NZAC04043473|BOLD:ACB7024
Diadegma|UCICH138-14|CL-911|BOLD:ACQ7132
Diadegma|UCICH158-14|L-3267|BOLD:ACM0668
Diadegma|UCICH184-14|L-9011|BOLD:ACL9725
Diadegma|NZICH107-20|NZAC03030232|BOLD:AAV3645
Diadegma|NZICH027-20|NZAC03030254|BOLD:AAV3645
Diadegma|NZHYM1918-12|NZAC04044923|BOLD:AAV3645
Diadegma|NZHYM1868-12|NZAC04043783|BOLD:AAV3645
Diadegma|NZICH072-20|NZAC03030200|BOLD:AAV3645
Diadegma|NZICH068-20|NZAC03030196|BOLD:AAV3645
Diadegma|NZICH382-20|NZAC03030181|BOLD:AAV3645
Diadegma|NZICH371-20|NZAC03030170|BOLD:AAV3645
Diadegma|NZICH355-20|NZAC03030154|BOLD:AAV3645
Diadegma|NZHYM166-10|NZAC04034802|BOLD:AAV3645
Diadegma|NZICH100-20|NZAC03030225|BOLD:AAV3645
Diadegma|LKMSC083-15|NZAC04106412|BOLD:AAV3645
Diadegma|LKMSC091-15|NZAC04105751|BOLD:AAV3645
Diadegma|LKMSC108-15|NZAC04106157|BOLD:AAV3645
Diadegma|NZICH035-20|NZAC03030262|BOLD:AAV3645
Diadegma|LKMSC148-15|NZAC04106095|BOLD:AAV3645
Diadegma|LKMSC094-15|NZAC04105959|BOLD:AAV3645
Diadegma|LKMSC107-15|NZAC04106211|BOLD:AAV3645
Diadegma|LKMSC095-15|NZAC04106308|BOLD:AAV3645
Diadegma|LKMSC109-15|NZAC04106502|BOLD:AAV3645
Diadegma|LKMSC256-16|NZAC04136896|BOLD:AAV3645
Diadegma|LKMSC257-16|NZAC04139220|BOLD:AAV3645
Campopleginae|NZIND170-22|NZAC_DNA_052|BOLD:AEP1468
Campopleginae|NZIND171-22|NZAC_DNA_053|BOLD:AEP1468
Dusona|NZICH061-20|NZAC03030189|BOLD:ABX8250
Dusona|NZHYM1413-12|NZAC04043282|BOLD:ABX8250
Dusona|NZHYM1910-12|NZAC04044835|BOLD:ABX8250
Dusona|NZICH384-20|NZAC03031212|BOLD:ABX8250
Dusona|LKMSC143-15|NZAC04106064|BOLD:ABX8250
Dusona|NZHYM1452-12|NZAC04044582|BOLD:ABX8251
Dusona|LKMSC141-15|NZAC04106312|BOLD:ABX8251
Dusona|LKMSC142-15|NZAC04106433|BOLD:ABX8251
Lissopimpla excelsa|NZHYM1177-11|NZAC04036236|BOLD:AAG7831
Lissopimpla excelsa|NZHYM1178-11|NZAC04036531|BOLD:AAG7831
Aucklandella|NZHYM024-10|NZAC04033581|BOLD:AAV0463
Aucklandella|NZHYM022-10|NZAC04033949|BOLD:AAV0463
Aucklandella|NZHYM027-10|NZAC04035897|BOLD:AAV0463
Aucklandella|NZHYM2466-15|NZAC04099569|BOLD:AAV0463
Levansa|NZICH120-20|NZAC03030292|BOLD:AEH2538
Levansa|NZICH240-20|NZAC03034888|BOLD:AEH2538
Levansa|NZHYM1675-12|NZAC04043816|BOLD:ABX9928
Levansa|NZHYM1344-12|NZAC04043471|BOLD:ABX9928
Levansa|NZHYM1427-12|NZAC04044588|BOLD:ABX9928
Levansa|NZHYM1693-12|NZAC04044804|BOLD:ABX9928
Levansa|NZHYM1702-12|NZAC04043883|BOLD:ABX9928
Levansa|NZHYM1437-12|NZAC04045020|BOLD:ABX9928
Levansa|NZICH259-20|NZAC03034907|BOLD:ACB8647
Levansa|NZHYM1703-12|NZAC04043987|BOLD:ACB8647
Levansa|NZHYM1695-12|NZAC04043996|BOLD:ACB8515
Levansa|NZHYM1731-12|NZAC04044693|BOLD:ACB8515
Levansa|NZHYM1699-12|NZAC04043489|BOLD:ACB8515
Levansa|NZHYM1729-12|NZAC04043674|BOLD:ACB8515
Levansa|NZHYM1718-12|NZAC04045170|BOLD:ACB8515
Levansa|NZHYM1697-12|NZAC04044633|BOLD:ACB8516
Levansa|NZHYM1719-12|NZAC04044512|BOLD:ACB8516
Levansa|NZHYM1700-12|NZAC04044251|BOLD:ACB8516
Levansa|NZHYM1696-12|NZAC04043994|BOLD:ACB8516
Levansa|NZHYM1678-12|NZAC04043714|BOLD:ACB8516
Levansa|NZHYM1694-12|NZAC04043704|BOLD:ACB8516
Levansa|LKMSC013-15|NZAC04106271|BOLD:ACB8516
Levansa|LKMSC012-15|NZAC04106446|BOLD:ACB8516
Levansa|LKMSC014-15|NZAC04106473|BOLD:ACB8516
Degithina|NZHYM1318-12|NZAC04043426|BOLD:ABX8498
Degithina|NZHYM1060-11|NZAC04044376|BOLD:ABX8498
Degithina|NZHYM1359-12|NZAC04049439|BOLD:ABX8498
Aucklandella|NZICH310-20|NZAC03030096|BOLD:AEH1455
Aucklandella|NZICH319-20|NZAC03030105|BOLD:AEH1455
Aucklandella|NZICH320-20|NZAC03030106|BOLD:AEH1455
Aucklandella|NZICH322-20|NZAC03030108|BOLD:AEH1455
Aucklandella|NZICH033-20|NZAC03030260|BOLD:AAV0462
Aucklandella|NZHYM092-10|NZAC04033332|BOLD:AAV0462
Aucklandella|NZHYM1720-12|NZAC04044696|BOLD:AAV0462
Aucklandella|NZICH208-20|NZAC03030146|BOLD:ACV2430
Aucklandella|LKMSC052-15|NZAC04106270|BOLD:ACV2430
Aucklandella|NZHYM005-10|NZAC04031085|BOLD:AAH7290
Aucklandella|NZHH012-11|NZAC04044751|BOLD:AAH7290

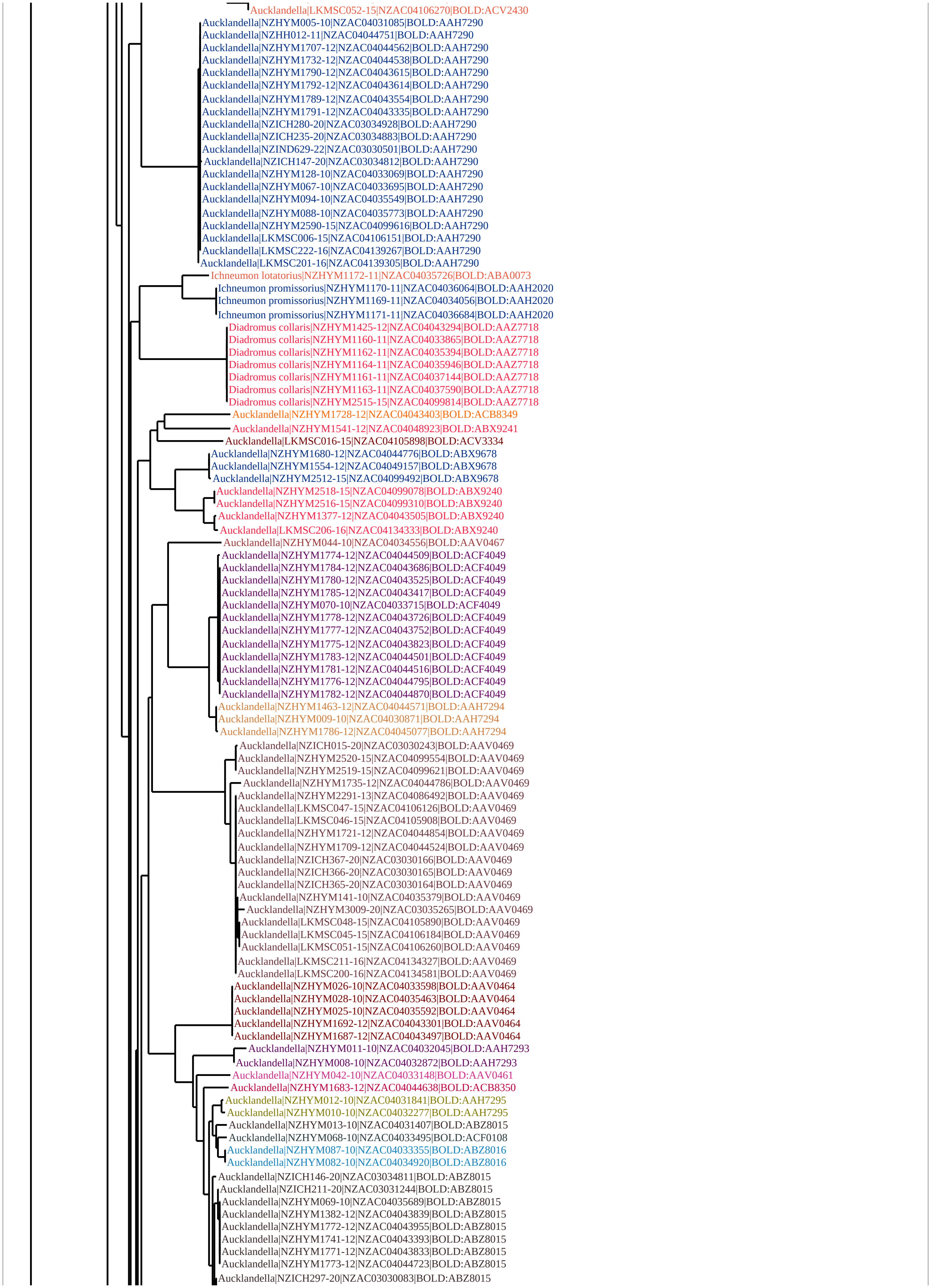
Aucklandella|LKMSC052-15|NZAC04106270|BOLD:ACV2430
Aucklandella|NZHYM005-10|NZAC04031085|BOLD:AAH7290
Aucklandella|NZHH012-11|NZAC04044751|BOLD:AAH7290
Aucklandella|NZHYM1707-12|NZAC04044562|BOLD:AAH7290
Aucklandella|NZHYM1732-12|NZAC04044538|BOLD:AAH7290
Aucklandella|NZHYM1790-12|NZAC04043615|BOLD:AAH7290
Aucklandella|NZHYM1792-12|NZAC04043614|BOLD:AAH7290
Aucklandella|NZHYM1789-12|NZAC04043554|BOLD:AAH7290
Aucklandella|NZHYM1791-12|NZAC04043335|BOLD:AAH7290
Aucklandella|NZICH280-20|NZAC03034928|BOLD:AAH7290
Aucklandella|NZICH235-20|NZAC03034883|BOLD:AAH7290
Aucklandella|NZIND629-22|NZAC03030501|BOLD:AAH7290
Aucklandella|NZICH147-20|NZAC03034812|BOLD:AAH7290
Aucklandella|NZHYM128-10|NZAC04033069|BOLD:AAH7290
Aucklandella|NZHYM067-10|NZAC04033695|BOLD:AAH7290
Aucklandella|NZHYM094-10|NZAC04035549|BOLD:AAH7290
Aucklandella|NZHYM088-10|NZAC04035773|BOLD:AAH7290
Aucklandella|NZHYM2590-15|NZAC04099616|BOLD:AAH7290
Aucklandella|LKMSC006-15|NZAC04106151|BOLD:AAH7290
Aucklandella|LKMSC222-16|NZAC04139267|BOLD:AAH7290
Aucklandella|LKMSC201-16|NZAC04139305|BOLD:AAH7290
Ichneumon lotatorius|NZHYM1172-11|NZAC04035726|BOLD:ABA0073
Ichneumon promissorius|NZHYM1170-11|NZAC04036064|BOLD:AAH2020
Ichneumon promissorius|NZHYM1169-11|NZAC04034056|BOLD:AAH2020
Ichneumon promissorius|NZHYM1171-11|NZAC04036684|BOLD:AAH2020
Diadromus collaris|NZHYM1425-12|NZAC04043294|BOLD:AAZ7718
Diadromus collaris|NZHYM1160-11|NZAC04033865|BOLD:AAZ7718
Diadromus collaris|NZHYM1162-11|NZAC04035394|BOLD:AAZ7718
Diadromus collaris|NZHYM1164-11|NZAC04035946|BOLD:AAZ7718
Diadromus collaris|NZHYM1161-11|NZAC04037144|BOLD:AAZ7718
Diadromus collaris|NZHYM1163-11|NZAC04037590|BOLD:AAZ7718
Diadromus collaris|NZHYM2515-15|NZAC04099814|BOLD:AAZ7718
Aucklandella|NZHYM1728-12|NZAC04043403|BOLD:ACB8349
Aucklandella|NZHYM1541-12|NZAC04048923|BOLD:ABX9241
Aucklandella|LKMSC016-15|NZAC04105898|BOLD:ACV3334
Aucklandella|NZHYM1680-12|NZAC04044776|BOLD:ABX9678
Aucklandella|NZHYM1554-12|NZAC04049157|BOLD:ABX9678
Aucklandella|NZHYM2512-15|NZAC04099492|BOLD:ABX9678
Aucklandella|NZHYM2518-15|NZAC04099078|BOLD:ABX9240
Aucklandella|NZHYM2516-15|NZAC04099310|BOLD:ABX9240
Aucklandella|NZHYM1377-12|NZAC04043505|BOLD:ABX9240
Aucklandella|LKMSC206-16|NZAC04134333|BOLD:ABX9240
Aucklandella|NZHYM044-10|NZAC04034556|BOLD:AAV0467
Aucklandella|NZHYM1774-12|NZAC04044509|BOLD:ACF4049
Aucklandella|NZHYM1784-12|NZAC04043686|BOLD:ACF4049
Aucklandella|NZHYM1780-12|NZAC04043525|BOLD:ACF4049
Aucklandella|NZHYM1785-12|NZAC04043417|BOLD:ACF4049
Aucklandella|NZHYM070-10|NZAC04033715|BOLD:ACF4049
Aucklandella|NZHYM1778-12|NZAC04043726|BOLD:ACF4049
Aucklandella|NZHYM1777-12|NZAC04043752|BOLD:ACF4049
Aucklandella|NZHYM1775-12|NZAC04043823|BOLD:ACF4049
Aucklandella|NZHYM1783-12|NZAC04044501|BOLD:ACF4049
Aucklandella|NZHYM1781-12|NZAC04044516|BOLD:ACF4049
Aucklandella|NZHYM1776-12|NZAC04044795|BOLD:ACF4049
Aucklandella|NZHYM1782-12|NZAC04044870|BOLD:ACF4049
Aucklandella|NZHYM1463-12|NZAC04044571|BOLD:AAH7294
Aucklandella|NZHYM009-10|NZAC04030871|BOLD:AAH7294
Aucklandella|NZHYM1786-12|NZAC04045077|BOLD:AAH7294
Aucklandella|NZICH015-20|NZAC03030243|BOLD:AAV0469
Aucklandella|NZHYM2520-15|NZAC04099554|BOLD:AAV0469
Aucklandella|NZHYM2519-15|NZAC04099621|BOLD:AAV0469
Aucklandella|NZHYM1735-12|NZAC04044786|BOLD:AAV0469
Aucklandella|NZHYM2291-13|NZAC04086492|BOLD:AAV0469
Aucklandella|LKMSC047-15|NZAC04106126|BOLD:AAV0469
Aucklandella|LKMSC046-15|NZAC04105908|BOLD:AAV0469
Aucklandella|NZHYM1721-12|NZAC04044854|BOLD:AAV0469
Aucklandella|NZHYM1709-12|NZAC04044524|BOLD:AAV0469
Aucklandella|NZICH367-20|NZAC03030166|BOLD:AAV0469
Aucklandella|NZICH366-20|NZAC03030165|BOLD:AAV0469
Aucklandella|NZICH365-20|NZAC03030164|BOLD:AAV0469
Aucklandella|NZHYM141-10|NZAC04035379|BOLD:AAV0469
Aucklandella|NZHYM3009-20|NZAC03035265|BOLD:AAV0469
Aucklandella|LKMSC048-15|NZAC04105890|BOLD:AAV0469
Aucklandella|LKMSC045-15|NZAC04106184|BOLD:AAV0469
Aucklandella|LKMSC051-15|NZAC04106260|BOLD:AAV0469
Aucklandella|LKMSC211-16|NZAC04134327|BOLD:AAV0469
Aucklandella|LKMSC200-16|NZAC04134581|BOLD:AAV0469
Aucklandella|NZHYM026-10|NZAC04033598|BOLD:AAV0464
Aucklandella|NZHYM028-10|NZAC04035463|BOLD:AAV0464
Aucklandella|NZHYM025-10|NZAC04035592|BOLD:AAV0464
Aucklandella|NZHYM1692-12|NZAC04043301|BOLD:AAV0464
Aucklandella|NZHYM1687-12|NZAC04043497|BOLD:AAV0464
Aucklandella|NZHYM011-10|NZAC04032045|BOLD:AAH7293
Aucklandella|NZHYM008-10|NZAC04032872|BOLD:AAH7293
Aucklandella|NZHYM042-10|NZAC04033148|BOLD:AAV0461
Aucklandella|NZHYM1683-12|NZAC04044638|BOLD:ACB8350
Aucklandella|NZHYM012-10|NZAC04031841|BOLD:AAH7295
Aucklandella|NZHYM010-10|NZAC04032277|BOLD:AAH7295
Aucklandella|NZHYM013-10|NZAC04031407|BOLD:ABZ8015
Aucklandella|NZHYM068-10|NZAC04033495|BOLD:ACF0108
Aucklandella|NZHYM087-10|NZAC04033355|BOLD:ABZ8016
Aucklandella|NZHYM082-10|NZAC04034920|BOLD:ABZ8016
Aucklandella|NZICH146-20|NZAC03034811|BOLD:ABZ8015
Aucklandella|NZICH211-20|NZAC03031244|BOLD:ABZ8015
Aucklandella|NZHYM069-10|NZAC04035689|BOLD:ABZ8015
Aucklandella|NZHYM1382-12|NZAC04043839|BOLD:ABZ8015
Aucklandella|NZHYM1772-12|NZAC04043955|BOLD:ABZ8015
Aucklandella|NZHYM1741-12|NZAC04043393|BOLD:ABZ8015
Aucklandella|NZHYM1771-12|NZAC04043833|BOLD:ABZ8015
Aucklandella|NZHYM1773-12|NZAC04044723|BOLD:ABZ8015
Aucklandella|NZICH297-20|NZAC03030083|BOLD:ABZ8015

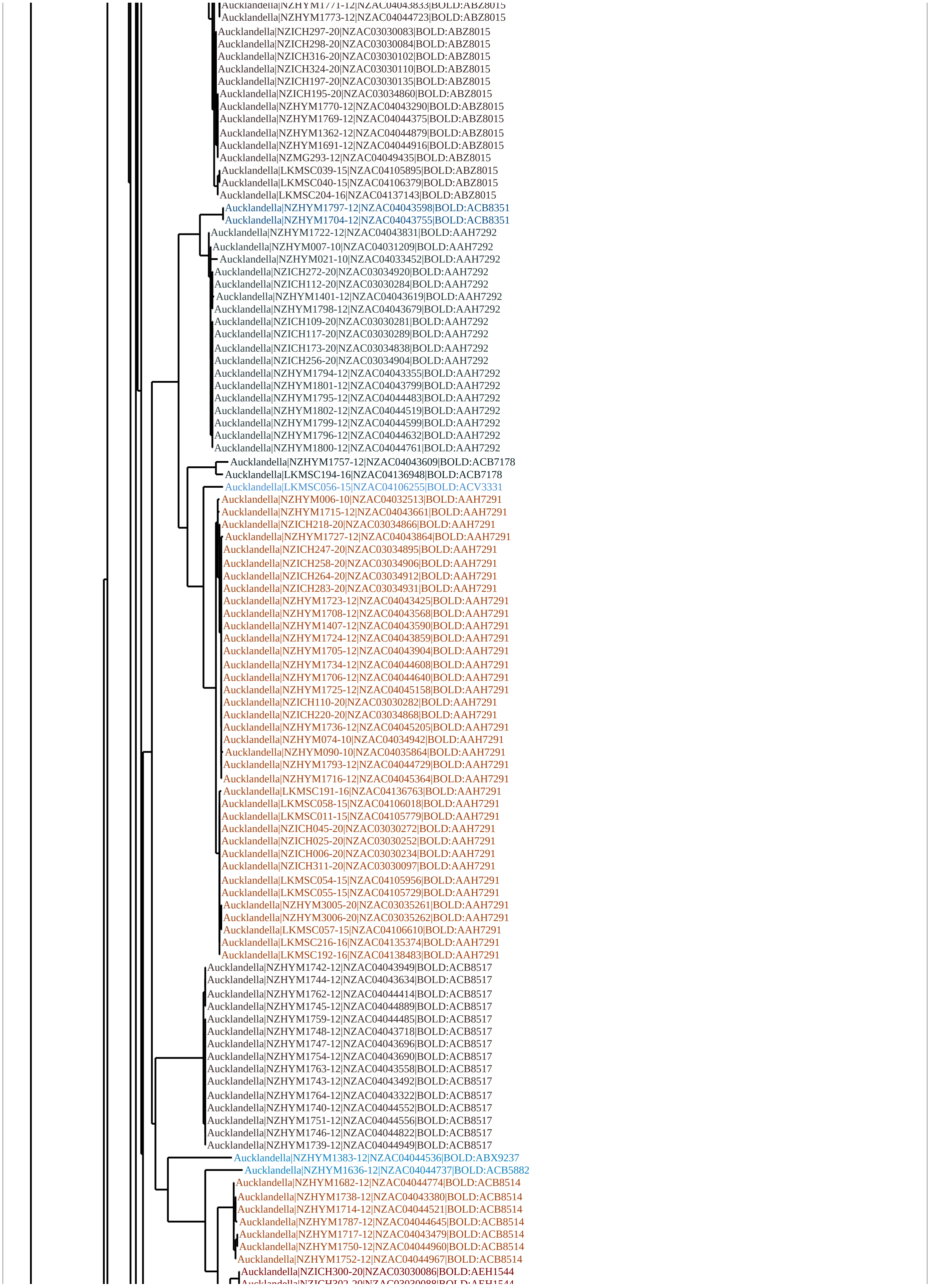
Aucklandella|NZHYM1771-12|NZAC04043833|BOLD:ABZ8015
Aucklandella|NZHYM1773-12|NZAC04044723|BOLD:ABZ8015
Aucklandella|NZICH297-20|NZAC03030083|BOLD:ABZ8015
Aucklandella|NZICH298-20|NZAC03030084|BOLD:ABZ8015
Aucklandella|NZICH316-20|NZAC03030102|BOLD:ABZ8015
Aucklandella|NZICH324-20|NZAC03030110|BOLD:ABZ8015
Aucklandella|NZICH197-20|NZAC03030135|BOLD:ABZ8015
Aucklandella|NZICH195-20|NZAC03034860|BOLD:ABZ8015
Aucklandella|NZHYM1770-12|NZAC04043290|BOLD:ABZ8015
Aucklandella|NZHYM1769-12|NZAC04044375|BOLD:ABZ8015
Aucklandella|NZHYM1362-12|NZAC04044879|BOLD:ABZ8015
Aucklandella|NZHYM1691-12|NZAC04044916|BOLD:ABZ8015
Aucklandella|NZMG293-12|NZAC04049435|BOLD:ABZ8015
Aucklandella|LKMSC039-15|NZAC04105895|BOLD:ABZ8015
Aucklandella|LKMSC040-15|NZAC04106379|BOLD:ABZ8015
Aucklandella|LKMSC204-16|NZAC04137143|BOLD:ABZ8015
Aucklandella|NZHYM1797-12|NZAC04043598|BOLD:ACB8351
Aucklandella|NZHYM1704-12|NZAC04043755|BOLD:ACB8351
Aucklandella|NZHYM1722-12|NZAC04043831|BOLD:AAH7292
Aucklandella|NZHYM007-10|NZAC04031209|BOLD:AAH7292
Aucklandella|NZHYM021-10|NZAC04033452|BOLD:AAH7292
Aucklandella|NZICH272-20|NZAC03034920|BOLD:AAH7292
Aucklandella|NZICH112-20|NZAC03030284|BOLD:AAH7292
Aucklandella|NZHYM1401-12|NZAC04043619|BOLD:AAH7292
Aucklandella|NZHYM1798-12|NZAC04043679|BOLD:AAH7292
Aucklandella|NZICH109-20|NZAC03030281|BOLD:AAH7292
Aucklandella|NZICH117-20|NZAC03030289|BOLD:AAH7292
Aucklandella|NZICH173-20|NZAC03034838|BOLD:AAH7292
Aucklandella|NZICH256-20|NZAC03034904|BOLD:AAH7292
Aucklandella|NZHYM1794-12|NZAC04043355|BOLD:AAH7292
Aucklandella|NZHYM1801-12|NZAC04043799|BOLD:AAH7292
Aucklandella|NZHYM1795-12|NZAC04044483|BOLD:AAH7292
Aucklandella|NZHYM1802-12|NZAC04044519|BOLD:AAH7292
Aucklandella|NZHYM1799-12|NZAC04044599|BOLD:AAH7292
Aucklandella|NZHYM1796-12|NZAC04044632|BOLD:AAH7292
Aucklandella|NZHYM1800-12|NZAC04044761|BOLD:AAH7292
Aucklandella|NZHYM1757-12|NZAC04043609|BOLD:ACB7178
Aucklandella|LKMSC194-16|NZAC04136948|BOLD:ACB7178
Aucklandella|LKMSC056-15|NZAC04106255|BOLD:ACV3331
Aucklandella|NZHYM006-10|NZAC04032513|BOLD:AAH7291
Aucklandella|NZHYM1715-12|NZAC04043661|BOLD:AAH7291
Aucklandella|NZICH218-20|NZAC03034866|BOLD:AAH7291
Aucklandella|NZHYM1727-12|NZAC04043864|BOLD:AAH7291
Aucklandella|NZICH247-20|NZAC03034895|BOLD:AAH7291
Aucklandella|NZICH258-20|NZAC03034906|BOLD:AAH7291
Aucklandella|NZICH264-20|NZAC03034912|BOLD:AAH7291
Aucklandella|NZICH283-20|NZAC03034931|BOLD:AAH7291
Aucklandella|NZHYM1723-12|NZAC04043425|BOLD:AAH7291
Aucklandella|NZHYM1708-12|NZAC04043568|BOLD:AAH7291
Aucklandella|NZHYM1407-12|NZAC04043590|BOLD:AAH7291
Aucklandella|NZHYM1724-12|NZAC04043859|BOLD:AAH7291
Aucklandella|NZHYM1705-12|NZAC04043904|BOLD:AAH7291
Aucklandella|NZHYM1734-12|NZAC04044608|BOLD:AAH7291
Aucklandella|NZHYM1706-12|NZAC04044640|BOLD:AAH7291
Aucklandella|NZHYM1725-12|NZAC04045158|BOLD:AAH7291
Aucklandella|NZICH110-20|NZAC03030282|BOLD:AAH7291
Aucklandella|NZICH220-20|NZAC03034868|BOLD:AAH7291
Aucklandella|NZHYM1736-12|NZAC04045205|BOLD:AAH7291
Aucklandella|NZHYM074-10|NZAC04034942|BOLD:AAH7291
Aucklandella|NZHYM090-10|NZAC04035864|BOLD:AAH7291
Aucklandella|NZHYM1793-12|NZAC04044729|BOLD:AAH7291
Aucklandella|NZHYM1716-12|NZAC04045364|BOLD:AAH7291
Aucklandella|LKMSC191-16|NZAC04136763|BOLD:AAH7291
Aucklandella|LKMSC058-15|NZAC04106018|BOLD:AAH7291
Aucklandella|LKMSC011-15|NZAC04105779|BOLD:AAH7291
Aucklandella|NZICH045-20|NZAC03030272|BOLD:AAH7291
Aucklandella|NZICH025-20|NZAC03030252|BOLD:AAH7291
Aucklandella|NZICH006-20|NZAC03030234|BOLD:AAH7291
Aucklandella|NZICH311-20|NZAC03030097|BOLD:AAH7291
Aucklandella|LKMSC054-15|NZAC04105956|BOLD:AAH7291
Aucklandella|LKMSC055-15|NZAC04105729|BOLD:AAH7291
Aucklandella|NZHYM3005-20|NZAC03035261|BOLD:AAH7291
Aucklandella|NZHYM3006-20|NZAC03035262|BOLD:AAH7291
Aucklandella|LKMSC057-15|NZAC04106610|BOLD:AAH7291
Aucklandella|LKMSC216-16|NZAC04135374|BOLD:AAH7291
Aucklandella|LKMSC192-16|NZAC04138483|BOLD:AAH7291
Aucklandella|NZHYM1742-12|NZAC04043949|BOLD:ACB8517
Aucklandella|NZHYM1744-12|NZAC04043634|BOLD:ACB8517
Aucklandella|NZHYM1762-12|NZAC04044414|BOLD:ACB8517
Aucklandella|NZHYM1745-12|NZAC04044889|BOLD:ACB8517
Aucklandella|NZHYM1759-12|NZAC04044485|BOLD:ACB8517
Aucklandella|NZHYM1748-12|NZAC04043718|BOLD:ACB8517
Aucklandella|NZHYM1747-12|NZAC04043696|BOLD:ACB8517
Aucklandella|NZHYM1754-12|NZAC04043690|BOLD:ACB8517
Aucklandella|NZHYM1763-12|NZAC04043558|BOLD:ACB8517
Aucklandella|NZHYM1743-12|NZAC04043492|BOLD:ACB8517
Aucklandella|NZHYM1764-12|NZAC04043322|BOLD:ACB8517
Aucklandella|NZHYM1740-12|NZAC04044552|BOLD:ACB8517
Aucklandella|NZHYM1751-12|NZAC04044556|BOLD:ACB8517
Aucklandella|NZHYM1746-12|NZAC04044822|BOLD:ACB8517
Aucklandella|NZHYM1739-12|NZAC04044949|BOLD:ACB8517
Aucklandella|NZHYM1383-12|NZAC04044536|BOLD:ABX9237
Aucklandella|NZHYM1636-12|NZAC04044737|BOLD:ACB5882
Aucklandella|NZHYM1682-12|NZAC04044774|BOLD:ACB8514
Aucklandella|NZHYM1738-12|NZAC04043380|BOLD:ACB8514
Aucklandella|NZHYM1714-12|NZAC04044521|BOLD:ACB8514
Aucklandella|NZHYM1787-12|NZAC04044645|BOLD:ACB8514
Aucklandella|NZHYM1717-12|NZAC04043479|BOLD:ACB8514
Aucklandella|NZHYM1750-12|NZAC04044960|BOLD:ACB8514
Aucklandella|NZHYM1752-12|NZAC04044967|BOLD:ACB8514
Aucklandella|NZICH300-20|NZAC03030086|BOLD:AEH1544

[illegible]
Aucklandella|NZHYM1752-12|NZAC04044967|BOLD:ACB8514
Aucklandella|NZICH300-20|NZAC03030086|BOLD:AEH1544
Aucklandella|NZICH302-20|NZAC03030088|BOLD:AEH1544
Aucklandella|NZICH143-20|NZAC03031382|BOLD:AEH1544
Aucklandella|NZHYM060-10|NZAC04034010|BOLD:AAV0465
Aucklandella|LKMSC028-15|NZAC04106143|BOLD:ACP7239
Aucklandella|NZHYM2592-15|NZAC04099122|BOLD:ACP7239
Aucklandella|LKMSC218-16|NZAC04139241|BOLD:ACP7239
Aucklandella|LKMSC041-15|NZAC04106023|BOLD:ACP7239
Aucklandella|LKMSC050-15|NZAC04105914|BOLD:ACP7239
Aucklandella|LKMSC029-15|NZAC04105963|BOLD:ACP7239
Aucklandella|LKMSC042-15|NZAC04106040|BOLD:ACP7239
Aucklandella|LKMSC031-15|NZAC04106169|BOLD:ACP7239
Aucklandella|LKMSC219-16|NZAC04137324|BOLD:ACP7239
Aucklandella|LKMSC213-16|NZAC04138795|BOLD:ACP7239
Aucklandella|LKMSC212-16|NZAC04135118|BOLD:ACP7239
Aucklandella|NZICH080-20|NZAC03030207|BOLD:ACP7239
Aucklandella|LKMSC197-16|NZAC04139386|BOLD:ACP7239
Aucklandella|LKMSC196-16|NZAC04139544|BOLD:ACP7239
Aucklandella|LKMSC035-15|NZAC04106027|BOLD:ACV3332
Aucklandella|LKMSC034-15|NZAC04105809|BOLD:ACV3332
Aucklandella|LKMSC224-16|NZAC04136203|BOLD:ACV3332
Lusius|NZHYM1330-12|NZAC04044276|BOLD:ABX9664
Lusius|NZHYM1408-12|NZAC04043531|BOLD:ABX9664
Lusius|NZHYM1350-12|NZAC04044699|BOLD:ABX9664
Lusius|NZHYM1415-12|NZAC04044703|BOLD:ABX9664
Aucklandella|LKMSC019-15|NZAC04106289|BOLD:ABX9239
Aucklandella|LKMSC017-15|NZAC04105874|BOLD:ABX9239
Aucklandella|NZHYM1768-12|NZAC04044574|BOLD:ABX9239
Aucklandella|NZHYM1710-12|NZAC04043741|BOLD:ABX9239
Aucklandella|NZICH231-20|NZAC03034879|BOLD:ABX9239
Aucklandella|NZICH229-20|NZAC03034877|BOLD:ABX9239
Aucklandella|NZICH226-20|NZAC03034874|BOLD:ABX9239
Aucklandella|NZICH169-20|NZAC03034834|BOLD:ABX9239
Aucklandella|NZICH156-20|NZAC03034821|BOLD:ABX9239
Aucklandella|NZICH126-20|NZAC03030298|BOLD:ABX9239
Aucklandella|NZHYM1417-12|NZAC04044610|BOLD:ABX9239
Aucklandella|NZICH158-20|NZAC03034823|BOLD:ABX9239
Aucklandella|NZHYM1390-12|NZAC04043374|BOLD:ABX9239
Aucklandella|NZHYM1688-12|NZAC04044635|BOLD:ABX9239
Aucklandella|NZHYM1685-12|NZAC04044742|BOLD:ABX9239
Aucklandella|LKMSC225-16|NZAC04136883|BOLD:ABX9239
Aucklandella|LKMSC195-16|NZAC04138012|BOLD:ABX9239
Aucklandella|LKMSC021-15|NZAC04106177|BOLD:ABX9239
Aucklandella|LKMSC018-15|NZAC04106178|BOLD:ABX9239
Aucklandella|LKMSC044-15|NZAC04106240|BOLD:ABX9239
Aucklandella|LKMSC020-15|NZAC04106131|BOLD:ABX9239
Aucklandella|LKMSC043-15|NZAC04106276|BOLD:ABX9239
Aucklandella|LKMSC202-16|NZAC04138579|BOLD:ABX9239
Aucklandella|LKMSC203-16|NZAC04139702|BOLD:ABX9239
Degithina|NZHYM1428-12|NZAC04043902|BOLD:AAV1460
Degithina|NZHYM1338-12|NZAC04044809|BOLD:AAV1460
Degithina|NZHYM1337-12|NZAC04044759|BOLD:AAV1460
Degithina|NZHYM1348-12|NZAC04044590|BOLD:AAV1460
Degithina|NZHYM1307-12|NZAC04043769|BOLD:AAV1460
Degithina|NZHYM1319-12|NZAC04043543|BOLD:AAV1460
Degithina|NZICH270-20|NZAC03034918|BOLD:AAV1460
Degithina|NZICH192-20|NZAC03034857|BOLD:AAV1460
Degithina|NZICH165-20|NZAC03034830|BOLD:AAV1460
Degithina|NZICH151-20|NZAC03034816|BOLD:AAV1460
Degithina|NZHYM1272-12|NZAC04043894|BOLD:AAV1460
Degithina|NZHYM085-10|NZAC04033942|BOLD:AAV1460
Degithina|NZHYM1099-11|NZAC04045287
Degithina|NZHYM1120-11|NZAC04045385|BOLD:ABX9510
Degithina|NZHYM1357-12|NZAC04049153|BOLD:ABX9510
Degithina|NZHYM1434-12|NZAC04044611|BOLD:ABX9931
Degithina|NZHYM1296-12|NZAC04044733|BOLD:ABX9931
Degithina|NZHYM1297-12|NZAC04044867|BOLD:ABX9931
Degithina decepta|NZHYM1122-11|NZAC04044268|BOLD:ABX9929
Degithina|NZHYM1361-12|NZAC04048861|BOLD:ABX9929
Degithina|NZHYM1360-12|NZAC04049352|BOLD:ABX9929
Degithina|NZICH268-20|NZAC03034916|BOLD:ABX9926
Degithina|NZICH255-20|NZAC03034903|BOLD:ABX9926
Degithina|NZHYM1406-12|NZAC04043526|BOLD:ABX9926
Degithina|NZHYM1131-11|NZAC04045162|BOLD:ABX9926
Degithina|LKMSC004-15|NZAC04105953|BOLD:ABX9926
Degithina|NZICH385-20|NZAC03038650|BOLD:ABX9926
Degithina|NZICH318-20|NZAC03030104|BOLD:ABX9926
Degithina|NZICH315-20|NZAC03030101|BOLD:ABX9926
Degithina|NZICH291-20|NZAC03030068|BOLD:ABX9926
Degithina|LKMSC003-15|NZAC04105966|BOLD:ABX9926
Degithina|LKMSC005-15|NZAC04106243|BOLD:ABX9926
Degithina|NZICH039-20|NZAC03030266|BOLD:ADO7052
Degithina|NZICH041-20|NZAC03030268|BOLD:ADO7052
Degithina|NZHYM1426-12|NZAC04043805|BOLD:ADO0389
Degithina|NZHYM1440-12|NZAC04043517|BOLD:ADO0389
Degithina|NZHYM1339-12|NZAC04044529|BOLD:ADO0389
Degithina|NZHYM1439-12|NZAC04044806|BOLD:ADO0389
Degithina|NZHYM1282-12|NZAC04044757|BOLD:ABX9238
Degithina|NZHYM1288-12|NZAC04044691|BOLD:ABX9238
Degithina|NZHYM1674-12|NZAC04043538|BOLD:ABX9238
Degithina|NZHYM1433-12|NZAC04044700|BOLD:ABX9238
Degithina|NZHYM1438-12|NZAC04045174|BOLD:ABX9238
Degithina|NZHYM1111-11|NZAC04044160
Degithina|NZHYM1299-12|NZAC04043637|BOLD:ABX9930
Degithina|NZHYM1341-12|NZAC04044665|BOLD:ABX9930
Degithina|NZHYM1070-11|NZAC04045252
Degithina|NZHYM1342-12|NZAC04043515|BOLD:AAV1461
Degithina|NZHYM1356-12|NZAC04043579|BOLD:AAV1461
Degithina|NZHYM029-10|NZAC04034488|BOLD:AAV1461
Degithina|NZHYM030-10|NZAC04035595|BOLD:AAV1461

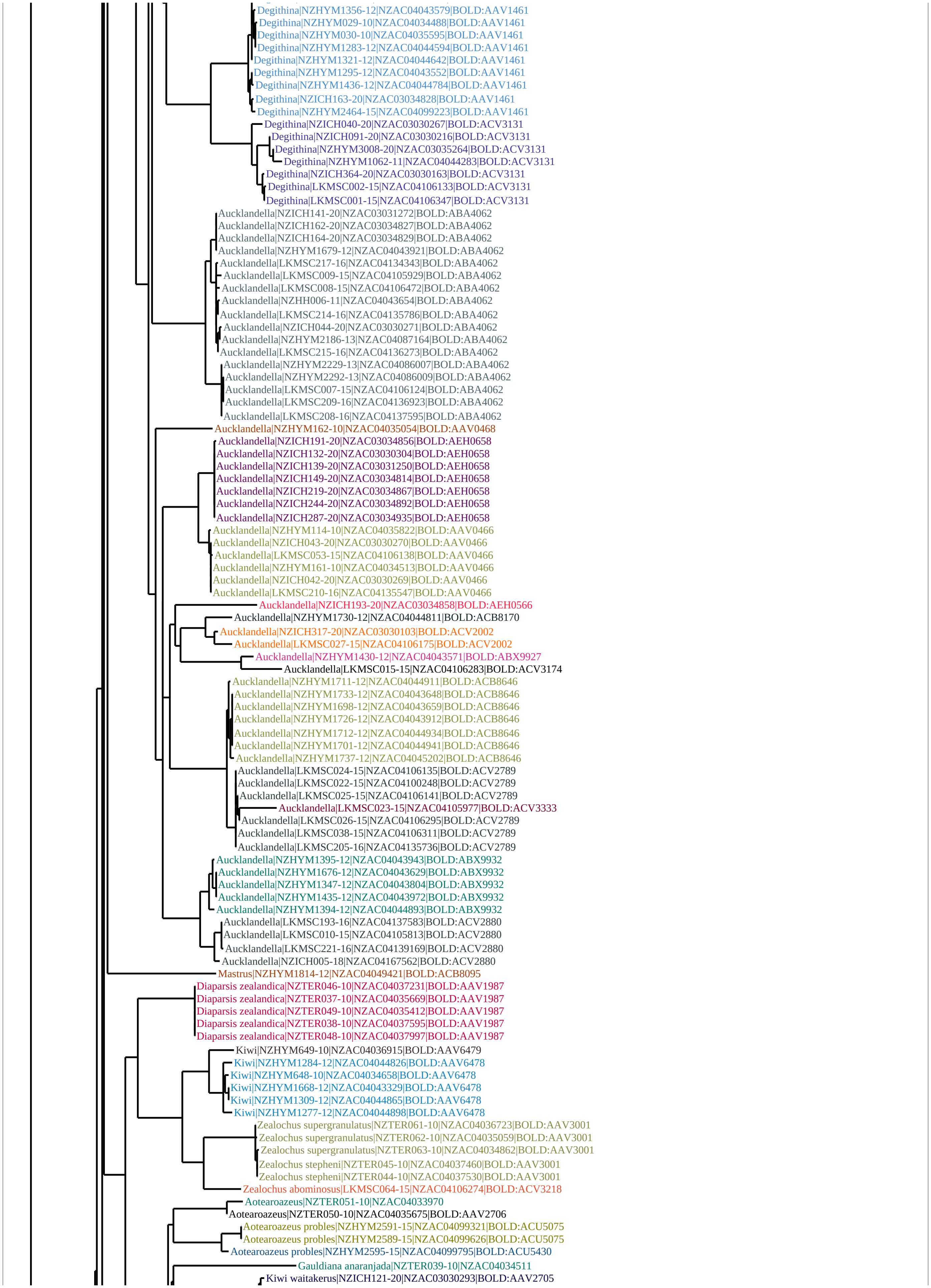
Degithina|NZHYM1356-12|NZAC04043579|BOLD:AAV1461
Degithina|NZHYM029-10|NZAC04034488|BOLD:AAV1461
Degithina|NZHYM030-10|NZAC04035595|BOLD:AAV1461
Degithina|NZHYM1283-12|NZAC04044594|BOLD:AAV1461
Degithina|NZHYM1321-12|NZAC04044642|BOLD:AAV1461
Degithina|NZHYM1295-12|NZAC04043552|BOLD:AAV1461
Degithina|NZHYM1436-12|NZAC04044784|BOLD:AAV1461
Degithina|NZICH163-20|NZAC03034828|BOLD:AAV1461
Degithina|NZHYM2464-15|NZAC04099223|BOLD:AAV1461
Degithina|NZICH040-20|NZAC03030267|BOLD:ACV3131
Degithina|NZICH091-20|NZAC03030216|BOLD:ACV3131
Degithina|NZHYM3008-20|NZAC03035264|BOLD:ACV3131
Degithina|NZHYM1062-11|NZAC04044283|BOLD:ACV3131
Degithina|NZICH364-20|NZAC03030163|BOLD:ACV3131
Degithina|LKMSC002-15|NZAC04106133|BOLD:ACV3131
Degithina|LKMSC001-15|NZAC04106347|BOLD:ACV3131
Aucklandella|NZICH141-20|NZAC03031272|BOLD:ABA4062
Aucklandella|NZICH162-20|NZAC03034827|BOLD:ABA4062
Aucklandella|NZICH164-20|NZAC03034829|BOLD:ABA4062
Aucklandella|NZHYM1679-12|NZAC04043921|BOLD:ABA4062
Aucklandella|LKMSC217-16|NZAC04134343|BOLD:ABA4062
Aucklandella|LKMSC009-15|NZAC04105929|BOLD:ABA4062
Aucklandella|LKMSC008-15|NZAC04106472|BOLD:ABA4062
Aucklandella|NZHH006-11|NZAC04043654|BOLD:ABA4062
Aucklandella|LKMSC214-16|NZAC04135786|BOLD:ABA4062
Aucklandella|NZICH044-20|NZAC03030271|BOLD:ABA4062
Aucklandella|NZHYM2186-13|NZAC04087164|BOLD:ABA4062
Aucklandella|LKMSC215-16|NZAC04136273|BOLD:ABA4062
Aucklandella|NZHYM2229-13|NZAC04086007|BOLD:ABA4062
Aucklandella|NZHYM2292-13|NZAC04086009|BOLD:ABA4062
Aucklandella|LKMSC007-15|NZAC04106124|BOLD:ABA4062
Aucklandella|LKMSC209-16|NZAC04136923|BOLD:ABA4062
Aucklandella|LKMSC208-16|NZAC04137595|BOLD:ABA4062
Aucklandella|NZHYM162-10|NZAC04035054|BOLD:AAV0468
Aucklandella|NZICH191-20|NZAC03034856|BOLD:AEH0658
Aucklandella|NZICH132-20|NZAC03030304|BOLD:AEH0658
Aucklandella|NZICH139-20|NZAC03031250|BOLD:AEH0658
Aucklandella|NZICH149-20|NZAC03034814|BOLD:AEH0658
Aucklandella|NZICH219-20|NZAC03034867|BOLD:AEH0658
Aucklandella|NZICH244-20|NZAC03034892|BOLD:AEH0658
Aucklandella|NZICH287-20|NZAC03034935|BOLD:AEH0658
Aucklandella|NZHYM114-10|NZAC04035822|BOLD:AAV0466
Aucklandella|NZICH043-20|NZAC03030270|BOLD:AAV0466
Aucklandella|LKMSC053-15|NZAC04106138|BOLD:AAV0466
Aucklandella|NZHYM161-10|NZAC04034513|BOLD:AAV0466
Aucklandella|NZICH042-20|NZAC03030269|BOLD:AAV0466
Aucklandella|LKMSC210-16|NZAC04135547|BOLD:AAV0466
Aucklandella|NZICH193-20|NZAC03034858|BOLD:AEH0566
Aucklandella|NZHYM1730-12|NZAC04044811|BOLD:ACB8170
Aucklandella|NZICH317-20|NZAC03030103|BOLD:ACV2002
Aucklandella|LKMSC027-15|NZAC04106175|BOLD:ACV2002
Aucklandella|NZHYM1430-12|NZAC04043571|BOLD:ABX9927
Aucklandella|LKMSC015-15|NZAC04106283|BOLD:ACV3174
Aucklandella|NZHYM1711-12|NZAC04044911|BOLD:ACB8646
Aucklandella|NZHYM1733-12|NZAC04043648|BOLD:ACB8646
Aucklandella|NZHYM1698-12|NZAC04043659|BOLD:ACB8646
Aucklandella|NZHYM1726-12|NZAC04043912|BOLD:ACB8646
Aucklandella|NZHYM1712-12|NZAC04044934|BOLD:ACB8646
Aucklandella|NZHYM1701-12|NZAC04044941|BOLD:ACB8646
Aucklandella|NZHYM1737-12|NZAC04045202|BOLD:ACB8646
Aucklandella|LKMSC024-15|NZAC04106135|BOLD:ACV2789
Aucklandella|LKMSC022-15|NZAC04100248|BOLD:ACV2789
Aucklandella|LKMSC025-15|NZAC04106141|BOLD:ACV2789
Aucklandella|LKMSC023-15|NZAC04105977|BOLD:ACV3333
Aucklandella|LKMSC026-15|NZAC04106295|BOLD:ACV2789
Aucklandella|LKMSC038-15|NZAC04106311|BOLD:ACV2789
Aucklandella|LKMSC205-16|NZAC04135736|BOLD:ACV2789
Aucklandella|NZHYM1395-12|NZAC04043943|BOLD:ABX9932
Aucklandella|NZHYM1676-12|NZAC04043629|BOLD:ABX9932
Aucklandella|NZHYM1347-12|NZAC04043804|BOLD:ABX9932
Aucklandella|NZHYM1435-12|NZAC04043972|BOLD:ABX9932
Aucklandella|NZHYM1394-12|NZAC04044893|BOLD:ABX9932
Aucklandella|LKMSC193-16|NZAC04137583|BOLD:ACV2880
Aucklandella|LKMSC010-15|NZAC04105813|BOLD:ACV2880
Aucklandella|LKMSC221-16|NZAC04139169|BOLD:ACV2880
Aucklandella|NZICH005-18|NZAC04167562|BOLD:ACV2880
Mastrus|NZHYM1814-12|NZAC04049421|BOLD:ACB8095
Diaparsis zealandica|NZTER046-10|NZAC04037231|BOLD:AAV1987
Diaparsis zealandica|NZTER037-10|NZAC04035669|BOLD:AAV1987
Diaparsis zealandica|NZTER049-10|NZAC04035412|BOLD:AAV1987
Diaparsis zealandica|NZTER038-10|NZAC04037595|BOLD:AAV1987
Diaparsis zealandica|NZTER048-10|NZAC04037997|BOLD:AAV1987
Kiwi|NZHYM649-10|NZAC04036915|BOLD:AAV6479
Kiwi|NZHYM1284-12|NZAC04044826|BOLD:AAV6478
Kiwi|NZHYM648-10|NZAC04034658|BOLD:AAV6478
Kiwi|NZHYM1668-12|NZAC04043329|BOLD:AAV6478
Kiwi|NZHYM1309-12|NZAC04044865|BOLD:AAV6478
Kiwi|NZHYM1277-12|NZAC04044898|BOLD:AAV6478
Zealochus supergranulatus|NZTER061-10|NZAC04036723|BOLD:AAV3001
Zealochus supergranulatus|NZTER062-10|NZAC04035059|BOLD:AAV3001
Zealochus supergranulatus|NZTER063-10|NZAC04034862|BOLD:AAV3001
Zealochus stepheni|NZTER045-10|NZAC04037460|BOLD:AAV3001
Zealochus stepheni|NZTER044-10|NZAC04037530|BOLD:AAV3001
Zealochus abominosus|LKMSC064-15|NZAC04106274|BOLD:ACV3218
Aotearoazeus|NZTER051-10|NZAC04033970
Aotearoazeus|NZTER050-10|NZAC04035675|BOLD:AAV2706
Aotearoazeus probles|NZHYM2591-15|NZAC04099321|BOLD:ACU5075
Aotearoazeus probles|NZHYM2589-15|NZAC04099626|BOLD:ACU5075
Aotearoazeus probles|NZHYM2595-15|NZAC04099795|BOLD:ACU5430
Gauldiana anaranjada|NZTER039-10|NZAC04034511
Kiwi waitakerus|NZICH121-20|NZAC03030293|BOLD:AAV2705

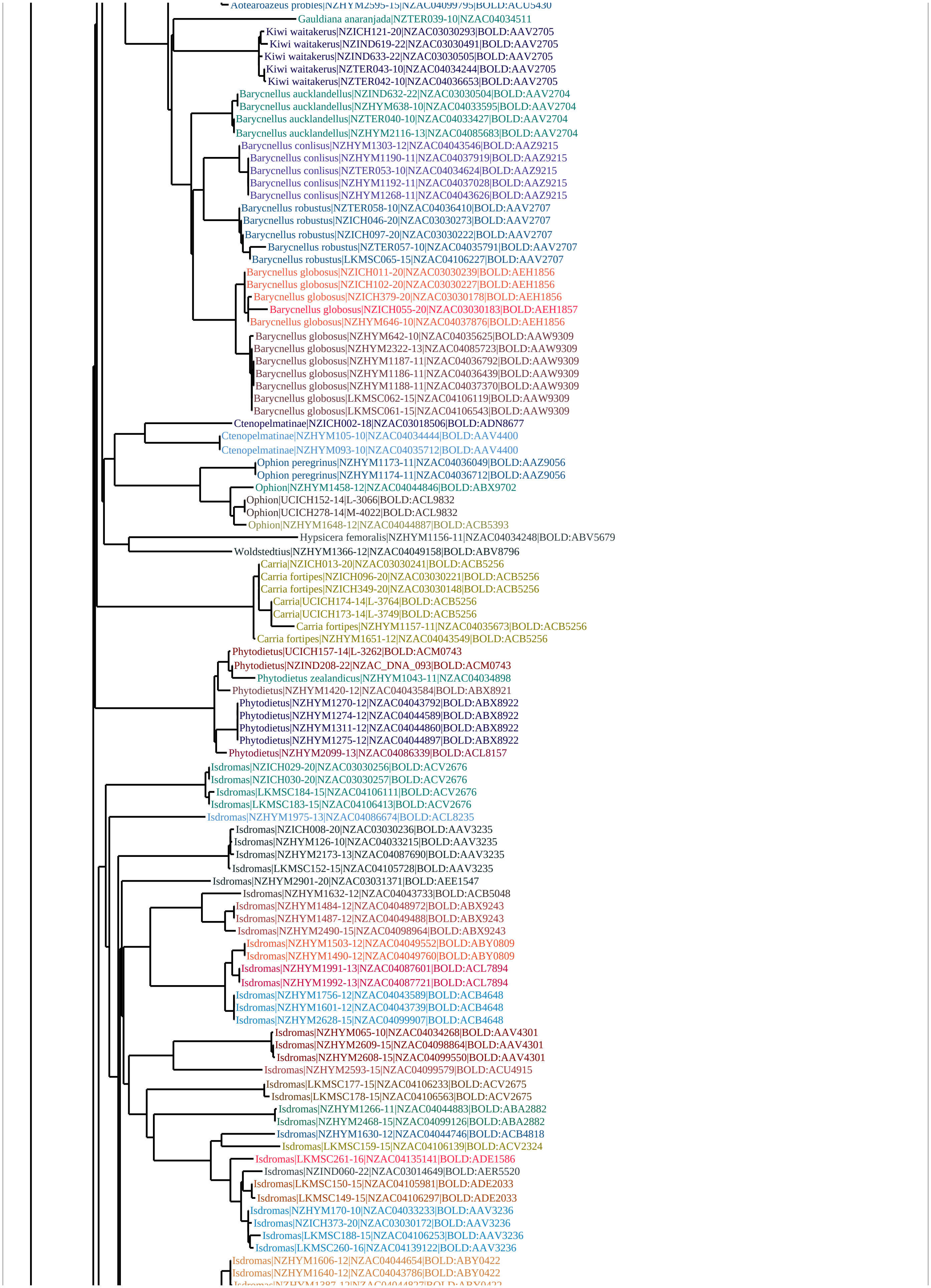
Aotearoazeus probles|NZHYM2595-15|NZAC04099795|BOLD:ACU5430
Gauldiana anaranjada|NZTER039-10|NZAC04034511
Kiwi waitakerus|NZICH121-20|NZAC03030293|BOLD:AAV2705
Kiwi waitakerus|NZIND619-22|NZAC03030491|BOLD:AAV2705
Kiwi waitakerus|NZIND633-22|NZAC03030505|BOLD:AAV2705
Kiwi waitakerus|NZTER043-10|NZAC04034244|BOLD:AAV2705
Kiwi waitakerus|NZTER042-10|NZAC04036653|BOLD:AAV2705
Barycnellus aucklandellus|NZIND632-22|NZAC03030504|BOLD:AAV2704
Barycnellus aucklandellus|NZHYM638-10|NZAC04033595|BOLD:AAV2704
Barycnellus aucklandellus|NZTER040-10|NZAC04033427|BOLD:AAV2704
Barycnellus aucklandellus|NZHYM2116-13|NZAC04085683|BOLD:AAV2704
Barycnellus conlisus|NZHYM1303-12|NZAC04043546|BOLD:AAZ9215
Barycnellus conlisus|NZHYM1190-11|NZAC04037919|BOLD:AAZ9215
Barycnellus conlisus|NZTER053-10|NZAC04034624|BOLD:AAZ9215
Barycnellus conlisus|NZHYM1192-11|NZAC04037028|BOLD:AAZ9215
Barycnellus conlisus|NZHYM1268-11|NZAC04043626|BOLD:AAZ9215
Barycnellus robustus|NZTER058-10|NZAC04036410|BOLD:AAV2707
Barycnellus robustus|NZICH046-20|NZAC03030273|BOLD:AAV2707
Barycnellus robustus|NZICH097-20|NZAC03030222|BOLD:AAV2707
Barycnellus robustus|NZTER057-10|NZAC04035791|BOLD:AAV2707
Barycnellus robustus|LKMSC065-15|NZAC04106227|BOLD:AAV2707
Barycnellus globosus|NZICH011-20|NZAC03030239|BOLD:AEH1856
Barycnellus globosus|NZICH102-20|NZAC03030227|BOLD:AEH1856
Barycnellus globosus|NZICH379-20|NZAC03030178|BOLD:AEH1856
Barycnellus globosus|NZICH055-20|NZAC03030183|BOLD:AEH1857
Barycnellus globosus|NZHYM646-10|NZAC04037876|BOLD:AEH1856
Barycnellus globosus|NZHYM642-10|NZAC04035625|BOLD:AAW9309
Barycnellus globosus|NZHYM2322-13|NZAC04085723|BOLD:AAW9309
Barycnellus globosus|NZHYM1187-11|NZAC04036792|BOLD:AAW9309
Barycnellus globosus|NZHYM1186-11|NZAC04036439|BOLD:AAW9309
Barycnellus globosus|NZHYM1188-11|NZAC04037370|BOLD:AAW9309
Barycnellus globosus|LKMSC062-15|NZAC04106119|BOLD:AAW9309
Barycnellus globosus|LKMSC061-15|NZAC04106543|BOLD:AAW9309
Ctenopelmatinae|NZICH002-18|NZAC03018506|BOLD:ADN8677
Ctenopelmatinae|NZHYM105-10|NZAC04034444|BOLD:AAV4400
Ctenopelmatinae|NZHYM093-10|NZAC04035712|BOLD:AAV4400
Ophion peregrinus|NZHYM1173-11|NZAC04036049|BOLD:AAZ9056
Ophion peregrinus|NZHYM1174-11|NZAC04036712|BOLD:AAZ9056
Ophion|NZHYM1458-12|NZAC04044846|BOLD:ABX9702
Ophion|UCICH152-14|L-3066|BOLD:ACL9832
Ophion|UCICH278-14|M-4022|BOLD:ACL9832
Ophion|NZHYM1648-12|NZAC04044887|BOLD:ACB5393
Hypsicera femoralis|NZHYM1156-11|NZAC04034248|BOLD:ABV5679
Woldstedtius|NZHYM1366-12|NZAC04049158|BOLD:ABV8796
Carria|NZICH013-20|NZAC03030241|BOLD:ACB5256
Carria fortipes|NZICH096-20|NZAC03030221|BOLD:ACB5256
Carria fortipes|NZICH349-20|NZAC03030148|BOLD:ACB5256
Carria|UCICH174-14|L-3764|BOLD:ACB5256
Carria|UCICH173-14|L-3749|BOLD:ACB5256
Carria fortipes|NZHYM1157-11|NZAC04035673|BOLD:ACB5256
Carria fortipes|NZHYM1651-12|NZAC04043549|BOLD:ACB5256
Phytodietus|UCICH157-14|L-3262|BOLD:ACM0743
Phytodietus|NZIND208-22|NZAC_DNA_093|BOLD:ACM0743
Phytodietus zealandicus|NZHYM1043-11|NZAC04034898
Phytodietus|NZHYM1420-12|NZAC04043584|BOLD:ABX8921
Phytodietus|NZHYM1270-12|NZAC04043792|BOLD:ABX8922
Phytodietus|NZHYM1274-12|NZAC04044589|BOLD:ABX8922
Phytodietus|NZHYM1311-12|NZAC04044860|BOLD:ABX8922
Phytodietus|NZHYM1275-12|NZAC04044897|BOLD:ABX8922
Phytodietus|NZHYM2099-13|NZAC04086339|BOLD:ACL8157
Isdromas|NZICH029-20|NZAC03030256|BOLD:ACV2676
Isdromas|NZICH030-20|NZAC03030257|BOLD:ACV2676
Isdromas|LKMSC184-15|NZAC04106111|BOLD:ACV2676
Isdromas|LKMSC183-15|NZAC04106413|BOLD:ACV2676
Isdromas|NZHYM1975-13|NZAC04086674|BOLD:ACL8235
Isdromas|NZICH008-20|NZAC03030236|BOLD:AAV3235
Isdromas|NZHYM126-10|NZAC04033215|BOLD:AAV3235
Isdromas|NZHYM2173-13|NZAC04087690|BOLD:AAV3235
Isdromas|LKMSC152-15|NZAC04105728|BOLD:AAV3235
Isdromas|NZHYM2901-20|NZAC03031371|BOLD:AEE1547
Isdromas|NZHYM1632-12|NZAC04043733|BOLD:ACB5048
Isdromas|NZHYM1484-12|NZAC04048972|BOLD:ABX9243
Isdromas|NZHYM1487-12|NZAC04049488|BOLD:ABX9243
Isdromas|NZHYM2490-15|NZAC04098964|BOLD:ABX9243
Isdromas|NZHYM1503-12|NZAC04049552|BOLD:ABY0809
Isdromas|NZHYM1490-12|NZAC04049760|BOLD:ABY0809
Isdromas|NZHYM1991-13|NZAC04087601|BOLD:ACL7894
Isdromas|NZHYM1992-13|NZAC04087721|BOLD:ACL7894
Isdromas|NZHYM1756-12|NZAC04043589|BOLD:ACB4648
Isdromas|NZHYM1601-12|NZAC04043739|BOLD:ACB4648
Isdromas|NZHYM2628-15|NZAC04099907|BOLD:ACB4648
Isdromas|NZHYM065-10|NZAC04034268|BOLD:AAV4301
Isdromas|NZHYM2609-15|NZAC04098864|BOLD:AAV4301
Isdromas|NZHYM2608-15|NZAC04099550|BOLD:AAV4301
Isdromas|NZHYM2593-15|NZAC04099579|BOLD:ACU4915
Isdromas|LKMSC177-15|NZAC04106233|BOLD:ACV2675
Isdromas|LKMSC178-15|NZAC04106563|BOLD:ACV2675
Isdromas|NZHYM1266-11|NZAC04044883|BOLD:ABA2882
Isdromas|NZHYM2468-15|NZAC04099126|BOLD:ABA2882
Isdromas|NZHYM1630-12|NZAC04044746|BOLD:ACB4818
Isdromas|LKMSC159-15|NZAC04106139|BOLD:ACV2324
Isdromas|LKMSC261-16|NZAC04135141|BOLD:ADE1586
Isdromas|NZIND060-22|NZAC03014649|BOLD:AER5520
Isdromas|LKMSC150-15|NZAC04105981|BOLD:ADE2033
Isdromas|LKMSC149-15|NZAC04106297|BOLD:ADE2033
Isdromas|NZHYM170-10|NZAC04033233|BOLD:AAV3236
Isdromas|NZICH373-20|NZAC03030172|BOLD:AAV3236
Isdromas|LKMSC188-15|NZAC04106253|BOLD:AAV3236
Isdromas|LKMSC260-16|NZAC04139122|BOLD:AAV3236
Isdromas|NZHYM1606-12|NZAC04044654|BOLD:ABY0422
Isdromas|NZHYM1640-12|NZAC04043786|BOLD:ABY0422
Isdromas|NZHYM1387-12|NZAC04044827|BOLD:ABY0422

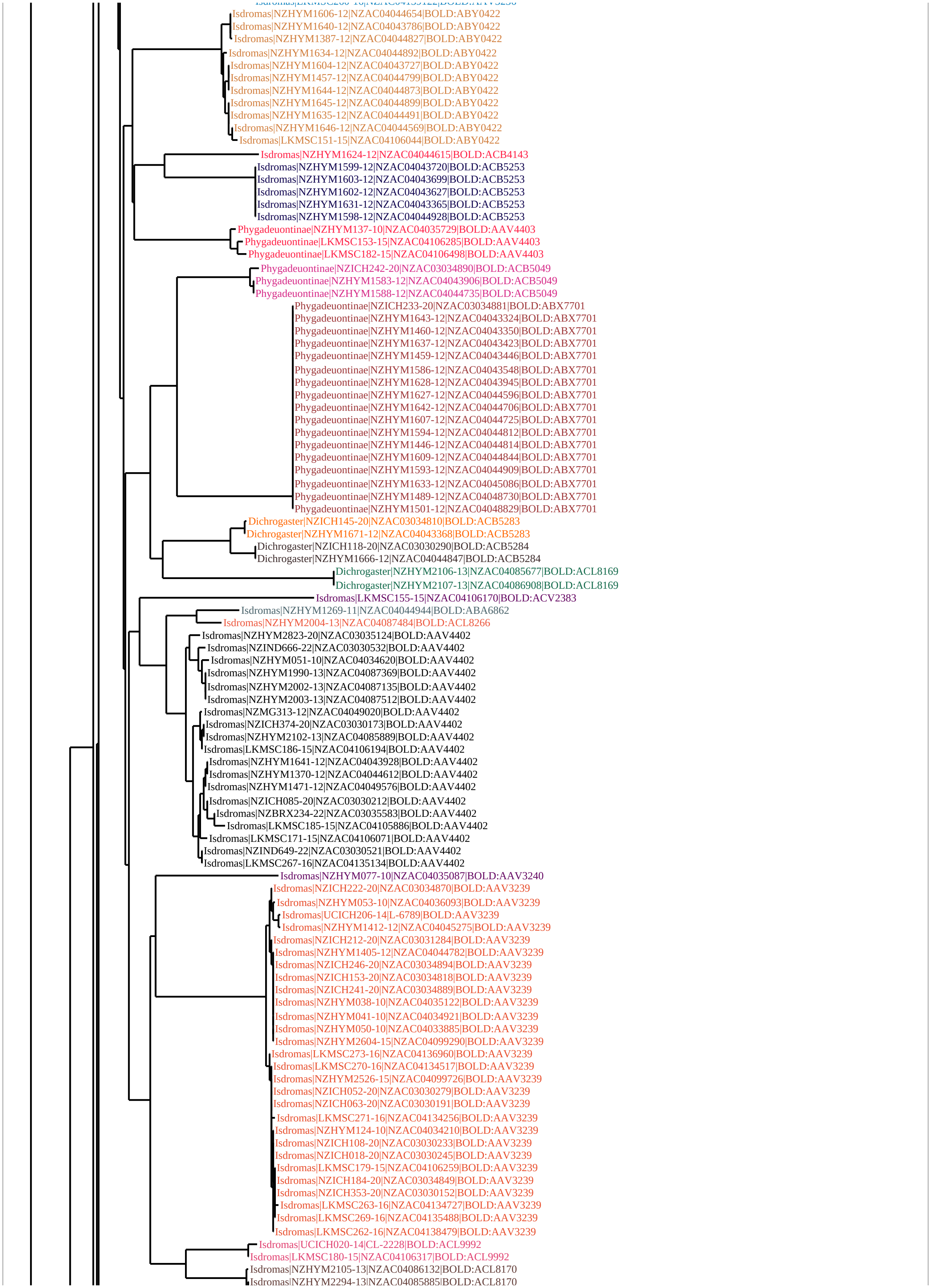
Isdromas|NZHYM1606-12|NZAC04044654|BOLD:ABY0422
Isdromas|NZHYM1640-12|NZAC04043786|BOLD:ABY0422
Isdromas|NZHYM1387-12|NZAC04044827|BOLD:ABY0422
Isdromas|NZHYM1634-12|NZAC04044892|BOLD:ABY0422
Isdromas|NZHYM1604-12|NZAC04043727|BOLD:ABY0422
Isdromas|NZHYM1457-12|NZAC04044799|BOLD:ABY0422
Isdromas|NZHYM1644-12|NZAC04044873|BOLD:ABY0422
Isdromas|NZHYM1645-12|NZAC04044899|BOLD:ABY0422
Isdromas|NZHYM1635-12|NZAC04044491|BOLD:ABY0422
Isdromas|NZHYM1646-12|NZAC04044569|BOLD:ABY0422
Isdromas|LKMSC151-15|NZAC04106044|BOLD:ABY0422
Isdromas|NZHYM1624-12|NZAC04044615|BOLD:ACB4143
Isdromas|NZHYM1599-12|NZAC04043720|BOLD:ACB5253
Isdromas|NZHYM1603-12|NZAC04043699|BOLD:ACB5253
Isdromas|NZHYM1602-12|NZAC04043627|BOLD:ACB5253
Isdromas|NZHYM1631-12|NZAC04043365|BOLD:ACB5253
Isdromas|NZHYM1598-12|NZAC04044928|BOLD:ACB5253
Phygadeuontinae|NZHYM137-10|NZAC04035729|BOLD:AAV4403
Phygadeuontinae|LKMSC153-15|NZAC04106285|BOLD:AAV4403
Phygadeuontinae|LKMSC182-15|NZAC04106498|BOLD:AAV4403
Phygadeuontinae|NZICH242-20|NZAC03034890|BOLD:ACB5049
Phygadeuontinae|NZHYM1583-12|NZAC04043906|BOLD:ACB5049
Phygadeuontinae|NZHYM1588-12|NZAC04044735|BOLD:ACB5049
Phygadeuontinae|NZICH233-20|NZAC03034881|BOLD:ABX7701
Phygadeuontinae|NZHYM1643-12|NZAC04043324|BOLD:ABX7701
Phygadeuontinae|NZHYM1460-12|NZAC04043350|BOLD:ABX7701
Phygadeuontinae|NZHYM1637-12|NZAC04043423|BOLD:ABX7701
Phygadeuontinae|NZHYM1459-12|NZAC04043446|BOLD:ABX7701
Phygadeuontinae|NZHYM1586-12|NZAC04043548|BOLD:ABX7701
Phygadeuontinae|NZHYM1628-12|NZAC04043945|BOLD:ABX7701
Phygadeuontinae|NZHYM1627-12|NZAC04044596|BOLD:ABX7701
Phygadeuontinae|NZHYM1642-12|NZAC04044706|BOLD:ABX7701
Phygadeuontinae|NZHYM1607-12|NZAC04044725|BOLD:ABX7701
Phygadeuontinae|NZHYM1594-12|NZAC04044812|BOLD:ABX7701
Phygadeuontinae|NZHYM1446-12|NZAC04044814|BOLD:ABX7701
Phygadeuontinae|NZHYM1609-12|NZAC04044844|BOLD:ABX7701
Phygadeuontinae|NZHYM1593-12|NZAC04044909|BOLD:ABX7701
Phygadeuontinae|NZHYM1633-12|NZAC04045086|BOLD:ABX7701
Phygadeuontinae|NZHYM1489-12|NZAC04048730|BOLD:ABX7701
Phygadeuontinae|NZHYM1501-12|NZAC04048829|BOLD:ABX7701
Dichrogaster|NZICH145-20|NZAC03034810|BOLD:ACB5283
Dichrogaster|NZHYM1671-12|NZAC04043368|BOLD:ACB5283
Dichrogaster|NZICH118-20|NZAC03030290|BOLD:ACB5284
Dichrogaster|NZHYM1666-12|NZAC04044847|BOLD:ACB5284
Dichrogaster|NZHYM2106-13|NZAC04085677|BOLD:ACL8169
Dichrogaster|NZHYM2107-13|NZAC04086908|BOLD:ACL8169
Isdromas|LKMSC155-15|NZAC04106170|BOLD:ACV2383
Isdromas|NZHYM1269-11|NZAC04044944|BOLD:ABA6862
Isdromas|NZHYM2004-13|NZAC04087484|BOLD:ACL8266
Isdromas|NZHYM2823-20|NZAC03035124|BOLD:AAV4402
Isdromas|NZIND666-22|NZAC03030532|BOLD:AAV4402
Isdromas|NZHYM051-10|NZAC04034620|BOLD:AAV4402
Isdromas|NZHYM1990-13|NZAC04087369|BOLD:AAV4402
Isdromas|NZHYM2002-13|NZAC04087135|BOLD:AAV4402
Isdromas|NZHYM2003-13|NZAC04087512|BOLD:AAV4402
Isdromas|NZMG313-12|NZAC04049020|BOLD:AAV4402
Isdromas|NZICH374-20|NZAC03030173|BOLD:AAV4402
Isdromas|NZHYM2102-13|NZAC04085889|BOLD:AAV4402
Isdromas|LKMSC186-15|NZAC04106194|BOLD:AAV4402
Isdromas|NZHYM1641-12|NZAC04043928|BOLD:AAV4402
Isdromas|NZHYM1370-12|NZAC04044612|BOLD:AAV4402
Isdromas|NZHYM1471-12|NZAC04049576|BOLD:AAV4402
Isdromas|NZICH085-20|NZAC03030212|BOLD:AAV4402
Isdromas|NZBRX234-22|NZAC03035583|BOLD:AAV4402
Isdromas|LKMSC185-15|NZAC04105886|BOLD:AAV4402
Isdromas|LKMSC171-15|NZAC04106071|BOLD:AAV4402
Isdromas|NZIND649-22|NZAC03030521|BOLD:AAV4402
Isdromas|LKMSC267-16|NZAC04135134|BOLD:AAV4402
Isdromas|NZHYM077-10|NZAC04035087|BOLD:AAV3240
Isdromas|NZICH222-20|NZAC03034870|BOLD:AAV3239
Isdromas|NZHYM053-10|NZAC04036093|BOLD:AAV3239
Isdromas|UCICH206-14|L-6789|BOLD:AAV3239
Isdromas|NZHYM1412-12|NZAC04045275|BOLD:AAV3239
Isdromas|NZICH212-20|NZAC03031284|BOLD:AAV3239
Isdromas|NZHYM1405-12|NZAC04044782|BOLD:AAV3239
Isdromas|NZICH246-20|NZAC03034894|BOLD:AAV3239
Isdromas|NZICH153-20|NZAC03034818|BOLD:AAV3239
Isdromas|NZICH241-20|NZAC03034889|BOLD:AAV3239
Isdromas|NZHYM038-10|NZAC04035122|BOLD:AAV3239
Isdromas|NZHYM041-10|NZAC04034921|BOLD:AAV3239
Isdromas|NZHYM050-10|NZAC04033885|BOLD:AAV3239
Isdromas|NZHYM2604-15|NZAC04099290|BOLD:AAV3239
Isdromas|LKMSC273-16|NZAC04136960|BOLD:AAV3239
Isdromas|LKMSC270-16|NZAC04134517|BOLD:AAV3239
Isdromas|NZHYM2526-15|NZAC04099726|BOLD:AAV3239
Isdromas|NZICH052-20|NZAC03030279|BOLD:AAV3239
Isdromas|NZICH063-20|NZAC03030191|BOLD:AAV3239
Isdromas|LKMSC271-16|NZAC04134256|BOLD:AAV3239
Isdromas|NZHYM124-10|NZAC04034210|BOLD:AAV3239
Isdromas|NZICH108-20|NZAC03030233|BOLD:AAV3239
Isdromas|NZICH018-20|NZAC03030245|BOLD:AAV3239
Isdromas|LKMSC179-15|NZAC04106259|BOLD:AAV3239
Isdromas|NZICH184-20|NZAC03034849|BOLD:AAV3239
Isdromas|NZICH353-20|NZAC03030152|BOLD:AAV3239
Isdromas|LKMSC263-16|NZAC04134727|BOLD:AAV3239
Isdromas|LKMSC269-16|NZAC04135488|BOLD:AAV3239
Isdromas|LKMSC262-16|NZAC04138479|BOLD:AAV3239
Isdromas|UCICH020-14|CL-2228|BOLD:ACL9992
Isdromas|LKMSC180-15|NZAC04106317|BOLD:ACL9992
Isdromas|NZHYM2105-13|NZAC04086132|BOLD:ACL8170
Isdromas|NZHYM2294-13|NZAC04085885|BOLD:ACL8170

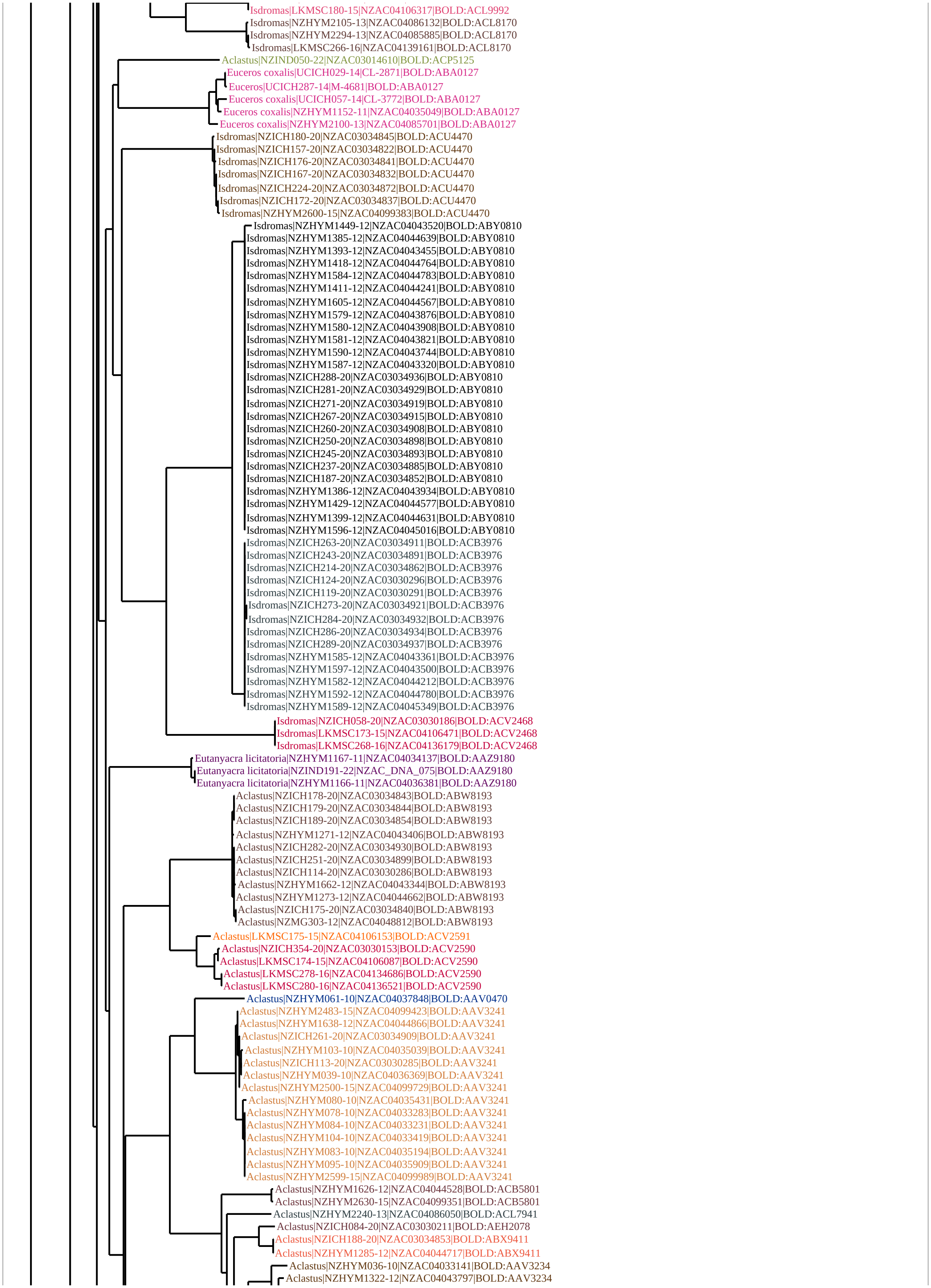
Isdromas|LKMSC180-15|NZAC04106317|BOLD:ACL9992
Isdromas|NZHYM2105-13|NZAC04086132|BOLD:ACL8170
Isdromas|NZHYM2294-13|NZAC04085885|BOLD:ACL8170
Isdromas|LKMSC266-16|NZAC04139161|BOLD:ACL8170
Aclastus|NZIND050-22|NZAC03014610|BOLD:ACP5125
Euceros coxalis|UCICH029-14|CL-2871|BOLD:ABA0127
Euceros|UCICH287-14|M-4681|BOLD:ABA0127
Euceros coxalis|UCICH057-14|CL-3772|BOLD:ABA0127
Euceros coxalis|NZHYM1152-11|NZAC04035049|BOLD:ABA0127
Euceros coxalis|NZHYM2100-13|NZAC04085701|BOLD:ABA0127
Isdromas|NZICH180-20|NZAC03034845|BOLD:ACU4470
Isdromas|NZICH157-20|NZAC03034822|BOLD:ACU4470
Isdromas|NZICH176-20|NZAC03034841|BOLD:ACU4470
Isdromas|NZICH167-20|NZAC03034832|BOLD:ACU4470
Isdromas|NZICH224-20|NZAC03034872|BOLD:ACU4470
Isdromas|NZICH172-20|NZAC03034837|BOLD:ACU4470
Isdromas|NZHYM2600-15|NZAC04099383|BOLD:ACU4470
Isdromas|NZHYM1449-12|NZAC04043520|BOLD:ABY0810
Isdromas|NZHYM1385-12|NZAC04044639|BOLD:ABY0810
Isdromas|NZHYM1393-12|NZAC04043455|BOLD:ABY0810
Isdromas|NZHYM1418-12|NZAC04044764|BOLD:ABY0810
Isdromas|NZHYM1584-12|NZAC04044783|BOLD:ABY0810
Isdromas|NZHYM1411-12|NZAC04044241|BOLD:ABY0810
Isdromas|NZHYM1605-12|NZAC04044567|BOLD:ABY0810
Isdromas|NZHYM1579-12|NZAC04043876|BOLD:ABY0810
Isdromas|NZHYM1580-12|NZAC04043908|BOLD:ABY0810
Isdromas|NZHYM1581-12|NZAC04043821|BOLD:ABY0810
Isdromas|NZHYM1590-12|NZAC04043744|BOLD:ABY0810
Isdromas|NZHYM1587-12|NZAC04043320|BOLD:ABY0810
Isdromas|NZICH288-20|NZAC03034936|BOLD:ABY0810
Isdromas|NZICH281-20|NZAC03034929|BOLD:ABY0810
Isdromas|NZICH271-20|NZAC03034919|BOLD:ABY0810
Isdromas|NZICH267-20|NZAC03034915|BOLD:ABY0810
Isdromas|NZICH260-20|NZAC03034908|BOLD:ABY0810
Isdromas|NZICH250-20|NZAC03034898|BOLD:ABY0810
Isdromas|NZICH245-20|NZAC03034893|BOLD:ABY0810
Isdromas|NZICH237-20|NZAC03034885|BOLD:ABY0810
Isdromas|NZICH187-20|NZAC03034852|BOLD:ABY0810
Isdromas|NZHYM1386-12|NZAC04043934|BOLD:ABY0810
Isdromas|NZHYM1429-12|NZAC04044577|BOLD:ABY0810
Isdromas|NZHYM1399-12|NZAC04044631|BOLD:ABY0810
Isdromas|NZHYM1596-12|NZAC04045016|BOLD:ABY0810
Isdromas|NZICH263-20|NZAC03034911|BOLD:ACB3976
Isdromas|NZICH243-20|NZAC03034891|BOLD:ACB3976
Isdromas|NZICH214-20|NZAC03034862|BOLD:ACB3976
Isdromas|NZICH124-20|NZAC03030296|BOLD:ACB3976
Isdromas|NZICH119-20|NZAC03030291|BOLD:ACB3976
Isdromas|NZICH273-20|NZAC03034921|BOLD:ACB3976
Isdromas|NZICH284-20|NZAC03034932|BOLD:ACB3976
Isdromas|NZICH286-20|NZAC03034934|BOLD:ACB3976
Isdromas|NZICH289-20|NZAC03034937|BOLD:ACB3976
Isdromas|NZHYM1585-12|NZAC04043361|BOLD:ACB3976
Isdromas|NZHYM1597-12|NZAC04043500|BOLD:ACB3976
Isdromas|NZHYM1582-12|NZAC04044212|BOLD:ACB3976
Isdromas|NZHYM1592-12|NZAC04044780|BOLD:ACB3976
Isdromas|NZHYM1589-12|NZAC04045349|BOLD:ACB3976
Isdromas|NZICH058-20|NZAC03030186|BOLD:ACV2468
Isdromas|LKMSC173-15|NZAC04106471|BOLD:ACV2468
Isdromas|LKMSC268-16|NZAC04136179|BOLD:ACV2468
Eutanyacra licitatoria|NZHYM1167-11|NZAC04034137|BOLD:AAZ9180
Eutanyacra licitatoria|NZIND191-22|NZAC_DNA_075|BOLD:AAZ9180
Eutanyacra licitatoria|NZHYM1166-11|NZAC04036381|BOLD:AAZ9180
Aclastus|NZICH178-20|NZAC03034843|BOLD:ABW8193
Aclastus|NZICH179-20|NZAC03034844|BOLD:ABW8193
Aclastus|NZICH189-20|NZAC03034854|BOLD:ABW8193
Aclastus|NZHYM1271-12|NZAC04043406|BOLD:ABW8193
Aclastus|NZICH282-20|NZAC03034930|BOLD:ABW8193
Aclastus|NZICH251-20|NZAC03034899|BOLD:ABW8193
Aclastus|NZICH114-20|NZAC03030286|BOLD:ABW8193
Aclastus|NZHYM1662-12|NZAC04043344|BOLD:ABW8193
Aclastus|NZHYM1273-12|NZAC04044662|BOLD:ABW8193
Aclastus|NZICH175-20|NZAC03034840|BOLD:ABW8193
Aclastus|NZMG303-12|NZAC04048812|BOLD:ABW8193
Aclastus|LKMSC175-15|NZAC04106153|BOLD:ACV2591
Aclastus|NZICH354-20|NZAC03030153|BOLD:ACV2590
Aclastus|LKMSC174-15|NZAC04106087|BOLD:ACV2590
Aclastus|LKMSC278-16|NZAC04134686|BOLD:ACV2590
Aclastus|LKMSC280-16|NZAC04136521|BOLD:ACV2590
Aclastus|NZHYM061-10|NZAC04037848|BOLD:AAV0470
Aclastus|NZHYM2483-15|NZAC04099423|BOLD:AAV3241
Aclastus|NZHYM1638-12|NZAC04044866|BOLD:AAV3241
Aclastus|NZICH261-20|NZAC03034909|BOLD:AAV3241
Aclastus|NZHYM103-10|NZAC04035039|BOLD:AAV3241
Aclastus|NZICH113-20|NZAC03030285|BOLD:AAV3241
Aclastus|NZHYM039-10|NZAC04036369|BOLD:AAV3241
Aclastus|NZHYM2500-15|NZAC04099729|BOLD:AAV3241
Aclastus|NZHYM080-10|NZAC04035431|BOLD:AAV3241
Aclastus|NZHYM078-10|NZAC04033283|BOLD:AAV3241
Aclastus|NZHYM084-10|NZAC04033231|BOLD:AAV3241
Aclastus|NZHYM104-10|NZAC04033419|BOLD:AAV3241
Aclastus|NZHYM083-10|NZAC04035194|BOLD:AAV3241
Aclastus|NZHYM095-10|NZAC04035909|BOLD:AAV3241
Aclastus|NZHYM2599-15|NZAC04099989|BOLD:AAV3241
Aclastus|NZHYM1626-12|NZAC04044528|BOLD:ACB5801
Aclastus|NZHYM2630-15|NZAC04099351|BOLD:ACB5801
Aclastus|NZHYM2240-13|NZAC04086050|BOLD:ACL7941
Aclastus|NZICH084-20|NZAC03030211|BOLD:AEH2078
Aclastus|NZICH188-20|NZAC03034853|BOLD:ABX9411
Aclastus|NZHYM1285-12|NZAC04044717|BOLD:ABX9411
Aclastus|NZHYM036-10|NZAC04033141|BOLD:AAV3234
Aclastus|NZHYM1322-12|NZAC04043797|BOLD:AAV3234

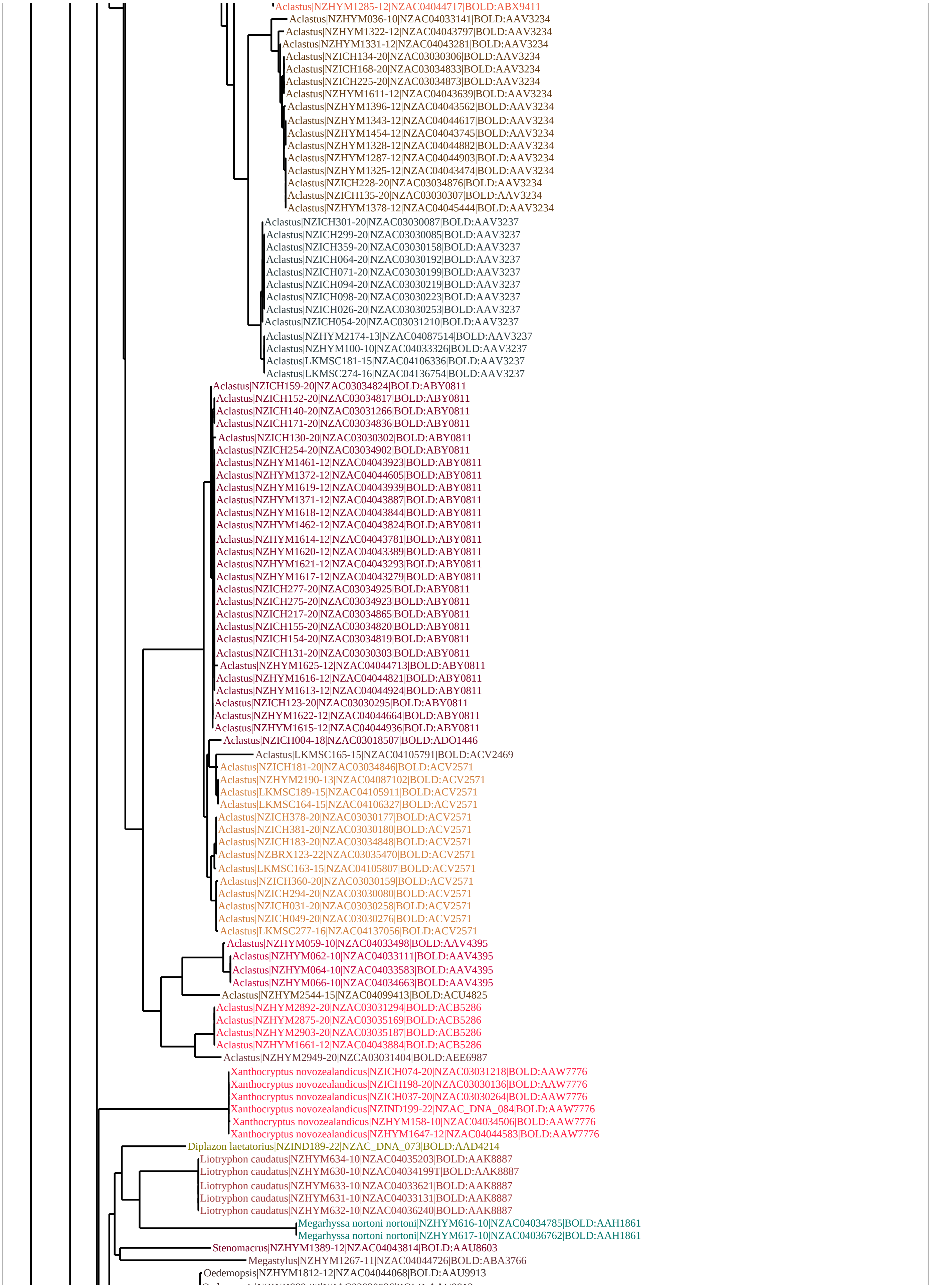
Aclastus|NZHYM1285-12|NZAC04044717|BOLD:ABX9411
Aclastus|NZHYM036-10|NZAC04033141|BOLD:AAV3234
Aclastus|NZHYM1322-12|NZAC04043797|BOLD:AAV3234
Aclastus|NZHYM1331-12|NZAC04043281|BOLD:AAV3234
Aclastus|NZICH134-20|NZAC03030306|BOLD:AAV3234
Aclastus|NZICH168-20|NZAC03034833|BOLD:AAV3234
Aclastus|NZICH225-20|NZAC03034873|BOLD:AAV3234
Aclastus|NZHYM1611-12|NZAC04043639|BOLD:AAV3234
Aclastus|NZHYM1396-12|NZAC04043562|BOLD:AAV3234
Aclastus|NZHYM1343-12|NZAC04044617|BOLD:AAV3234
Aclastus|NZHYM1454-12|NZAC04043745|BOLD:AAV3234
Aclastus|NZHYM1328-12|NZAC04044882|BOLD:AAV3234
Aclastus|NZHYM1287-12|NZAC04044903|BOLD:AAV3234
Aclastus|NZHYM1325-12|NZAC04043474|BOLD:AAV3234
Aclastus|NZICH228-20|NZAC03034876|BOLD:AAV3234
Aclastus|NZICH135-20|NZAC03030307|BOLD:AAV3234
Aclastus|NZHYM1378-12|NZAC04045444|BOLD:AAV3234
Aclastus|NZICH301-20|NZAC03030087|BOLD:AAV3237
Aclastus|NZICH299-20|NZAC03030085|BOLD:AAV3237
Aclastus|NZICH359-20|NZAC03030158|BOLD:AAV3237
Aclastus|NZICH064-20|NZAC03030192|BOLD:AAV3237
Aclastus|NZICH071-20|NZAC03030199|BOLD:AAV3237
Aclastus|NZICH094-20|NZAC03030219|BOLD:AAV3237
Aclastus|NZICH098-20|NZAC03030223|BOLD:AAV3237
Aclastus|NZICH026-20|NZAC03030253|BOLD:AAV3237
Aclastus|NZICH054-20|NZAC03031210|BOLD:AAV3237
Aclastus|NZHYM2174-13|NZAC04087514|BOLD:AAV3237
Aclastus|NZHYM100-10|NZAC04033326|BOLD:AAV3237
Aclastus|LKMSC181-15|NZAC04106336|BOLD:AAV3237
Aclastus|LKMSC274-16|NZAC04136754|BOLD:AAV3237
Aclastus|NZICH159-20|NZAC03034824|BOLD:ABY0811
Aclastus|NZICH152-20|NZAC03034817|BOLD:ABY0811
Aclastus|NZICH140-20|NZAC03031266|BOLD:ABY0811
Aclastus|NZICH171-20|NZAC03034836|BOLD:ABY0811
Aclastus|NZICH130-20|NZAC03030302|BOLD:ABY0811
Aclastus|NZICH254-20|NZAC03034902|BOLD:ABY0811
Aclastus|NZHYM1461-12|NZAC04043923|BOLD:ABY0811
Aclastus|NZHYM1372-12|NZAC04044605|BOLD:ABY0811
Aclastus|NZHYM1619-12|NZAC04043939|BOLD:ABY0811
Aclastus|NZHYM1371-12|NZAC04043887|BOLD:ABY0811
Aclastus|NZHYM1618-12|NZAC04043844|BOLD:ABY0811
Aclastus|NZHYM1462-12|NZAC04043824|BOLD:ABY0811
Aclastus|NZHYM1614-12|NZAC04043781|BOLD:ABY0811
Aclastus|NZHYM1620-12|NZAC04043389|BOLD:ABY0811
Aclastus|NZHYM1621-12|NZAC04043293|BOLD:ABY0811
Aclastus|NZHYM1617-12|NZAC04043279|BOLD:ABY0811
Aclastus|NZICH277-20|NZAC03034925|BOLD:ABY0811
Aclastus|NZICH275-20|NZAC03034923|BOLD:ABY0811
Aclastus|NZICH217-20|NZAC03034865|BOLD:ABY0811
Aclastus|NZICH155-20|NZAC03034820|BOLD:ABY0811
Aclastus|NZICH154-20|NZAC03034819|BOLD:ABY0811
Aclastus|NZICH131-20|NZAC03030303|BOLD:ABY0811
Aclastus|NZHYM1625-12|NZAC04044713|BOLD:ABY0811
Aclastus|NZHYM1616-12|NZAC04044821|BOLD:ABY0811
Aclastus|NZHYM1613-12|NZAC04044924|BOLD:ABY0811
Aclastus|NZICH123-20|NZAC03030295|BOLD:ABY0811
Aclastus|NZHYM1622-12|NZAC04044664|BOLD:ABY0811
Aclastus|NZHYM1615-12|NZAC04044936|BOLD:ABY0811
Aclastus|NZICH004-18|NZAC03018507|BOLD:ADO1446
Aclastus|LKMSC165-15|NZAC04105791|BOLD:ACV2469
Aclastus|NZICH181-20|NZAC03034846|BOLD:ACV2571
Aclastus|NZHYM2190-13|NZAC04087102|BOLD:ACV2571
Aclastus|LKMSC189-15|NZAC04105911|BOLD:ACV2571
Aclastus|LKMSC164-15|NZAC04106327|BOLD:ACV2571
Aclastus|NZICH378-20|NZAC03030177|BOLD:ACV2571
Aclastus|NZICH381-20|NZAC03030180|BOLD:ACV2571
Aclastus|NZICH183-20|NZAC03034848|BOLD:ACV2571
Aclastus|NZBRX123-22|NZAC03035470|BOLD:ACV2571
Aclastus|LKMSC163-15|NZAC04105807|BOLD:ACV2571
Aclastus|NZICH360-20|NZAC03030159|BOLD:ACV2571
Aclastus|NZICH294-20|NZAC03030080|BOLD:ACV2571
Aclastus|NZICH031-20|NZAC03030258|BOLD:ACV2571
Aclastus|NZICH049-20|NZAC03030276|BOLD:ACV2571
Aclastus|LKMSC277-16|NZAC04137056|BOLD:ACV2571
Aclastus|NZHYM059-10|NZAC04033498|BOLD:AAV4395
Aclastus|NZHYM062-10|NZAC04033111|BOLD:AAV4395
Aclastus|NZHYM064-10|NZAC04033583|BOLD:AAV4395
Aclastus|NZHYM066-10|NZAC04034663|BOLD:AAV4395
Aclastus|NZHYM2544-15|NZAC04099413|BOLD:ACU4825
Aclastus|NZHYM2892-20|NZAC03031294|BOLD:ACB5286
Aclastus|NZHYM2875-20|NZAC03035169|BOLD:ACB5286
Aclastus|NZHYM2903-20|NZAC03035187|BOLD:ACB5286
Aclastus|NZHYM1661-12|NZAC04043884|BOLD:ACB5286
Aclastus|NZHYM2949-20|NZCA03031404|BOLD:AEE6987
Xanthocryptus novozealandicus|NZICH074-20|NZAC03031218|BOLD:AAW7776
Xanthocryptus novozealandicus|NZICH198-20|NZAC03030136|BOLD:AAW7776
Xanthocryptus novozealandicus|NZICH037-20|NZAC03030264|BOLD:AAW7776
Xanthocryptus novozealandicus|NZIND199-22|NZAC_DNA_084|BOLD:AAW7776
Xanthocryptus novozealandicus|NZHYM158-10|NZAC04034506|BOLD:AAW7776
Xanthocryptus novozealandicus|NZHYM1647-12|NZAC04044583|BOLD:AAW7776
Diplazon laetatorius|NZIND189-22|NZAC_DNA_073|BOLD:AAD4214
Liotryphon caudatus|NZHYM634-10|NZAC04035203|BOLD:AAK8887
Liotryphon caudatus|NZHYM630-10|NZAC04034199T|BOLD:AAK8887
Liotryphon caudatus|NZHYM633-10|NZAC04033621|BOLD:AAK8887
Liotryphon caudatus|NZHYM631-10|NZAC04033131|BOLD:AAK8887
Liotryphon caudatus|NZHYM632-10|NZAC04036240|BOLD:AAK8887
Megarhyssa nortoni nortoni|NZHYM616-10|NZAC04034785|BOLD:AAH1861
Megarhyssa nortoni nortoni|NZHYM617-10|NZAC04036762|BOLD:AAH1861
Stenomacrus|NZHYM1389-12|NZAC04043814|BOLD:AAU8603
Megastylus|NZHYM1267-11|NZAC04044726|BOLD:ABA3766
Oedemopsis|NZHYM1812-12|NZAC04044068|BOLD:AAU9913

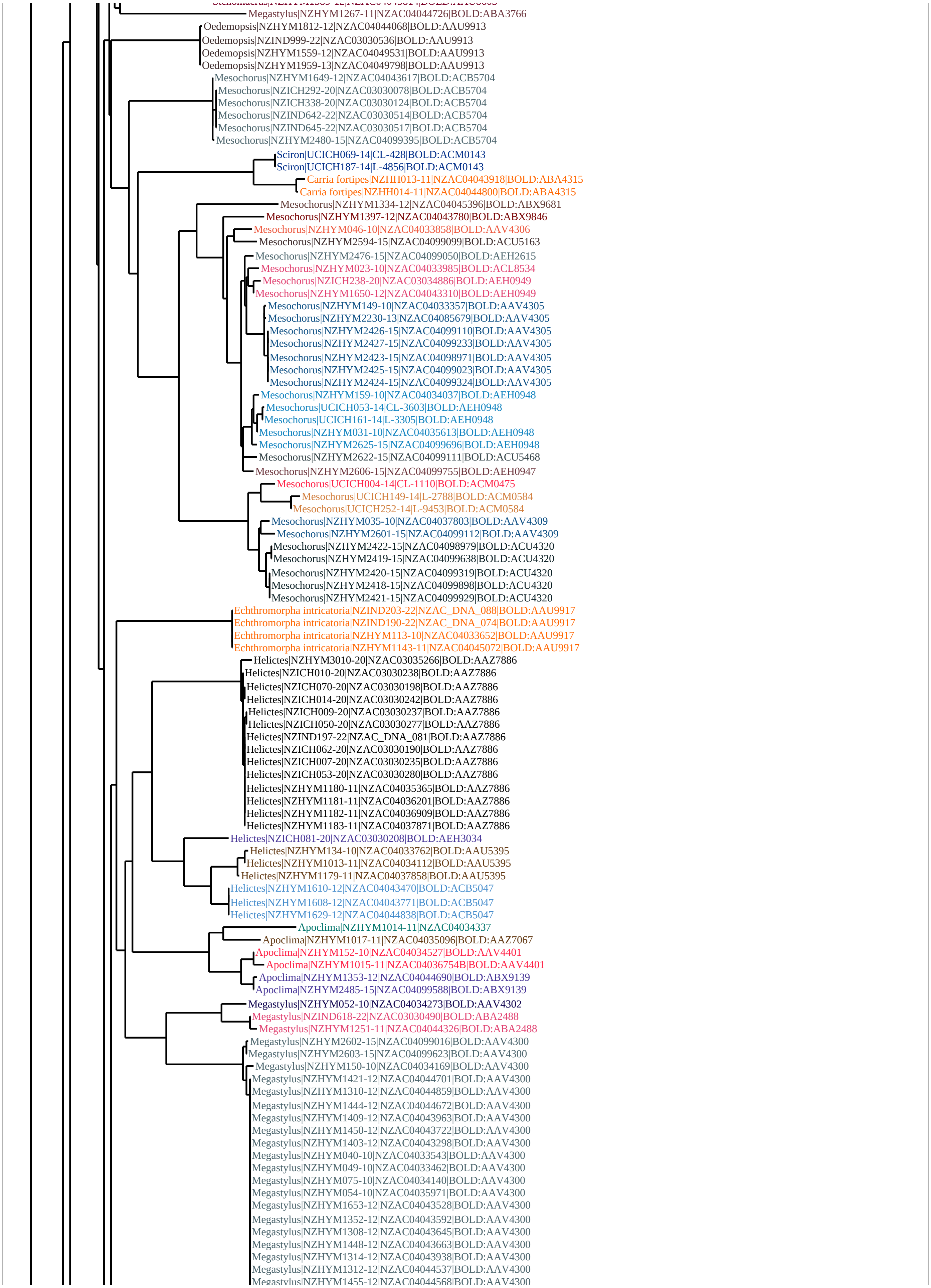
Megastylus|NZHYM1267-11|NZAC04044726|BOLD:ABA3766
Oedemopsis|NZHYM1812-12|NZAC04044068|BOLD:AAU9913
Oedemopsis|NZIND999-22|NZAC03030536|BOLD:AAU9913
Oedemopsis|NZHYM1559-12|NZAC04049531|BOLD:AAU9913
Oedemopsis|NZHYM1959-13|NZAC04049798|BOLD:AAU9913
Mesochorus|NZHYM1649-12|NZAC04043617|BOLD:ACB5704
Mesochorus|NZICH292-20|NZAC03030078|BOLD:ACB5704
Mesochorus|NZICH338-20|NZAC03030124|BOLD:ACB5704
Mesochorus|NZIND642-22|NZAC03030514|BOLD:ACB5704
Mesochorus|NZIND645-22|NZAC03030517|BOLD:ACB5704
Mesochorus|NZHYM2480-15|NZAC04099395|BOLD:ACB5704
Sciron|UCICH069-14|CL-428|BOLD:ACM0143
Sciron|UCICH187-14|L-4856|BOLD:ACM0143
Carria fortipes|NZHH013-11|NZAC04043918|BOLD:ABA4315
Carria fortipes|NZHH014-11|NZAC04044800|BOLD:ABA4315
Mesochorus|NZHYM1334-12|NZAC04045396|BOLD:ABX9681
Mesochorus|NZHYM1397-12|NZAC04043780|BOLD:ABX9846
Mesochorus|NZHYM046-10|NZAC04033858|BOLD:AAV4306
Mesochorus|NZHYM2594-15|NZAC04099099|BOLD:ACU5163
Mesochorus|NZHYM2476-15|NZAC04099050|BOLD:AEH2615
Mesochorus|NZHYM023-10|NZAC04033985|BOLD:ACL8534
Mesochorus|NZICH238-20|NZAC03034886|BOLD:AEH0949
Mesochorus|NZHYM1650-12|NZAC04043310|BOLD:AEH0949
Mesochorus|NZHYM149-10|NZAC04033357|BOLD:AAV4305
Mesochorus|NZHYM2230-13|NZAC04085679|BOLD:AAV4305
Mesochorus|NZHYM2426-15|NZAC04099110|BOLD:AAV4305
Mesochorus|NZHYM2427-15|NZAC04099233|BOLD:AAV4305
Mesochorus|NZHYM2423-15|NZAC04098971|BOLD:AAV4305
Mesochorus|NZHYM2425-15|NZAC04099023|BOLD:AAV4305
Mesochorus|NZHYM2424-15|NZAC04099324|BOLD:AAV4305
Mesochorus|NZHYM159-10|NZAC04034037|BOLD:AEH0948
Mesochorus|UCICH053-14|CL-3603|BOLD:AEH0948
Mesochorus|UCICH161-14|L-3305|BOLD:AEH0948
Mesochorus|NZHYM031-10|NZAC04035613|BOLD:AEH0948
Mesochorus|NZHYM2625-15|NZAC04099696|BOLD:AEH0948
Mesochorus|NZHYM2622-15|NZAC04099111|BOLD:ACU5468
Mesochorus|NZHYM2606-15|NZAC04099755|BOLD:AEH0947
Mesochorus|UCICH004-14|CL-1110|BOLD:ACM0475
Mesochorus|UCICH149-14|L-2788|BOLD:ACM0584
Mesochorus|UCICH252-14|L-9453|BOLD:ACM0584
Mesochorus|NZHYM035-10|NZAC04037803|BOLD:AAV4309
Mesochorus|NZHYM2601-15|NZAC04099112|BOLD:AAV4309
Mesochorus|NZHYM2422-15|NZAC04098979|BOLD:ACU4320
Mesochorus|NZHYM2419-15|NZAC04099638|BOLD:ACU4320
Mesochorus|NZHYM2420-15|NZAC04099319|BOLD:ACU4320
Mesochorus|NZHYM2418-15|NZAC04099898|BOLD:ACU4320
Mesochorus|NZHYM2421-15|NZAC04099929|BOLD:ACU4320
Echthromorpha intricatoria|NZIND203-22|NZAC_DNA_088|BOLD:AAU9917
Echthromorpha intricatoria|NZIND190-22|NZAC_DNA_074|BOLD:AAU9917
Echthromorpha intricatoria|NZHYM113-10|NZAC04033652|BOLD:AAU9917
Echthromorpha intricatoria|NZHYM1143-11|NZAC04045072|BOLD:AAU9917
Helictes|NZHYM3010-20|NZAC03035266|BOLD:AAZ7886
Helictes|NZICH010-20|NZAC03030238|BOLD:AAZ7886
Helictes|NZICH070-20|NZAC03030198|BOLD:AAZ7886
Helictes|NZICH014-20|NZAC03030242|BOLD:AAZ7886
Helictes|NZICH009-20|NZAC03030237|BOLD:AAZ7886
Helictes|NZICH050-20|NZAC03030277|BOLD:AAZ7886
Helictes|NZIND197-22|NZAC_DNA_081|BOLD:AAZ7886
Helictes|NZICH062-20|NZAC03030190|BOLD:AAZ7886
Helictes|NZICH007-20|NZAC03030235|BOLD:AAZ7886
Helictes|NZICH053-20|NZAC03030280|BOLD:AAZ7886
Helictes|NZHYM1180-11|NZAC04035365|BOLD:AAZ7886
Helictes|NZHYM1181-11|NZAC04036201|BOLD:AAZ7886
Helictes|NZHYM1182-11|NZAC04036909|BOLD:AAZ7886
Helictes|NZHYM1183-11|NZAC04037871|BOLD:AAZ7886
Helictes|NZICH081-20|NZAC03030208|BOLD:AEH3034
Helictes|NZHYM134-10|NZAC04033762|BOLD:AAU5395
Helictes|NZHYM1013-11|NZAC04034112|BOLD:AAU5395
Helictes|NZHYM1179-11|NZAC04037858|BOLD:AAU5395
Helictes|NZHYM1610-12|NZAC04043470|BOLD:ACB5047
Helictes|NZHYM1608-12|NZAC04043771|BOLD:ACB5047
Helictes|NZHYM1629-12|NZAC04044838|BOLD:ACB5047
Apoclima|NZHYM1014-11|NZAC04034337
Apoclima|NZHYM1017-11|NZAC04035096|BOLD:AAZ7067
Apoclima|NZHYM152-10|NZAC04034527|BOLD:AAV4401
Apoclima|NZHYM1015-11|NZAC04036754B|BOLD:AAV4401
Apoclima|NZHYM1353-12|NZAC04044690|BOLD:ABX9139
Apoclima|NZHYM2485-15|NZAC04099588|BOLD:ABX9139
Megastylus|NZHYM052-10|NZAC04034273|BOLD:AAV4302
Megastylus|NZIND618-22|NZAC03030490|BOLD:ABA2488
Megastylus|NZHYM1251-11|NZAC04044326|BOLD:ABA2488
Megastylus|NZHYM2602-15|NZAC04099016|BOLD:AAV4300
Megastylus|NZHYM2603-15|NZAC04099623|BOLD:AAV4300
Megastylus|NZHYM150-10|NZAC04034169|BOLD:AAV4300
Megastylus|NZHYM1421-12|NZAC04044701|BOLD:AAV4300
Megastylus|NZHYM1310-12|NZAC04044859|BOLD:AAV4300
Megastylus|NZHYM1444-12|NZAC04044672|BOLD:AAV4300
Megastylus|NZHYM1409-12|NZAC04043963|BOLD:AAV4300
Megastylus|NZHYM1450-12|NZAC04043722|BOLD:AAV4300
Megastylus|NZHYM1403-12|NZAC04043298|BOLD:AAV4300
Megastylus|NZHYM040-10|NZAC04033543|BOLD:AAV4300
Megastylus|NZHYM049-10|NZAC04033462|BOLD:AAV4300
Megastylus|NZHYM075-10|NZAC04034140|BOLD:AAV4300
Megastylus|NZHYM054-10|NZAC04035971|BOLD:AAV4300
Megastylus|NZHYM1653-12|NZAC04043528|BOLD:AAV4300
Megastylus|NZHYM1352-12|NZAC04043592|BOLD:AAV4300
Megastylus|NZHYM1308-12|NZAC04043645|BOLD:AAV4300
Megastylus|NZHYM1448-12|NZAC04043663|BOLD:AAV4300
Megastylus|NZHYM1314-12|NZAC04043938|BOLD:AAV4300
Megastylus|NZHYM1312-12|NZAC04044537|BOLD:AAV4300
Megastylus|NZHYM1455-12|NZAC04044568|BOLD:AAV4300

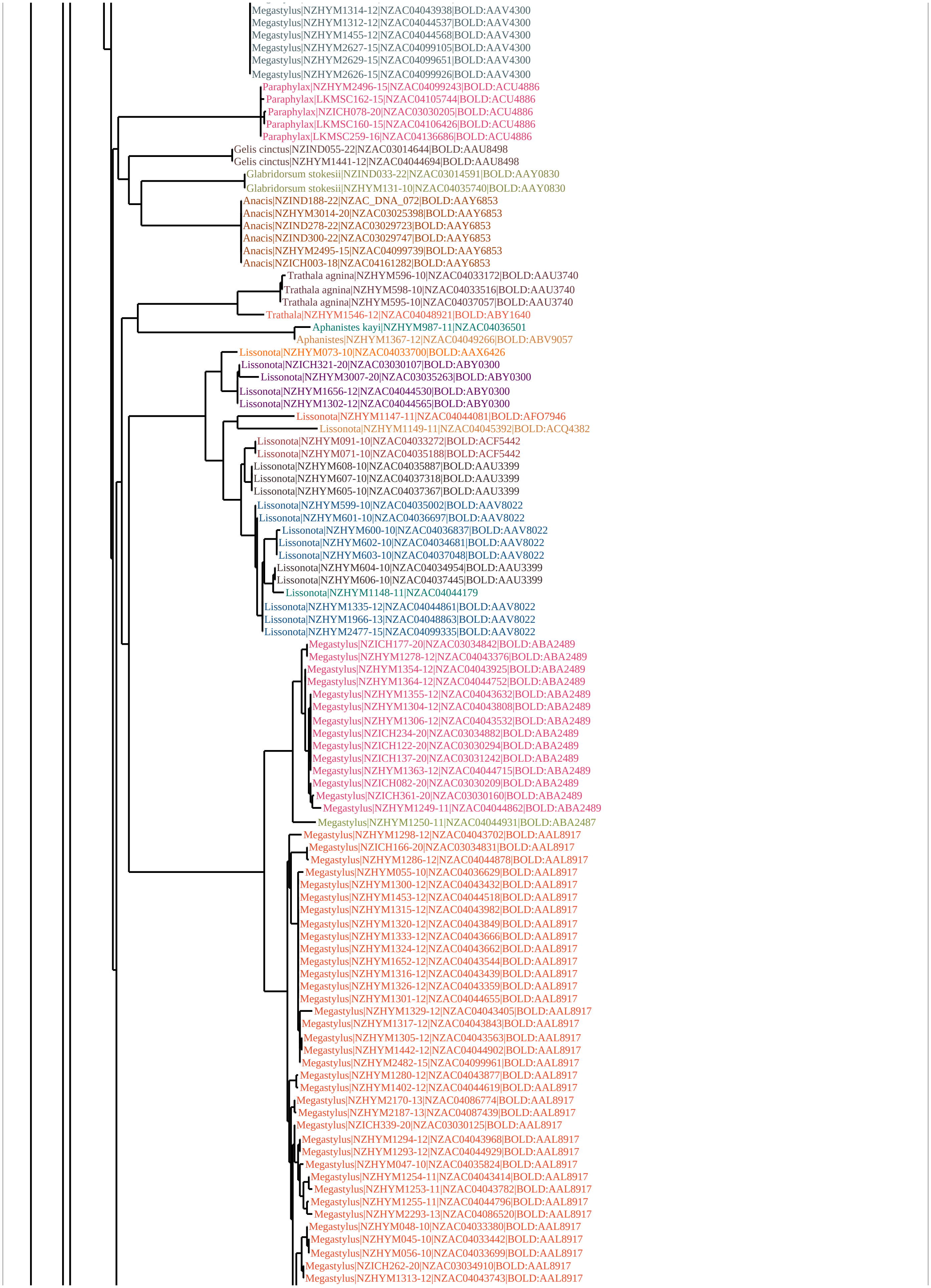
Megastylus|NZHYM1314-12|NZAC04043938|BOLD:AAV4300
Megastylus|NZHYM1312-12|NZAC04044537|BOLD:AAV4300
Megastylus|NZHYM1455-12|NZAC04044568|BOLD:AAV4300
Megastylus|NZHYM2627-15|NZAC04099105|BOLD:AAV4300
Megastylus|NZHYM2629-15|NZAC04099651|BOLD:AAV4300
Megastylus|NZHYM2626-15|NZAC04099926|BOLD:AAV4300
Paraphylax|NZHYM2496-15|NZAC04099243|BOLD:ACU4886
Paraphylax|LKMSC162-15|NZAC04105744|BOLD:ACU4886
Paraphylax|NZICH078-20|NZAC03030205|BOLD:ACU4886
Paraphylax|LKMSC160-15|NZAC04106426|BOLD:ACU4886
Paraphylax|LKMSC259-16|NZAC04136686|BOLD:ACU4886
Gelis cinctus|NZIND055-22|NZAC03014644|BOLD:AAU8498
Gelis cinctus|NZHYM1441-12|NZAC04044694|BOLD:AAU8498
Glabridorsum stokesii|NZIND033-22|NZAC03014591|BOLD:AAY0830
Glabridorsum stokesii|NZHYM131-10|NZAC04035740|BOLD:AAY0830
Anacis|NZIND188-22|NZAC_DNA_072|BOLD:AAY6853
Anacis|NZHYM3014-20|NZAC03025398|BOLD:AAY6853
Anacis|NZIND278-22|NZAC03029723|BOLD:AAY6853
Anacis|NZIND300-22|NZAC03029747|BOLD:AAY6853
Anacis|NZHYM2495-15|NZAC04099739|BOLD:AAY6853
Anacis|NZICH003-18|NZAC04161282|BOLD:AAY6853
Trathala agnina|NZHYM596-10|NZAC04033172|BOLD:AAU3740
Trathala agnina|NZHYM598-10|NZAC04033516|BOLD:AAU3740
Trathala agnina|NZHYM595-10|NZAC04037057|BOLD:AAU3740
Trathala|NZHYM1546-12|NZAC04048921|BOLD:ABY1640
Aphanistes kayi|NZHYM987-11|NZAC04036501
Aphanistes|NZHYM1367-12|NZAC04049266|BOLD:ABV9057
Lissonota|NZHYM073-10|NZAC04033700|BOLD:AAX6426
Lissonota|NZICH321-20|NZAC03030107|BOLD:ABY0300
Lissonota|NZHYM3007-20|NZAC03035263|BOLD:ABY0300
Lissonota|NZHYM1656-12|NZAC04044530|BOLD:ABY0300
Lissonota|NZHYM1302-12|NZAC04044565|BOLD:ABY0300
Lissonota|NZHYM1147-11|NZAC04044081|BOLD:AFO7946
Lissonota|NZHYM1149-11|NZAC04045392|BOLD:ACQ4382
Lissonota|NZHYM091-10|NZAC04033272|BOLD:ACF5442
Lissonota|NZHYM071-10|NZAC04035188|BOLD:ACF5442
Lissonota|NZHYM608-10|NZAC04035887|BOLD:AAU3399
Lissonota|NZHYM607-10|NZAC04037318|BOLD:AAU3399
Lissonota|NZHYM605-10|NZAC04037367|BOLD:AAU3399
Lissonota|NZHYM599-10|NZAC04035002|BOLD:AAV8022
Lissonota|NZHYM601-10|NZAC04036697|BOLD:AAV8022
Lissonota|NZHYM600-10|NZAC04036837|BOLD:AAV8022
Lissonota|NZHYM602-10|NZAC04034681|BOLD:AAV8022
Lissonota|NZHYM603-10|NZAC04037048|BOLD:AAV8022
Lissonota|NZHYM604-10|NZAC04034954|BOLD:AAU3399
Lissonota|NZHYM606-10|NZAC04037445|BOLD:AAU3399
Lissonota|NZHYM1148-11|NZAC04044179
Lissonota|NZHYM1335-12|NZAC04044861|BOLD:AAV8022
Lissonota|NZHYM1966-13|NZAC04048863|BOLD:AAV8022
Lissonota|NZHYM2477-15|NZAC04099335|BOLD:AAV8022
Megastylus|NZICH177-20|NZAC03034842|BOLD:ABA2489
Megastylus|NZHYM1278-12|NZAC04043376|BOLD:ABA2489
Megastylus|NZHYM1354-12|NZAC04043925|BOLD:ABA2489
Megastylus|NZHYM1364-12|NZAC04044752|BOLD:ABA2489
Megastylus|NZHYM1355-12|NZAC04043632|BOLD:ABA2489
Megastylus|NZHYM1304-12|NZAC04043808|BOLD:ABA2489
Megastylus|NZHYM1306-12|NZAC04043532|BOLD:ABA2489
Megastylus|NZICH234-20|NZAC03034882|BOLD:ABA2489
Megastylus|NZICH122-20|NZAC03030294|BOLD:ABA2489
Megastylus|NZICH137-20|NZAC03031242|BOLD:ABA2489
Megastylus|NZHYM1363-12|NZAC04044715|BOLD:ABA2489
Megastylus|NZICH082-20|NZAC03030209|BOLD:ABA2489
Megastylus|NZICH361-20|NZAC03030160|BOLD:ABA2489
Megastylus|NZHYM1249-11|NZAC04044862|BOLD:ABA2489
Megastylus|NZHYM1250-11|NZAC04044931|BOLD:ABA2487
Megastylus|NZHYM1298-12|NZAC04043702|BOLD:AAL8917
Megastylus|NZICH166-20|NZAC03034831|BOLD:AAL8917
Megastylus|NZHYM1286-12|NZAC04044878|BOLD:AAL8917
Megastylus|NZHYM055-10|NZAC04036629|BOLD:AAL8917
Megastylus|NZHYM1300-12|NZAC04043432|BOLD:AAL8917
Megastylus|NZHYM1453-12|NZAC04044518|BOLD:AAL8917
Megastylus|NZHYM1315-12|NZAC04043982|BOLD:AAL8917
Megastylus|NZHYM1320-12|NZAC04043849|BOLD:AAL8917
Megastylus|NZHYM1333-12|NZAC04043666|BOLD:AAL8917
Megastylus|NZHYM1324-12|NZAC04043662|BOLD:AAL8917
Megastylus|NZHYM1652-12|NZAC04043544|BOLD:AAL8917
Megastylus|NZHYM1316-12|NZAC04043439|BOLD:AAL8917
Megastylus|NZHYM1326-12|NZAC04043359|BOLD:AAL8917
Megastylus|NZHYM1301-12|NZAC04044655|BOLD:AAL8917
Megastylus|NZHYM1329-12|NZAC04043405|BOLD:AAL8917
Megastylus|NZHYM1317-12|NZAC04043843|BOLD:AAL8917
Megastylus|NZHYM1305-12|NZAC04043563|BOLD:AAL8917
Megastylus|NZHYM1442-12|NZAC04044902|BOLD:AAL8917
Megastylus|NZHYM2482-15|NZAC04099961|BOLD:AAL8917
Megastylus|NZHYM1280-12|NZAC04043877|BOLD:AAL8917
Megastylus|NZHYM1402-12|NZAC04044619|BOLD:AAL8917
Megastylus|NZHYM2170-13|NZAC04086774|BOLD:AAL8917
Megastylus|NZHYM2187-13|NZAC04087439|BOLD:AAL8917
Megastylus|NZICH339-20|NZAC03030125|BOLD:AAL8917
Megastylus|NZHYM1294-12|NZAC04043968|BOLD:AAL8917
Megastylus|NZHYM1293-12|NZAC04044929|BOLD:AAL8917
Megastylus|NZHYM047-10|NZAC04035824|BOLD:AAL8917
Megastylus|NZHYM1254-11|NZAC04043414|BOLD:AAL8917
Megastylus|NZHYM1253-11|NZAC04043782|BOLD:AAL8917
Megastylus|NZHYM1255-11|NZAC04044796|BOLD:AAL8917
Megastylus|NZHYM2293-13|NZAC04086520|BOLD:AAL8917
Megastylus|NZHYM048-10|NZAC04033380|BOLD:AAL8917
Megastylus|NZHYM045-10|NZAC04033442|BOLD:AAL8917
Megastylus|NZHYM056-10|NZAC04033699|BOLD:AAL8917
Megastylus|NZICH262-20|NZAC03034910|BOLD:AAL8917
Megastylus|NZHYM1313-12|NZAC04043743|BOLD:AAL8917

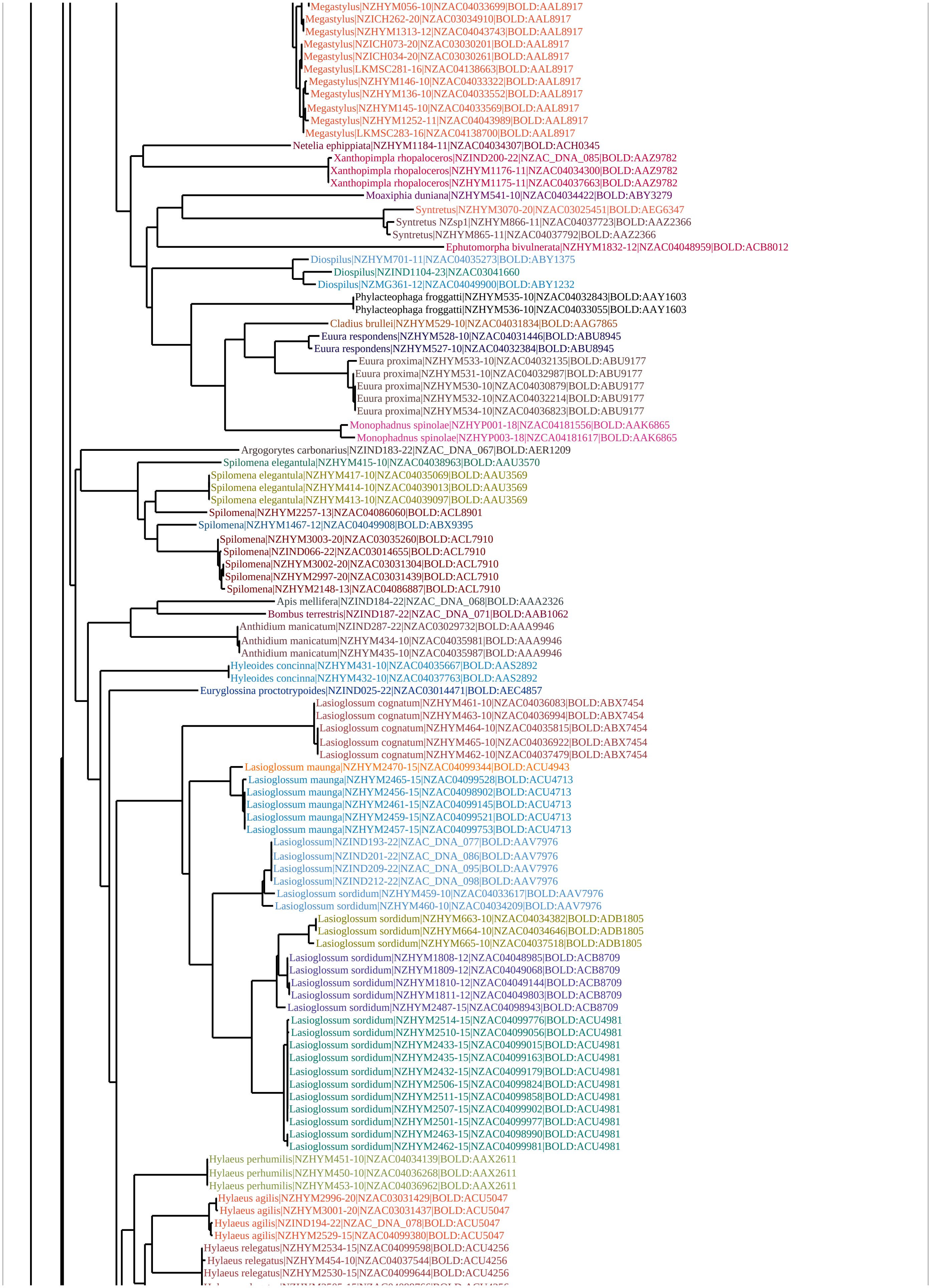
Megastylus|NZHYM056-10|NZAC04033699|BOLD:AAL8917
Megastylus|NZICH262-20|NZAC03034910|BOLD:AAL8917
Megastylus|NZHYM1313-12|NZAC04043743|BOLD:AAL8917
Megastylus|NZICH073-20|NZAC03030201|BOLD:AAL8917
Megastylus|NZICH034-20|NZAC03030261|BOLD:AAL8917
Megastylus|LKMSC281-16|NZAC04138663|BOLD:AAL8917
Megastylus|NZHYM146-10|NZAC04033322|BOLD:AAL8917
Megastylus|NZHYM136-10|NZAC04033552|BOLD:AAL8917
Megastylus|NZHYM145-10|NZAC04033569|BOLD:AAL8917
Megastylus|NZHYM1252-11|NZAC04043989|BOLD:AAL8917
Megastylus|LKMSC283-16|NZAC04138700|BOLD:AAL8917
Netelia ephippiata|NZHYM1184-11|NZAC04034307|BOLD:ACH0345
Xanthopimpla rhopaloceros|NZIND200-22|NZAC_DNA_085|BOLD:AAZ9782
Xanthopimpla rhopaloceros|NZHYM1176-11|NZAC04034300|BOLD:AAZ9782
Xanthopimpla rhopaloceros|NZHYM1175-11|NZAC04037663|BOLD:AAZ9782
Moaxiphia duniana|NZHYM541-10|NZAC04034422|BOLD:ABY3279
Syntretus|NZHYM3070-20|NZAC03025451|BOLD:AEG6347
Syntretus NZsp1|NZHYM866-11|NZAC04037723|BOLD:AAZ2366
Syntretus|NZHYM865-11|NZAC04037792|BOLD:AAZ2366
Ephutomorpha bivulnerata|NZHYM1832-12|NZAC04048959|BOLD:ACB8012
Diospilus|NZHYM701-11|NZAC04035273|BOLD:ABY1375
Diospilus|NZIND1104-23|NZAC03041660
Diospilus|NZMG361-12|NZAC04049900|BOLD:ABY1232
Phylacteophaga froggatti|NZHYM535-10|NZAC04032843|BOLD:AAY1603
Phylacteophaga froggatti|NZHYM536-10|NZAC04033055|BOLD:AAY1603
Cladius brullei|NZHYM529-10|NZAC04031834|BOLD:AAG7865
Euura respondens|NZHYM528-10|NZAC04031446|BOLD:ABU8945
Euura respondens|NZHYM527-10|NZAC04032384|BOLD:ABU8945
Euura proxima|NZHYM533-10|NZAC04032135|BOLD:ABU9177
Euura proxima|NZHYM531-10|NZAC04032987|BOLD:ABU9177
Euura proxima|NZHYM530-10|NZAC04030879|BOLD:ABU9177
Euura proxima|NZHYM532-10|NZAC04032214|BOLD:ABU9177
Euura proxima|NZHYM534-10|NZAC04036823|BOLD:ABU9177
Monophadnus spinolae|NZHYP001-18|NZAC04181556|BOLD:AAK6865
Monophadnus spinolae|NZHYP003-18|NZCA04181617|BOLD:AAK6865
Argogorytes carbonarius|NZIND183-22|NZAC_DNA_067|BOLD:AER1209
Spilomena elegantula|NZHYM415-10|NZAC04038963|BOLD:AAU3570
Spilomena elegantula|NZHYM417-10|NZAC04035069|BOLD:AAU3569
Spilomena elegantula|NZHYM414-10|NZAC04039013|BOLD:AAU3569
Spilomena elegantula|NZHYM413-10|NZAC04039097|BOLD:AAU3569
Spilomena|NZHYM2257-13|NZAC04086060|BOLD:ACL8901
Spilomena|NZHYM1467-12|NZAC04049908|BOLD:ABX9395
Spilomena|NZHYM3003-20|NZAC03035260|BOLD:ACL7910
Spilomena|NZIND066-22|NZAC03014655|BOLD:ACL7910
Spilomena|NZHYM3002-20|NZAC03031304|BOLD:ACL7910
Spilomena|NZHYM2997-20|NZAC03031439|BOLD:ACL7910
Spilomena|NZHYM2148-13|NZAC04086887|BOLD:ACL7910
Apis mellifera|NZIND184-22|NZAC_DNA_068|BOLD:AAA2326
Bombus terrestris|NZIND187-22|NZAC_DNA_071|BOLD:AAB1062
Anthidium manicatum|NZIND287-22|NZAC03029732|BOLD:AAA9946
Anthidium manicatum|NZHYM434-10|NZAC04035981|BOLD:AAA9946
Anthidium manicatum|NZHYM435-10|NZAC04035987|BOLD:AAA9946
Hyleoides concinna|NZHYM431-10|NZAC04035667|BOLD:AAS2892
Hyleoides concinna|NZHYM432-10|NZAC04037763|BOLD:AAS2892
Euryglossina proctotrypoides|NZIND025-22|NZAC03014471|BOLD:AEC4857
Lasioglossum cognatum|NZHYM461-10|NZAC04036083|BOLD:ABX7454
Lasioglossum cognatum|NZHYM463-10|NZAC04036994|BOLD:ABX7454
Lasioglossum cognatum|NZHYM464-10|NZAC04035815|BOLD:ABX7454
Lasioglossum cognatum|NZHYM465-10|NZAC04036922|BOLD:ABX7454
Lasioglossum cognatum|NZHYM462-10|NZAC04037479|BOLD:ABX7454
Lasioglossum maunga|NZHYM2470-15|NZAC04099344|BOLD:ACU4943
Lasioglossum maunga|NZHYM2465-15|NZAC04099528|BOLD:ACU4713
Lasioglossum maunga|NZHYM2456-15|NZAC04098902|BOLD:ACU4713
Lasioglossum maunga|NZHYM2461-15|NZAC04099145|BOLD:ACU4713
Lasioglossum maunga|NZHYM2459-15|NZAC04099521|BOLD:ACU4713
Lasioglossum maunga|NZHYM2457-15|NZAC04099753|BOLD:ACU4713
Lasioglossum|NZIND193-22|NZAC_DNA_077|BOLD:AAV7976
Lasioglossum|NZIND201-22|NZAC_DNA_086|BOLD:AAV7976
Lasioglossum|NZIND209-22|NZAC_DNA_095|BOLD:AAV7976
Lasioglossum|NZIND212-22|NZAC_DNA_098|BOLD:AAV7976
Lasioglossum sordidum|NZHYM459-10|NZAC04033617|BOLD:AAV7976
Lasioglossum sordidum|NZHYM460-10|NZAC04034209|BOLD:AAV7976
Lasioglossum sordidum|NZHYM663-10|NZAC04034382|BOLD:ADB1805
Lasioglossum sordidum|NZHYM664-10|NZAC04034646|BOLD:ADB1805
Lasioglossum sordidum|NZHYM665-10|NZAC04037518|BOLD:ADB1805
Lasioglossum sordidum|NZHYM1808-12|NZAC04048985|BOLD:ACB8709
Lasioglossum sordidum|NZHYM1809-12|NZAC04049068|BOLD:ACB8709
Lasioglossum sordidum|NZHYM1810-12|NZAC04049144|BOLD:ACB8709
Lasioglossum sordidum|NZHYM1811-12|NZAC04049803|BOLD:ACB8709
Lasioglossum sordidum|NZHYM2487-15|NZAC04098943|BOLD:ACB8709
Lasioglossum sordidum|NZHYM2514-15|NZAC04099776|BOLD:ACU4981
Lasioglossum sordidum|NZHYM2510-15|NZAC04099056|BOLD:ACU4981
Lasioglossum sordidum|NZHYM2433-15|NZAC04099015|BOLD:ACU4981
Lasioglossum sordidum|NZHYM2435-15|NZAC04099163|BOLD:ACU4981
Lasioglossum sordidum|NZHYM2432-15|NZAC04099179|BOLD:ACU4981
Lasioglossum sordidum|NZHYM2506-15|NZAC04099824|BOLD:ACU4981
Lasioglossum sordidum|NZHYM2511-15|NZAC04099858|BOLD:ACU4981
Lasioglossum sordidum|NZHYM2507-15|NZAC04099902|BOLD:ACU4981
Lasioglossum sordidum|NZHYM2501-15|NZAC04099977|BOLD:ACU4981
Lasioglossum sordidum|NZHYM2463-15|NZAC04098990|BOLD:ACU4981
Lasioglossum sordidum|NZHYM2462-15|NZAC04099981|BOLD:ACU4981
Hylaeus perhumilis|NZHYM451-10|NZAC04034139|BOLD:AAX2611
Hylaeus perhumilis|NZHYM450-10|NZAC04036268|BOLD:AAX2611
Hylaeus perhumilis|NZHYM453-10|NZAC04036962|BOLD:AAX2611
Hylaeus agilis|NZHYM2996-20|NZAC03031429|BOLD:ACU5047
Hylaeus agilis|NZHYM3001-20|NZAC03031437|BOLD:ACU5047
Hylaeus agilis|NZIND194-22|NZAC_DNA_078|BOLD:ACU5047
Hylaeus agilis|NZHYM2529-15|NZAC04099380|BOLD:ACU5047
Hylaeus relegatus|NZHYM2534-15|NZAC04099598|BOLD:ACU4256
Hylaeus relegatus|NZHYM454-10|NZAC04037544|BOLD:ACU4256
Hylaeus relegatus|NZHYM2530-15|NZAC04099644|BOLD:ACU4256

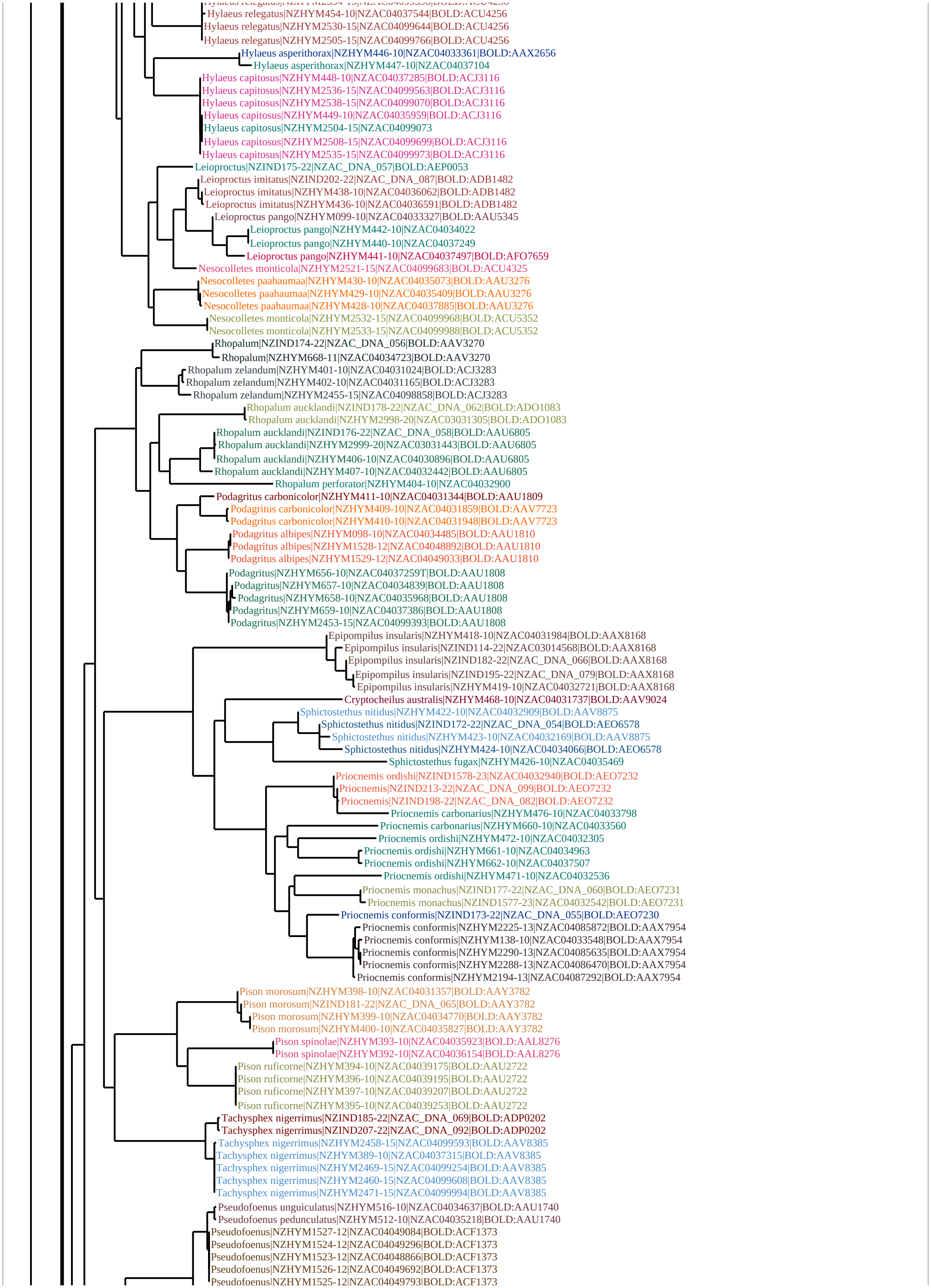
Hylaeus relegatus|NZHYM454-10|NZAC04037544|BOLD:ACU4256
Hylaeus relegatus|NZHYM2530-15|NZAC04099644|BOLD:ACU4256
Hylaeus relegatus|NZHYM2505-15|NZAC04099766|BOLD:ACU4256
Hylaeus asperithorax|NZHYM446-10|NZAC04033361|BOLD:AAX2656
Hylaeus asperithorax|NZHYM447-10|NZAC04037104
Hylaeus capitosus|NZHYM448-10|NZAC04037285|BOLD:ACJ3116
Hylaeus capitosus|NZHYM2536-15|NZAC04099563|BOLD:ACJ3116
Hylaeus capitosus|NZHYM2538-15|NZAC04099070|BOLD:ACJ3116
Hylaeus capitosus|NZHYM449-10|NZAC04035959|BOLD:ACJ3116
Hylaeus capitosus|NZHYM2504-15|NZAC04099073
Hylaeus capitosus|NZHYM2508-15|NZAC04099699|BOLD:ACJ3116
Hylaeus capitosus|NZHYM2535-15|NZAC04099973|BOLD:ACJ3116
Leioproctus|NZIND175-22|NZAC_DNA_057|BOLD:AEP0053
Leioproctus imitatus|NZIND202-22|NZAC_DNA_087|BOLD:ADB1482
Leioproctus imitatus|NZHYM438-10|NZAC04036062|BOLD:ADB1482
Leioproctus imitatus|NZHYM436-10|NZAC04036591|BOLD:ADB1482
Leioproctus pango|NZHYM099-10|NZAC04033327|BOLD:AAU5345
Leioproctus pango|NZHYM442-10|NZAC04034022
Leioproctus pango|NZHYM440-10|NZAC04037249
Leioproctus pango|NZHYM441-10|NZAC04037497|BOLD:AFO7659
Nesocolletes monticola|NZHYM2521-15|NZAC04099683|BOLD:ACU4325
Nesocolletes paahaumaa|NZHYM430-10|NZAC04035073|BOLD:AAU3276
Nesocolletes paahaumaa|NZHYM429-10|NZAC04035409|BOLD:AAU3276
Nesocolletes paahaumaa|NZHYM428-10|NZAC04037885|BOLD:AAU3276
Nesocolletes monticola|NZHYM2532-15|NZAC04099968|BOLD:ACU5352
Nesocolletes monticola|NZHYM2533-15|NZAC04099988|BOLD:ACU5352
Rhopalum|NZIND174-22|NZAC_DNA_056|BOLD:AAV3270
Rhopalum|NZHYM668-11|NZAC04034723|BOLD:AAV3270
Rhopalum zelandum|NZHYM401-10|NZAC04031024|BOLD:ACJ3283
Rhopalum zelandum|NZHYM402-10|NZAC04031165|BOLD:ACJ3283
Rhopalum zelandum|NZHYM2455-15|NZAC04098858|BOLD:ACJ3283
Rhopalum aucklandi|NZIND178-22|NZAC_DNA_062|BOLD:ADO1083
Rhopalum aucklandi|NZHYM2998-20|NZAC03031305|BOLD:ADO1083
Rhopalum aucklandi|NZIND176-22|NZAC_DNA_058|BOLD:AAU6805
Rhopalum aucklandi|NZHYM2999-20|NZAC03031443|BOLD:AAU6805
Rhopalum aucklandi|NZHYM406-10|NZAC04030896|BOLD:AAU6805
Rhopalum aucklandi|NZHYM407-10|NZAC04032442|BOLD:AAU6805
Rhopalum perforator|NZHYM404-10|NZAC04032900
Podagritus carbonicolor|NZHYM411-10|NZAC04031344|BOLD:AAU1809
Podagritus carbonicolor|NZHYM409-10|NZAC04031859|BOLD:AAV7723
Podagritus carbonicolor|NZHYM410-10|NZAC04031948|BOLD:AAV7723
Podagritus albipes|NZHYM098-10|NZAC04034485|BOLD:AAU1810
Podagritus albipes|NZHYM1528-12|NZAC04048892|BOLD:AAU1810
Podagritus albipes|NZHYM1529-12|NZAC04049033|BOLD:AAU1810
Podagritus|NZHYM656-10|NZAC04037259T|BOLD:AAU1808
Podagritus|NZHYM657-10|NZAC04034839|BOLD:AAU1808
Podagritus|NZHYM658-10|NZAC04035968|BOLD:AAU1808
Podagritus|NZHYM659-10|NZAC04037386|BOLD:AAU1808
Podagritus|NZHYM2453-15|NZAC04099393|BOLD:AAU1808
Epipompilus insularis|NZHYM418-10|NZAC04031984|BOLD:AAX8168
Epipompilus insularis|NZIND114-22|NZAC03014568|BOLD:AAX8168
Epipompilus insularis|NZIND182-22|NZAC_DNA_066|BOLD:AAX8168
Epipompilus insularis|NZIND195-22|NZAC_DNA_079|BOLD:AAX8168
Epipompilus insularis|NZHYM419-10|NZAC04032721|BOLD:AAX8168
Cryptocheilus australis|NZHYM468-10|NZAC04031737|BOLD:AAV9024
Sphictostethus nitidus|NZHYM422-10|NZAC04032909|BOLD:AAV8875
Sphictostethus nitidus|NZIND172-22|NZAC_DNA_054|BOLD:AEO6578
Sphictostethus nitidus|NZHYM423-10|NZAC04032169|BOLD:AAV8875
Sphictostethus nitidus|NZHYM424-10|NZAC04034066|BOLD:AEO6578
Sphictostethus fugax|NZHYM426-10|NZAC04035469
Priocnemis ordishi|NZIND1578-23|NZAC04032940|BOLD:AEO7232
Priocnemis|NZIND213-22|NZAC_DNA_099|BOLD:AEO7232
Priocnemis|NZIND198-22|NZAC_DNA_082|BOLD:AEO7232
Priocnemis carbonarius|NZHYM476-10|NZAC04033798
Priocnemis carbonarius|NZHYM660-10|NZAC04033560
Priocnemis ordishi|NZHYM472-10|NZAC04032305
Priocnemis ordishi|NZHYM661-10|NZAC04034963
Priocnemis ordishi|NZHYM662-10|NZAC04037507
Priocnemis ordishi|NZHYM471-10|NZAC04032536
Priocnemis monachus|NZIND177-22|NZAC_DNA_060|BOLD:AEO7231
Priocnemis monachus|NZIND1577-23|NZAC04032542|BOLD:AEO7231
Priocnemis conformis|NZIND173-22|NZAC_DNA_055|BOLD:AEO7230
Priocnemis conformis|NZHYM2225-13|NZAC04085872|BOLD:AAX7954
Priocnemis conformis|NZHYM138-10|NZAC04033548|BOLD:AAX7954
Priocnemis conformis|NZHYM2290-13|NZAC04085635|BOLD:AAX7954
Priocnemis conformis|NZHYM2288-13|NZAC04086470|BOLD:AAX7954
Priocnemis conformis|NZHYM2194-13|NZAC04087292|BOLD:AAX7954
Pison morosum|NZHYM398-10|NZAC04031357|BOLD:AAY3782
Pison morosum|NZIND181-22|NZAC_DNA_065|BOLD:AAY3782
Pison morosum|NZHYM399-10|NZAC04034770|BOLD:AAY3782
Pison morosum|NZHYM400-10|NZAC04035827|BOLD:AAY3782
Pison spinolae|NZHYM393-10|NZAC04035923|BOLD:AAL8276
Pison spinolae|NZHYM392-10|NZAC04036154|BOLD:AAL8276
Pison ruficorne|NZHYM394-10|NZAC04039175|BOLD:AAU2722
Pison ruficorne|NZHYM396-10|NZAC04039195|BOLD:AAU2722
Pison ruficorne|NZHYM397-10|NZAC04039207|BOLD:AAU2722
Pison ruficorne|NZHYM395-10|NZAC04039253|BOLD:AAU2722
Tachysphex nigerrimus|NZIND185-22|NZAC_DNA_069|BOLD:ADP0202
Tachysphex nigerrimus|NZIND207-22|NZAC_DNA_092|BOLD:ADP0202
Tachysphex nigerrimus|NZHYM2458-15|NZAC04099593|BOLD:AAV8385
Tachysphex nigerrimus|NZHYM389-10|NZAC04037315|BOLD:AAV8385
Tachysphex nigerrimus|NZHYM2469-15|NZAC04099254|BOLD:AAV8385
Tachysphex nigerrimus|NZHYM2460-15|NZAC04099608|BOLD:AAV8385
Tachysphex nigerrimus|NZHYM2471-15|NZAC04099994|BOLD:AAV8385
Pseudofoenus unguiculatus|NZHYM516-10|NZAC04034637|BOLD:AAU1740
Pseudofoenus pedunculatus|NZHYM512-10|NZAC04035218|BOLD:AAU1740
Pseudofoenus|NZHYM1527-12|NZAC04049084|BOLD:ACF1373
Pseudofoenus|NZHYM1524-12|NZAC04049296|BOLD:ACF1373
Pseudofoenus|NZHYM1523-12|NZAC04048866|BOLD:ACF1373
Pseudofoenus|NZHYM1526-12|NZAC04049692|BOLD:ACF1373
Pseudofoenus|NZHYM1525-12|NZAC04049793|BOLD:ACF1373

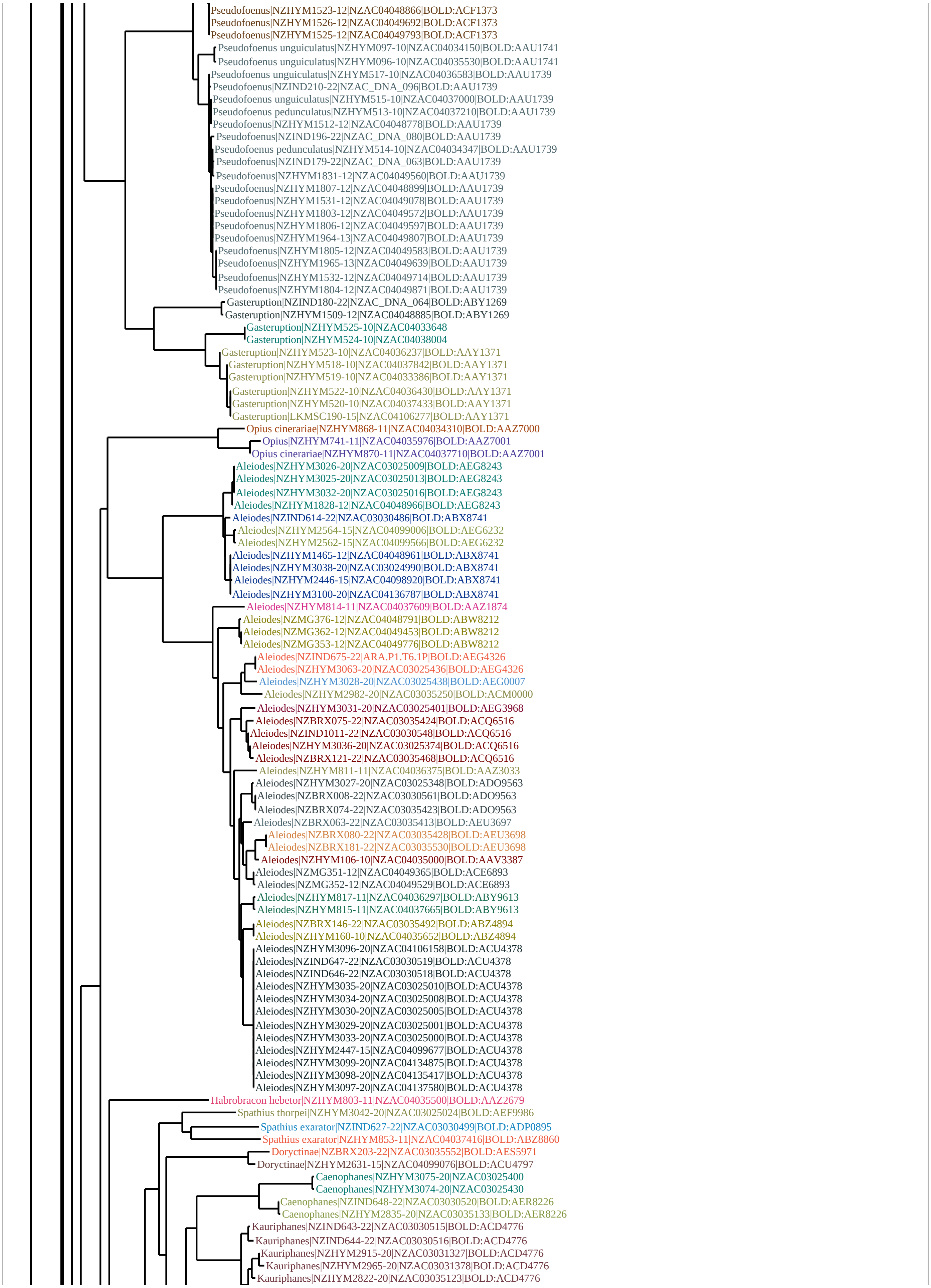
Pseudofoenus|NZHYM1523-12|NZAC04048866|BOLD:ACF1373
Pseudofoenus|NZHYM1526-12|NZAC04049692|BOLD:ACF1373
Pseudofoenus|NZHYM1525-12|NZAC04049793|BOLD:ACF1373
Pseudofoenus unguiculatus|NZHYM097-10|NZAC04034150|BOLD:AAU1741
Pseudofoenus unguiculatus|NZHYM096-10|NZAC04035530|BOLD:AAU1741
Pseudofoenus unguiculatus|NZHYM517-10|NZAC04036583|BOLD:AAU1739
Pseudofoenus|NZIND210-22|NZAC_DNA_096|BOLD:AAU1739
Pseudofoenus unguiculatus|NZHYM515-10|NZAC04037000|BOLD:AAU1739
Pseudofoenus pedunculatus|NZHYM513-10|NZAC04037210|BOLD:AAU1739
Pseudofoenus|NZHYM1512-12|NZAC04048778|BOLD:AAU1739
Pseudofoenus|NZIND196-22|NZAC_DNA_080|BOLD:AAU1739
Pseudofoenus pedunculatus|NZHYM514-10|NZAC04034347|BOLD:AAU1739
Pseudofoenus|NZIND179-22|NZAC_DNA_063|BOLD:AAU1739
Pseudofoenus|NZHYM1831-12|NZAC04049560|BOLD:AAU1739
Pseudofoenus|NZHYM1807-12|NZAC04048899|BOLD:AAU1739
Pseudofoenus|NZHYM1531-12|NZAC04049078|BOLD:AAU1739
Pseudofoenus|NZHYM1803-12|NZAC04049572|BOLD:AAU1739
Pseudofoenus|NZHYM1806-12|NZAC04049597|BOLD:AAU1739
Pseudofoenus|NZHYM1964-13|NZAC04049807|BOLD:AAU1739
Pseudofoenus|NZHYM1805-12|NZAC04049583|BOLD:AAU1739
Pseudofoenus|NZHYM1965-13|NZAC04049639|BOLD:AAU1739
Pseudofoenus|NZHYM1532-12|NZAC04049714|BOLD:AAU1739
Pseudofoenus|NZHYM1804-12|NZAC04049871|BOLD:AAU1739
Gasteruption|NZIND180-22|NZAC_DNA_064|BOLD:ABY1269
Gasteruption|NZHYM1509-12|NZAC04048885|BOLD:ABY1269
Gasteruption|NZHYM525-10|NZAC04033648
Gasteruption|NZHYM524-10|NZAC04038004
Gasteruption|NZHYM523-10|NZAC04036237|BOLD:AAY1371
Gasteruption|NZHYM518-10|NZAC04037842|BOLD:AAY1371
Gasteruption|NZHYM519-10|NZAC04033386|BOLD:AAY1371
Gasteruption|NZHYM522-10|NZAC04036430|BOLD:AAY1371
Gasteruption|NZHYM520-10|NZAC04037433|BOLD:AAY1371
Gasteruption|LKMSC190-15|NZAC04106277|BOLD:AAY1371
Opius cinerariae|NZHYM868-11|NZAC04034310|BOLD:AAZ7000
Opius|NZHYM741-11|NZAC04035976|BOLD:AAZ7001
Opius cinerariae|NZHYM870-11|NZAC04037710|BOLD:AAZ7001
Aleiodes|NZHYM3026-20|NZAC03025009|BOLD:AEG8243
Aleiodes|NZHYM3025-20|NZAC03025013|BOLD:AEG8243
Aleiodes|NZHYM3032-20|NZAC03025016|BOLD:AEG8243
Aleiodes|NZHYM1828-12|NZAC04048966|BOLD:AEG8243
Aleiodes|NZIND614-22|NZAC03030486|BOLD:ABX8741
Aleiodes|NZHYM2564-15|NZAC04099006|BOLD:AEG6232
Aleiodes|NZHYM2562-15|NZAC04099566|BOLD:AEG6232
Aleiodes|NZHYM1465-12|NZAC04048961|BOLD:ABX8741
Aleiodes|NZHYM3038-20|NZAC03024990|BOLD:ABX8741
Aleiodes|NZHYM2446-15|NZAC04098920|BOLD:ABX8741
Aleiodes|NZHYM3100-20|NZAC04136787|BOLD:ABX8741
Aleiodes|NZHYM814-11|NZAC04037609|BOLD:AAZ1874
Aleiodes|NZMG376-12|NZAC04048791|BOLD:ABW8212
Aleiodes|NZMG362-12|NZAC04049453|BOLD:ABW8212
Aleiodes|NZMG353-12|NZAC04049776|BOLD:ABW8212
Aleiodes|NZIND675-22|ARA.P1.T6.1P|BOLD:AEG4326
Aleiodes|NZHYM3063-20|NZAC03025436|BOLD:AEG4326
Aleiodes|NZHYM3028-20|NZAC03025438|BOLD:AEG0007
Aleiodes|NZHYM2982-20|NZAC03035250|BOLD:ACM0000
Aleiodes|NZHYM3031-20|NZAC03025401|BOLD:AEG3968
Aleiodes|NZBRX075-22|NZAC03035424|BOLD:ACQ6516
Aleiodes|NZIND1011-22|NZAC03030548|BOLD:ACQ6516
Aleiodes|NZHYM3036-20|NZAC03025374|BOLD:ACQ6516
Aleiodes|NZBRX121-22|NZAC03035468|BOLD:ACQ6516
Aleiodes|NZHYM811-11|NZAC04036375|BOLD:AAZ3033
Aleiodes|NZHYM3027-20|NZAC03025348|BOLD:ADO9563
Aleiodes|NZBRX008-22|NZAC03030561|BOLD:ADO9563
Aleiodes|NZBRX074-22|NZAC03035423|BOLD:ADO9563
Aleiodes|NZBRX063-22|NZAC03035413|BOLD:AEU3697
Aleiodes|NZBRX080-22|NZAC03035428|BOLD:AEU3698
Aleiodes|NZBRX181-22|NZAC03035530|BOLD:AEU3698
Aleiodes|NZHYM106-10|NZAC04035000|BOLD:AAV3387
Aleiodes|NZMG351-12|NZAC04049365|BOLD:ACE6893
Aleiodes|NZMG352-12|NZAC04049529|BOLD:ACE6893
Aleiodes|NZHYM817-11|NZAC04036297|BOLD:ABY9613
Aleiodes|NZHYM815-11|NZAC04037665|BOLD:ABY9613
Aleiodes|NZBRX146-22|NZAC03035492|BOLD:ABZ4894
Aleiodes|NZHYM160-10|NZAC04035652|BOLD:ABZ4894
Aleiodes|NZHYM3096-20|NZAC04106158|BOLD:ACU4378
Aleiodes|NZIND647-22|NZAC03030519|BOLD:ACU4378
Aleiodes|NZIND646-22|NZAC03030518|BOLD:ACU4378
Aleiodes|NZHYM3035-20|NZAC03025010|BOLD:ACU4378
Aleiodes|NZHYM3034-20|NZAC03025008|BOLD:ACU4378
Aleiodes|NZHYM3030-20|NZAC03025005|BOLD:ACU4378
Aleiodes|NZHYM3029-20|NZAC03025001|BOLD:ACU4378
Aleiodes|NZHYM3033-20|NZAC03025000|BOLD:ACU4378
Aleiodes|NZHYM2447-15|NZAC04099677|BOLD:ACU4378
Aleiodes|NZHYM3099-20|NZAC04134875|BOLD:ACU4378
Aleiodes|NZHYM3098-20|NZAC04135417|BOLD:ACU4378
Aleiodes|NZHYM3097-20|NZAC04137580|BOLD:ACU4378
Habrobracon hebetor|NZHYM803-11|NZAC04035500|BOLD:AAZ2679
Spathius thorpei|NZHYM3042-20|NZAC03025024|BOLD:AEF9986
Spathius exarator|NZIND627-22|NZAC03030499|BOLD:ADP0895
Spathius exarator|NZHYM853-11|NZAC04037416|BOLD:ABZ8860
Doryctinae|NZBRX203-22|NZAC03035552|BOLD:AES5971
Doryctinae|NZHYM2631-15|NZAC04099076|BOLD:ACU4797
Caenophanes|NZHYM3075-20|NZAC03025400
Caenophanes|NZHYM3074-20|NZAC03025430
Caenophanes|NZIND648-22|NZAC03030520|BOLD:AER8226
Caenophanes|NZHYM2835-20|NZAC03035133|BOLD:AER8226
Kauriphanes|NZIND643-22|NZAC03030515|BOLD:ACD4776
Kauriphanes|NZIND644-22|NZAC03030516|BOLD:ACD4776
Kauriphanes|NZHYM2915-20|NZAC03031327|BOLD:ACD4776
Kauriphanes|NZHYM2965-20|NZAC03031378|BOLD:ACD4776
Kauriphanes|NZHYM2822-20|NZAC03035123|BOLD:ACD4776

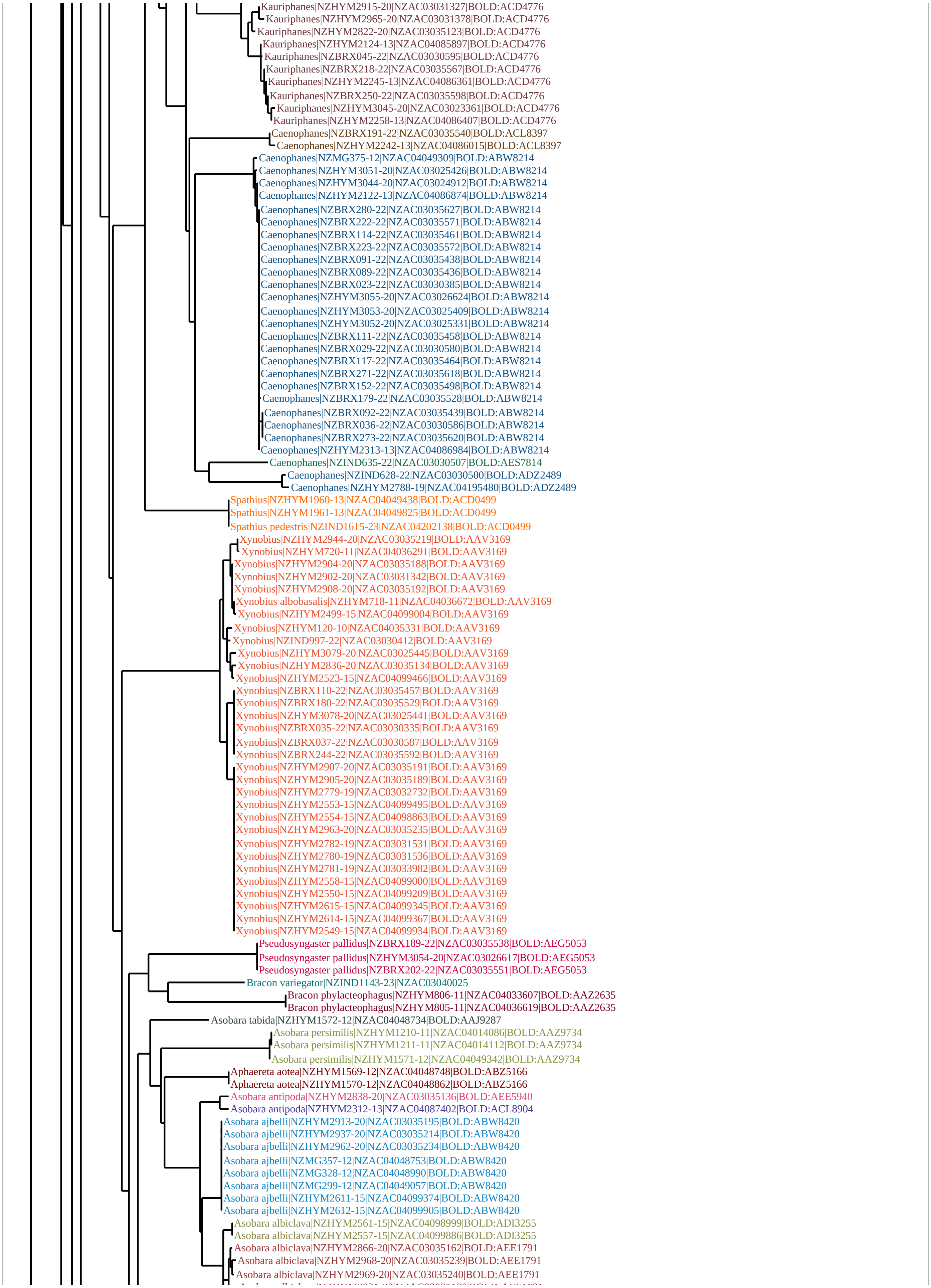
Kauriphanes|NZHYM2915-20|NZAC03031327|BOLD:ACD4776
Kauriphanes|NZHYM2965-20|NZAC03031378|BOLD:ACD4776
Kauriphanes|NZHYM2822-20|NZAC03035123|BOLD:ACD4776
Kauriphanes|NZHYM2124-13|NZAC04085897|BOLD:ACD4776
Kauriphanes|NZBRX045-22|NZAC03030595|BOLD:ACD4776
Kauriphanes|NZBRX218-22|NZAC03035567|BOLD:ACD4776
Kauriphanes|NZHYM2245-13|NZAC04086361|BOLD:ACD4776
Kauriphanes|NZBRX250-22|NZAC03035598|BOLD:ACD4776
Kauriphanes|NZHYM3045-20|NZAC03023361|BOLD:ACD4776
Kauriphanes|NZHYM2258-13|NZAC04086407|BOLD:ACD4776
Caenophanes|NZBRX191-22|NZAC03035540|BOLD:ACL8397
Caenophanes|NZHYM2242-13|NZAC04086015|BOLD:ACL8397
Caenophanes|NZMG375-12|NZAC04049309|BOLD:ABW8214
Caenophanes|NZHYM3051-20|NZAC03025426|BOLD:ABW8214
Caenophanes|NZHYM3044-20|NZAC03024912|BOLD:ABW8214
Caenophanes|NZHYM2122-13|NZAC04086874|BOLD:ABW8214
Caenophanes|NZBRX280-22|NZAC03035627|BOLD:ABW8214
Caenophanes|NZBRX222-22|NZAC03035571|BOLD:ABW8214
Caenophanes|NZBRX114-22|NZAC03035461|BOLD:ABW8214
Caenophanes|NZBRX223-22|NZAC03035572|BOLD:ABW8214
Caenophanes|NZBRX091-22|NZAC03035438|BOLD:ABW8214
Caenophanes|NZBRX089-22|NZAC03035436|BOLD:ABW8214
Caenophanes|NZBRX023-22|NZAC03030385|BOLD:ABW8214
Caenophanes|NZHYM3055-20|NZAC03026624|BOLD:ABW8214
Caenophanes|NZHYM3053-20|NZAC03025409|BOLD:ABW8214
Caenophanes|NZHYM3052-20|NZAC03025331|BOLD:ABW8214
Caenophanes|NZBRX111-22|NZAC03035458|BOLD:ABW8214
Caenophanes|NZBRX029-22|NZAC03030580|BOLD:ABW8214
Caenophanes|NZBRX117-22|NZAC03035464|BOLD:ABW8214
Caenophanes|NZBRX271-22|NZAC03035618|BOLD:ABW8214
Caenophanes|NZBRX152-22|NZAC03035498|BOLD:ABW8214
Caenophanes|NZBRX179-22|NZAC03035528|BOLD:ABW8214
Caenophanes|NZBRX092-22|NZAC03035439|BOLD:ABW8214
Caenophanes|NZBRX036-22|NZAC03030586|BOLD:ABW8214
Caenophanes|NZBRX273-22|NZAC03035620|BOLD:ABW8214
Caenophanes|NZHYM2313-13|NZAC04086984|BOLD:ABW8214
Caenophanes|NZIND635-22|NZAC03030507|BOLD:AES7814
Caenophanes|NZIND628-22|NZAC03030500|BOLD:ADZ2489
Caenophanes|NZHYM2788-19|NZAC04195480|BOLD:ADZ2489
Spathius|NZHYM1960-13|NZAC04049438|BOLD:ACD0499
Spathius|NZHYM1961-13|NZAC04049825|BOLD:ACD0499
Spathius pedestris|NZIND1615-23|NZAC04202138|BOLD:ACD0499
Xynobius|NZHYM2944-20|NZAC03035219|BOLD:AAV3169
Xynobius|NZHYM720-11|NZAC04036291|BOLD:AAV3169
Xynobius|NZHYM2904-20|NZAC03035188|BOLD:AAV3169
Xynobius|NZHYM2902-20|NZAC03031342|BOLD:AAV3169
Xynobius|NZHYM2908-20|NZAC03035192|BOLD:AAV3169
Xynobius albobasalis|NZHYM718-11|NZAC04036672|BOLD:AAV3169
Xynobius|NZHYM2499-15|NZAC04099004|BOLD:AAV3169
Xynobius|NZHYM120-10|NZAC04035331|BOLD:AAV3169
Xynobius|NZIND997-22|NZAC03030412|BOLD:AAV3169
Xynobius|NZHYM3079-20|NZAC03025445|BOLD:AAV3169
Xynobius|NZHYM2836-20|NZAC03035134|BOLD:AAV3169
Xynobius|NZHYM2523-15|NZAC04099466|BOLD:AAV3169
Xynobius|NZBRX110-22|NZAC03035457|BOLD:AAV3169
Xynobius|NZBRX180-22|NZAC03035529|BOLD:AAV3169
Xynobius|NZHYM3078-20|NZAC03025441|BOLD:AAV3169
Xynobius|NZBRX035-22|NZAC03030335|BOLD:AAV3169
Xynobius|NZBRX037-22|NZAC03030587|BOLD:AAV3169
Xynobius|NZBRX244-22|NZAC03035592|BOLD:AAV3169
Xynobius|NZHYM2907-20|NZAC03035191|BOLD:AAV3169
Xynobius|NZHYM2905-20|NZAC03035189|BOLD:AAV3169
Xynobius|NZHYM2779-19|NZAC03032732|BOLD:AAV3169
Xynobius|NZHYM2553-15|NZAC04099495|BOLD:AAV3169
Xynobius|NZHYM2554-15|NZAC04098863|BOLD:AAV3169
Xynobius|NZHYM2963-20|NZAC03035235|BOLD:AAV3169
Xynobius|NZHYM2782-19|NZAC03031531|BOLD:AAV3169
Xynobius|NZHYM2780-19|NZAC03031536|BOLD:AAV3169
Xynobius|NZHYM2781-19|NZAC03033982|BOLD:AAV3169
Xynobius|NZHYM2558-15|NZAC04099000|BOLD:AAV3169
Xynobius|NZHYM2550-15|NZAC04099209|BOLD:AAV3169
Xynobius|NZHYM2615-15|NZAC04099345|BOLD:AAV3169
Xynobius|NZHYM2614-15|NZAC04099367|BOLD:AAV3169
Xynobius|NZHYM2549-15|NZAC04099934|BOLD:AAV3169
Pseudosyngaster pallidus|NZBRX189-22|NZAC03035538|BOLD:AEG5053
Pseudosyngaster pallidus|NZHYM3054-20|NZAC03026617|BOLD:AEG5053
Pseudosyngaster pallidus|NZBRX202-22|NZAC03035551|BOLD:AEG5053
Bracon variegator|NZIND1143-23|NZAC03040025
Bracon phylacteophagus|NZHYM806-11|NZAC04033607|BOLD:AAZ2635
Bracon phylacteophagus|NZHYM805-11|NZAC04036619|BOLD:AAZ2635
Asobara tabida|NZHYM1572-12|NZAC04048734|BOLD:AAJ9287
Asobara persimilis|NZHYM1210-11|NZAC04014086|BOLD:AAZ9734
Asobara persimilis|NZHYM1211-11|NZAC04014112|BOLD:AAZ9734
Asobara persimilis|NZHYM1571-12|NZAC04049342|BOLD:AAZ9734
Aphaereta aotea|NZHYM1569-12|NZAC04048748|BOLD:ABZ5166
Aphaereta aotea|NZHYM1570-12|NZAC04048862|BOLD:ABZ5166
Asobara antipoda|NZHYM2838-20|NZAC03035136|BOLD:AEE5940
Asobara antipoda|NZHYM2312-13|NZAC04087402|BOLD:ACL8904
Asobara ajbelli|NZHYM2913-20|NZAC03035195|BOLD:ABW8420
Asobara ajbelli|NZHYM2937-20|NZAC03035214|BOLD:ABW8420
Asobara ajbelli|NZHYM2962-20|NZAC03035234|BOLD:ABW8420
Asobara ajbelli|NZMG357-12|NZAC04048753|BOLD:ABW8420
Asobara ajbelli|NZMG328-12|NZAC04048990|BOLD:ABW8420
Asobara ajbelli|NZMG299-12|NZAC04049057|BOLD:ABW8420
Asobara ajbelli|NZHYM2611-15|NZAC04099374|BOLD:ABW8420
Asobara ajbelli|NZHYM2612-15|NZAC04099905|BOLD:ABW8420
Asobara albiclava|NZHYM2561-15|NZAC04098999|BOLD:ADI3255
Asobara albiclava|NZHYM2557-15|NZAC04099886|BOLD:ADI3255
Asobara albiclava|NZHYM2866-20|NZAC03035162|BOLD:AEE1791
Asobara albiclava|NZHYM2968-20|NZAC03035239|BOLD:AEE1791
Asobara albiclava|NZHYM2969-20|NZAC03035240|BOLD:AEE1791

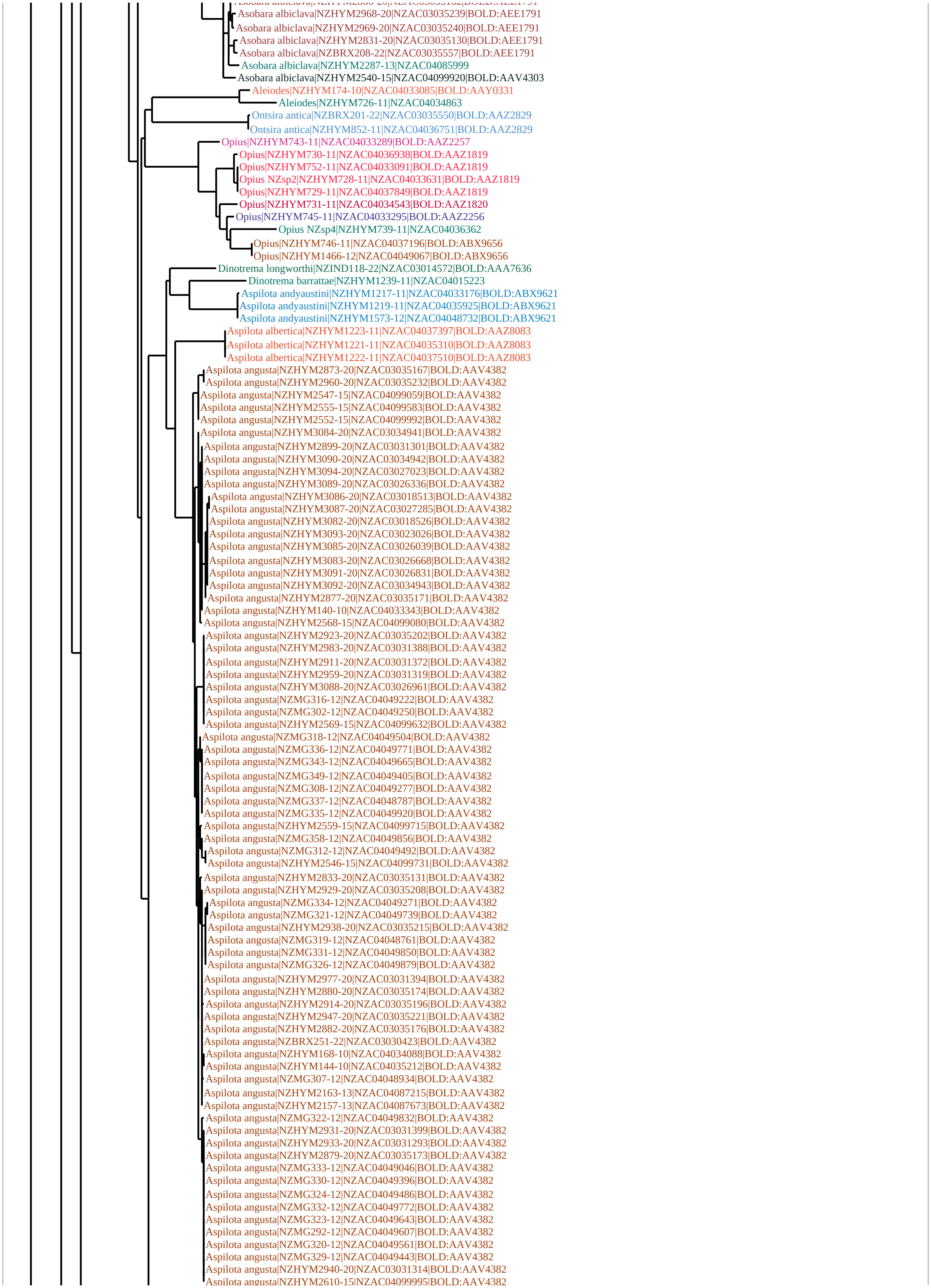
Asobara albiclava|NZHYM2968-20|NZAC03035239|BOLD:AEE1791
Asobara albiclava|NZHYM2969-20|NZAC03035240|BOLD:AEE1791
Asobara albiclava|NZHYM2831-20|NZAC03035130|BOLD:AEE1791
Asobara albiclava|NZBRX208-22|NZAC03035557|BOLD:AEE1791
Asobara albiclava|NZHYM2287-13|NZAC04085999
Asobara albiclava|NZHYM2540-15|NZAC04099920|BOLD:AAV4303
Aleiodes|NZHYM174-10|NZAC04033085|BOLD:AAY0331
Aleiodes|NZHYM726-11|NZAC04034863
Ontsira antica|NZBRX201-22|NZAC03035550|BOLD:AAZ2829
Ontsira antica|NZHYM852-11|NZAC04036751|BOLD:AAZ2829
Opius|NZHYM743-11|NZAC04033289|BOLD:AAZ2257
Opius|NZHYM730-11|NZAC04036938|BOLD:AAZ1819
Opius|NZHYM752-11|NZAC04033091|BOLD:AAZ1819
Opius NZsp2|NZHYM728-11|NZAC04033631|BOLD:AAZ1819
Opius|NZHYM729-11|NZAC04037849|BOLD:AAZ1819
Opius|NZHYM731-11|NZAC04034543|BOLD:AAZ1820
Opius|NZHYM745-11|NZAC04033295|BOLD:AAZ2256
Opius NZsp4|NZHYM739-11|NZAC04036362
Opius|NZHYM746-11|NZAC04037196|BOLD:ABX9656
Opius|NZHYM1466-12|NZAC04049067|BOLD:ABX9656
Dinotrema longworthi|NZIND118-22|NZAC03014572|BOLD:AAA7636
Dinotrema barrattae|NZHYM1239-11|NZAC04015223
Aspilota andyaustini|NZHYM1217-11|NZAC04033176|BOLD:ABX9621
Aspilota andyaustini|NZHYM1219-11|NZAC04035925|BOLD:ABX9621
Aspilota andyaustini|NZHYM1573-12|NZAC04048732|BOLD:ABX9621
Aspilota albertica|NZHYM1223-11|NZAC04037397|BOLD:AAZ8083
Aspilota albertica|NZHYM1221-11|NZAC04035310|BOLD:AAZ8083
Aspilota albertica|NZHYM1222-11|NZAC04037510|BOLD:AAZ8083
Aspilota angusta|NZHYM2873-20|NZAC03035167|BOLD:AAV4382
Aspilota angusta|NZHYM2960-20|NZAC03035232|BOLD:AAV4382
Aspilota angusta|NZHYM2547-15|NZAC04099059|BOLD:AAV4382
Aspilota angusta|NZHYM2555-15|NZAC04099583|BOLD:AAV4382
Aspilota angusta|NZHYM2552-15|NZAC04099992|BOLD:AAV4382
Aspilota angusta|NZHYM3084-20|NZAC03034941|BOLD:AAV4382
Aspilota angusta|NZHYM2899-20|NZAC03031301|BOLD:AAV4382
Aspilota angusta|NZHYM3090-20|NZAC03034942|BOLD:AAV4382
Aspilota angusta|NZHYM3094-20|NZAC03027023|BOLD:AAV4382
Aspilota angusta|NZHYM3089-20|NZAC03026336|BOLD:AAV4382
Aspilota angusta|NZHYM3086-20|NZAC03018513|BOLD:AAV4382
Aspilota angusta|NZHYM3087-20|NZAC03027285|BOLD:AAV4382
Aspilota angusta|NZHYM3082-20|NZAC03018526|BOLD:AAV4382
Aspilota angusta|NZHYM3093-20|NZAC03023026|BOLD:AAV4382
Aspilota angusta|NZHYM3085-20|NZAC03026039|BOLD:AAV4382
Aspilota angusta|NZHYM3083-20|NZAC03026668|BOLD:AAV4382
Aspilota angusta|NZHYM3091-20|NZAC03026831|BOLD:AAV4382
Aspilota angusta|NZHYM3092-20|NZAC03034943|BOLD:AAV4382
Aspilota angusta|NZHYM2877-20|NZAC03035171|BOLD:AAV4382
Aspilota angusta|NZHYM140-10|NZAC04033343|BOLD:AAV4382
Aspilota angusta|NZHYM2568-15|NZAC04099080|BOLD:AAV4382
Aspilota angusta|NZHYM2923-20|NZAC03035202|BOLD:AAV4382
Aspilota angusta|NZHYM2983-20|NZAC03031388|BOLD:AAV4382
Aspilota angusta|NZHYM2911-20|NZAC03031372|BOLD:AAV4382
Aspilota angusta|NZHYM2959-20|NZAC03031319|BOLD:AAV4382
Aspilota angusta|NZHYM3088-20|NZAC03026961|BOLD:AAV4382
Aspilota angusta|NZMG316-12|NZAC04049222|BOLD:AAV4382
Aspilota angusta|NZMG302-12|NZAC04049250|BOLD:AAV4382
Aspilota angusta|NZHYM2569-15|NZAC04099632|BOLD:AAV4382
Aspilota angusta|NZMG318-12|NZAC04049504|BOLD:AAV4382
Aspilota angusta|NZMG336-12|NZAC04049771|BOLD:AAV4382
Aspilota angusta|NZMG343-12|NZAC04049665|BOLD:AAV4382
Aspilota angusta|NZMG349-12|NZAC04049405|BOLD:AAV4382
Aspilota angusta|NZMG308-12|NZAC04049277|BOLD:AAV4382
Aspilota angusta|NZMG337-12|NZAC04048787|BOLD:AAV4382
Aspilota angusta|NZMG335-12|NZAC04049920|BOLD:AAV4382
Aspilota angusta|NZHYM2559-15|NZAC04099715|BOLD:AAV4382
Aspilota angusta|NZMG358-12|NZAC04049856|BOLD:AAV4382
Aspilota angusta|NZMG312-12|NZAC04049492|BOLD:AAV4382
Aspilota angusta|NZHYM2546-15|NZAC04099731|BOLD:AAV4382
Aspilota angusta|NZHYM2833-20|NZAC03035131|BOLD:AAV4382
Aspilota angusta|NZHYM2929-20|NZAC03035208|BOLD:AAV4382
Aspilota angusta|NZMG334-12|NZAC04049271|BOLD:AAV4382
Aspilota angusta|NZMG321-12|NZAC04049739|BOLD:AAV4382
Aspilota angusta|NZHYM2938-20|NZAC03035215|BOLD:AAV4382
Aspilota angusta|NZMG319-12|NZAC04048761|BOLD:AAV4382
Aspilota angusta|NZMG331-12|NZAC04049850|BOLD:AAV4382
Aspilota angusta|NZMG326-12|NZAC04049879|BOLD:AAV4382
Aspilota angusta|NZHYM2977-20|NZAC03031394|BOLD:AAV4382
Aspilota angusta|NZHYM2880-20|NZAC03035174|BOLD:AAV4382
Aspilota angusta|NZHYM2914-20|NZAC03035196|BOLD:AAV4382
Aspilota angusta|NZHYM2947-20|NZAC03035221|BOLD:AAV4382
Aspilota angusta|NZHYM2882-20|NZAC03035176|BOLD:AAV4382
Aspilota angusta|NZBRX251-22|NZAC03030423|BOLD:AAV4382
Aspilota angusta|NZHYM168-10|NZAC04034088|BOLD:AAV4382
Aspilota angusta|NZHYM144-10|NZAC04035212|BOLD:AAV4382
Aspilota angusta|NZMG307-12|NZAC04048934|BOLD:AAV4382
Aspilota angusta|NZHYM2163-13|NZAC04087215|BOLD:AAV4382
Aspilota angusta|NZHYM2157-13|NZAC04087673|BOLD:AAV4382
Aspilota angusta|NZMG322-12|NZAC04049832|BOLD:AAV4382
Aspilota angusta|NZHYM2931-20|NZAC03031399|BOLD:AAV4382
Aspilota angusta|NZHYM2933-20|NZAC03031293|BOLD:AAV4382
Aspilota angusta|NZHYM2879-20|NZAC03035173|BOLD:AAV4382
Aspilota angusta|NZMG333-12|NZAC04049046|BOLD:AAV4382
Aspilota angusta|NZMG330-12|NZAC04049396|BOLD:AAV4382
Aspilota angusta|NZMG324-12|NZAC04049486|BOLD:AAV4382
Aspilota angusta|NZMG332-12|NZAC04049772|BOLD:AAV4382
Aspilota angusta|NZMG323-12|NZAC04049643|BOLD:AAV4382
Aspilota angusta|NZMG292-12|NZAC04049607|BOLD:AAV4382
Aspilota angusta|NZMG320-12|NZAC04049561|BOLD:AAV4382
Aspilota angusta|NZMG329-12|NZAC04049443|BOLD:AAV4382
Aspilota angusta|NZHYM2940-20|NZAC03031314|BOLD:AAV4382
Aspilota angusta|NZHYM2610-15|NZAC04099995|BOLD:AAV4382

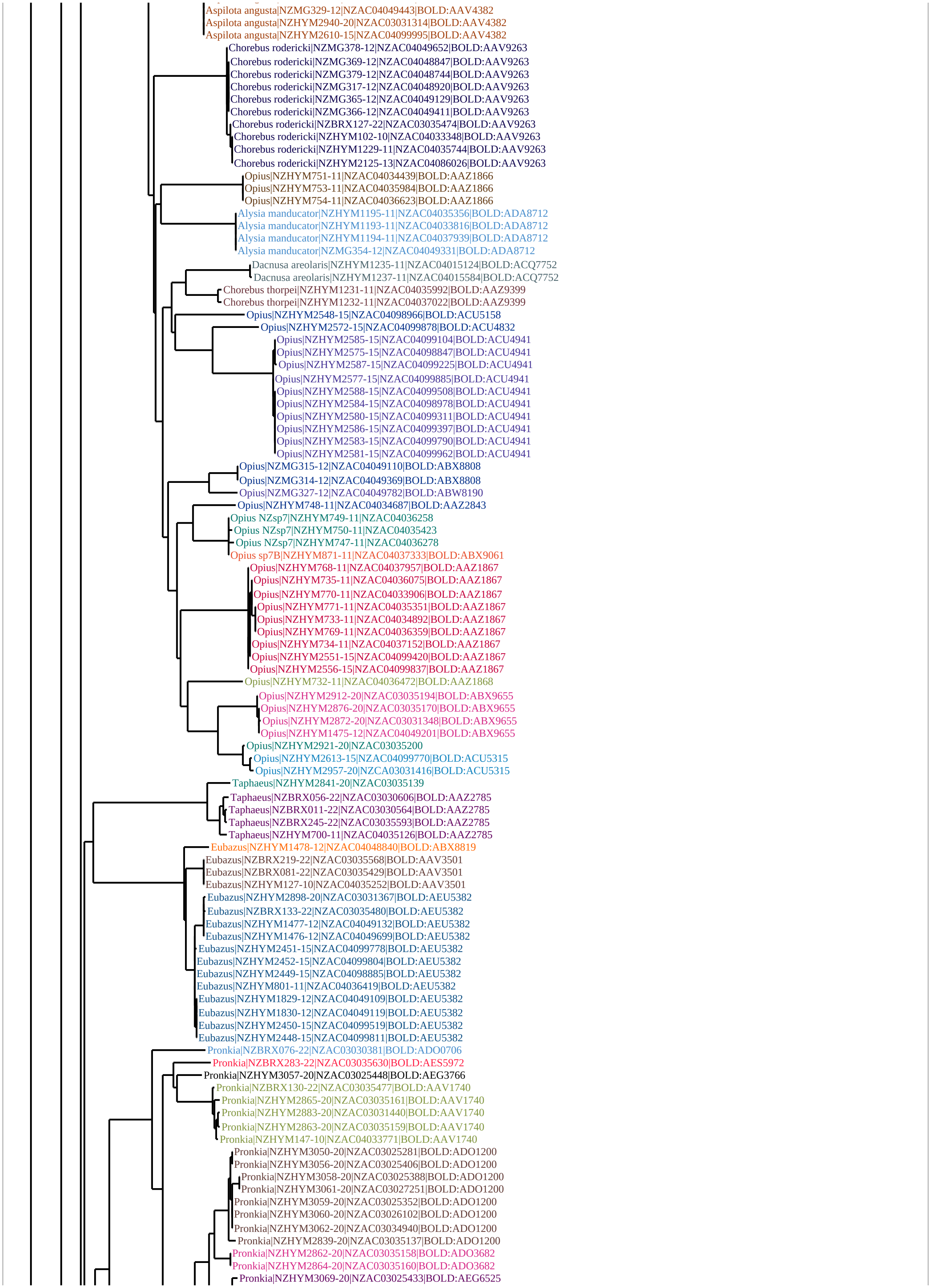

Aspilota angusta|NZMG329-12|NZAC04049443|BOLD:AAV4382
Aspilota angusta|NZHYM2940-20|NZAC03031314|BOLD:AAV4382
Aspilota angusta|NZHYM2610-15|NZAC04099995|BOLD:AAV4382
Chorebus rodericki|NZMG378-12|NZAC04049652|BOLD:AAV9263
Chorebus rodericki|NZMG369-12|NZAC04048847|BOLD:AAV9263
Chorebus rodericki|NZMG379-12|NZAC04048744|BOLD:AAV9263
Chorebus rodericki|NZMG317-12|NZAC04048920|BOLD:AAV9263
Chorebus rodericki|NZMG365-12|NZAC04049129|BOLD:AAV9263
Chorebus rodericki|NZMG366-12|NZAC04049411|BOLD:AAV9263
Chorebus rodericki|NZBRX127-22|NZAC03035474|BOLD:AAV9263
Chorebus rodericki|NZHYM102-10|NZAC04033348|BOLD:AAV9263
Chorebus rodericki|NZHYM1229-11|NZAC04035744|BOLD:AAV9263
Chorebus rodericki|NZHYM2125-13|NZAC04086026|BOLD:AAV9263
Opius|NZHYM751-11|NZAC04034439|BOLD:AAZ1866
Opius|NZHYM753-11|NZAC04035984|BOLD:AAZ1866
Opius|NZHYM754-11|NZAC04036623|BOLD:AAZ1866
Alysia manducator|NZHYM1195-11|NZAC04035356|BOLD:ADA8712
Alysia manducator|NZHYM1193-11|NZAC04033816|BOLD:ADA8712
Alysia manducator|NZHYM1194-11|NZAC04037939|BOLD:ADA8712
Alysia manducator|NZMG354-12|NZAC04049331|BOLD:ADA8712
Dacnusa areolaris|NZHYM1235-11|NZAC04015124|BOLD:ACQ7752
Dacnusa areolaris|NZHYM1237-11|NZAC04015584|BOLD:ACQ7752
Chorebus thorpei|NZHYM1231-11|NZAC04035992|BOLD:AAZ9399
Chorebus thorpei|NZHYM1232-11|NZAC04037022|BOLD:AAZ9399
Opius|NZHYM2548-15|NZAC04098966|BOLD:ACU5158
Opius|NZHYM2572-15|NZAC04099878|BOLD:ACU4832
Opius|NZHYM2585-15|NZAC04099104|BOLD:ACU4941
Opius|NZHYM2575-15|NZAC04098847|BOLD:ACU4941
Opius|NZHYM2587-15|NZAC04099225|BOLD:ACU4941
Opius|NZHYM2577-15|NZAC04099885|BOLD:ACU4941
Opius|NZHYM2588-15|NZAC04099508|BOLD:ACU4941
Opius|NZHYM2584-15|NZAC04098978|BOLD:ACU4941
Opius|NZHYM2580-15|NZAC04099311|BOLD:ACU4941
Opius|NZHYM2586-15|NZAC04099397|BOLD:ACU4941
Opius|NZHYM2583-15|NZAC04099790|BOLD:ACU4941
Opius|NZHYM2581-15|NZAC04099962|BOLD:ACU4941
Opius|NZMG315-12|NZAC04049110|BOLD:ABX8808
Opius|NZMG314-12|NZAC04049369|BOLD:ABX8808
Opius|NZMG327-12|NZAC04049782|BOLD:ABW8190
Opius|NZHYM748-11|NZAC04034687|BOLD:AAZ2843
Opius NZsp7|NZHYM749-11|NZAC04036258
Opius NZsp7|NZHYM750-11|NZAC04035423
Opius NZsp7|NZHYM747-11|NZAC04036278
Opius sp7B|NZHYM871-11|NZAC04037333|BOLD:ABX9061
Opius|NZHYM768-11|NZAC04037957|BOLD:AAZ1867
Opius|NZHYM735-11|NZAC04036075|BOLD:AAZ1867
Opius|NZHYM770-11|NZAC04033906|BOLD:AAZ1867
Opius|NZHYM771-11|NZAC04035351|BOLD:AAZ1867
Opius|NZHYM733-11|NZAC04034892|BOLD:AAZ1867
Opius|NZHYM769-11|NZAC04036359|BOLD:AAZ1867
Opius|NZHYM734-11|NZAC04037152|BOLD:AAZ1867
Opius|NZHYM2551-15|NZAC04099420|BOLD:AAZ1867
Opius|NZHYM2556-15|NZAC04099837|BOLD:AAZ1867
Opius|NZHYM732-11|NZAC04036472|BOLD:AAZ1868
Opius|NZHYM2912-20|NZAC03035194|BOLD:ABX9655
Opius|NZHYM2876-20|NZAC03035170|BOLD:ABX9655
Opius|NZHYM2872-20|NZAC03031348|BOLD:ABX9655
Opius|NZHYM1475-12|NZAC04049201|BOLD:ABX9655
Opius|NZHYM2921-20|NZAC03035200
Opius|NZHYM2613-15|NZAC04099770|BOLD:ACU5315
Opius|NZHYM2957-20|NZCA03031416|BOLD:ACU5315
Taphaeus|NZHYM2841-20|NZAC03035139
Taphaeus|NZBRX056-22|NZAC03030606|BOLD:AAZ2785
Taphaeus|NZBRX011-22|NZAC03030564|BOLD:AAZ2785
Taphaeus|NZBRX245-22|NZAC03035593|BOLD:AAZ2785
Taphaeus|NZHYM700-11|NZAC04035126|BOLD:AAZ2785
Eubazus|NZHYM1478-12|NZAC04048840|BOLD:ABX8819
Eubazus|NZBRX219-22|NZAC03035568|BOLD:AAV3501
Eubazus|NZBRX081-22|NZAC03035429|BOLD:AAV3501
Eubazus|NZHYM127-10|NZAC04035252|BOLD:AAV3501
Eubazus|NZHYM2898-20|NZAC03031367|BOLD:AEU5382
Eubazus|NZBRX133-22|NZAC03035480|BOLD:AEU5382
Eubazus|NZHYM1477-12|NZAC04049132|BOLD:AEU5382
Eubazus|NZHYM1476-12|NZAC04049699|BOLD:AEU5382
Eubazus|NZHYM2451-15|NZAC04099778|BOLD:AEU5382
Eubazus|NZHYM2452-15|NZAC04099804|BOLD:AEU5382
Eubazus|NZHYM2449-15|NZAC04098885|BOLD:AEU5382
Eubazus|NZHYM801-11|NZAC04036419|BOLD:AEU5382
Eubazus|NZHYM1829-12|NZAC04049109|BOLD:AEU5382
Eubazus|NZHYM1830-12|NZAC04049119|BOLD:AEU5382
Eubazus|NZHYM2450-15|NZAC04099519|BOLD:AEU5382
Eubazus|NZHYM2448-15|NZAC04099811|BOLD:AEU5382
Pronkia|NZBRX076-22|NZAC03030381|BOLD:ADO0706
Pronkia|NZBRX283-22|NZAC03035630|BOLD:AES5972
Pronkia|NZHYM3057-20|NZAC03025448|BOLD:AEG3766
Pronkia|NZBRX130-22|NZAC03035477|BOLD:AAV1740
Pronkia|NZHYM2865-20|NZAC03035161|BOLD:AAV1740
Pronkia|NZHYM2883-20|NZAC03031440|BOLD:AAV1740
Pronkia|NZHYM2863-20|NZAC03035159|BOLD:AAV1740
Pronkia|NZHYM147-10|NZAC04033771|BOLD:AAV1740
Pronkia|NZHYM3050-20|NZAC03025281|BOLD:ADO1200
Pronkia|NZHYM3056-20|NZAC03025406|BOLD:ADO1200
Pronkia|NZHYM3058-20|NZAC03025388|BOLD:ADO1200
Pronkia|NZHYM3061-20|NZAC03027251|BOLD:ADO1200
Pronkia|NZHYM3059-20|NZAC03025352|BOLD:ADO1200
Pronkia|NZHYM3060-20|NZAC03026102|BOLD:ADO1200
Pronkia|NZHYM3062-20|NZAC03034940|BOLD:ADO1200
Pronkia|NZHYM2839-20|NZAC03035137|BOLD:ADO1200
Pronkia|NZHYM2862-20|NZAC03035158|BOLD:ADO3682
Pronkia|NZHYM2864-20|NZAC03035160|BOLD:ADO3682
Pronkia|NZHYM3069-20|NZAC03025433|BOLD:AEG6525

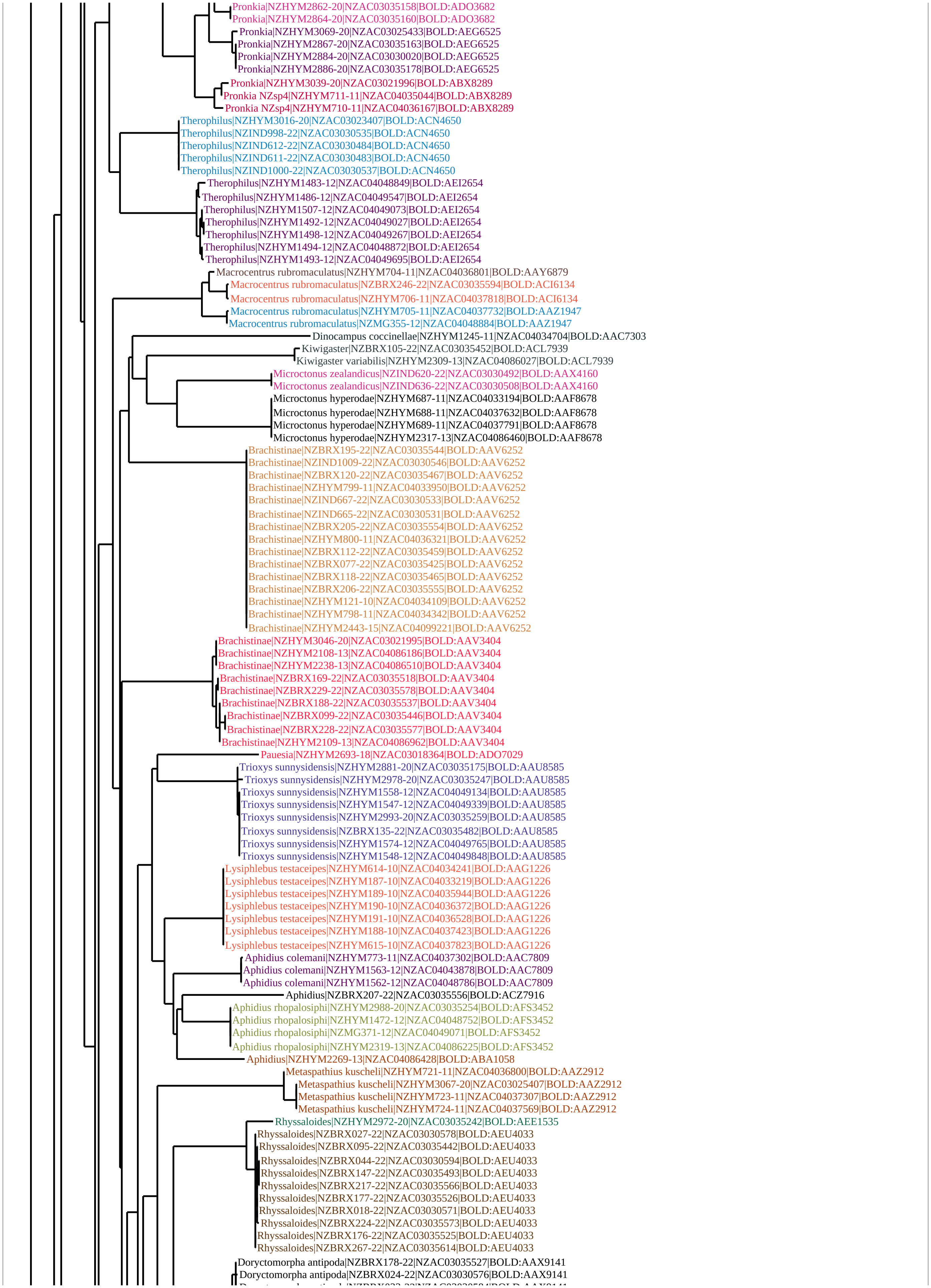
Pronkia|NZHYM2862-20|NZAC03035158|BOLD:ADO3682
Pronkia|NZHYM2864-20|NZAC03035160|BOLD:ADO3682
Pronkia|NZHYM3069-20|NZAC03025433|BOLD:AEG6525
Pronkia|NZHYM2867-20|NZAC03035163|BOLD:AEG6525
Pronkia|NZHYM2884-20|NZAC03030020|BOLD:AEG6525
Pronkia|NZHYM2886-20|NZAC03035178|BOLD:AEG6525
Pronkia|NZHYM3039-20|NZAC03021996|BOLD:ABX8289
Pronkia NZsp4|NZHYM711-11|NZAC04035044|BOLD:ABX8289
Pronkia NZsp4|NZHYM710-11|NZAC04036167|BOLD:ABX8289
Therophilus|NZHYM3016-20|NZAC03023407|BOLD:ACN4650
Therophilus|NZIND998-22|NZAC03030535|BOLD:ACN4650
Therophilus|NZIND612-22|NZAC03030484|BOLD:ACN4650
Therophilus|NZIND611-22|NZAC03030483|BOLD:ACN4650
Therophilus|NZIND1000-22|NZAC03030537|BOLD:ACN4650
Therophilus|NZHYM1483-12|NZAC04048849|BOLD:AEI2654
Therophilus|NZHYM1486-12|NZAC04049547|BOLD:AEI2654
Therophilus|NZHYM1507-12|NZAC04049073|BOLD:AEI2654
Therophilus|NZHYM1492-12|NZAC04049027|BOLD:AEI2654
Therophilus|NZHYM1498-12|NZAC04049267|BOLD:AEI2654
Therophilus|NZHYM1494-12|NZAC04048872|BOLD:AEI2654
Therophilus|NZHYM1493-12|NZAC04049695|BOLD:AEI2654
Macrocentrus rubromaculatus|NZHYM704-11|NZAC04036801|BOLD:AAY6879
Macrocentrus rubromaculatus|NZBRX246-22|NZAC03035594|BOLD:ACI6134
Macrocentrus rubromaculatus|NZHYM706-11|NZAC04037818|BOLD:ACI6134
Macrocentrus rubromaculatus|NZHYM705-11|NZAC04037732|BOLD:AAZ1947
Macrocentrus rubromaculatus|NZMG355-12|NZAC04048884|BOLD:AAZ1947
Dinocampus coccinellae|NZHYM1245-11|NZAC04034704|BOLD:AAC7303
Kiwigaster|NZBRX105-22|NZAC03035452|BOLD:ACL7939
Kiwigaster variabilis|NZHYM2309-13|NZAC04086027|BOLD:ACL7939
Microctonus zealandicus|NZIND620-22|NZAC03030492|BOLD:AAX4160
Microctonus zealandicus|NZIND636-22|NZAC03030508|BOLD:AAX4160
Microctonus hyperodae|NZHYM687-11|NZAC04033194|BOLD:AAF8678
Microctonus hyperodae|NZHYM688-11|NZAC04037632|BOLD:AAF8678
Microctonus hyperodae|NZHYM689-11|NZAC04037791|BOLD:AAF8678
Microctonus hyperodae|NZHYM2317-13|NZAC04086460|BOLD:AAF8678
Brachistinae|NZBRX195-22|NZAC03035544|BOLD:AAV6252
Brachistinae|NZIND1009-22|NZAC03030546|BOLD:AAV6252
Brachistinae|NZBRX120-22|NZAC03035467|BOLD:AAV6252
Brachistinae|NZHYM799-11|NZAC04033950|BOLD:AAV6252
Brachistinae|NZIND667-22|NZAC03030533|BOLD:AAV6252
Brachistinae|NZIND665-22|NZAC03030531|BOLD:AAV6252
Brachistinae|NZBRX205-22|NZAC03035554|BOLD:AAV6252
Brachistinae|NZHYM800-11|NZAC04036321|BOLD:AAV6252
Brachistinae|NZBRX112-22|NZAC03035459|BOLD:AAV6252
Brachistinae|NZBRX077-22|NZAC03035425|BOLD:AAV6252
Brachistinae|NZBRX118-22|NZAC03035465|BOLD:AAV6252
Brachistinae|NZBRX206-22|NZAC03035555|BOLD:AAV6252
Brachistinae|NZHYM121-10|NZAC04034109|BOLD:AAV6252
Brachistinae|NZHYM798-11|NZAC04034342|BOLD:AAV6252
Brachistinae|NZHYM2443-15|NZAC04099221|BOLD:AAV6252
Brachistinae|NZHYM3046-20|NZAC03021995|BOLD:AAV3404
Brachistinae|NZHYM2108-13|NZAC04086186|BOLD:AAV3404
Brachistinae|NZHYM2238-13|NZAC04086510|BOLD:AAV3404
Brachistinae|NZBRX169-22|NZAC03035518|BOLD:AAV3404
Brachistinae|NZBRX229-22|NZAC03035578|BOLD:AAV3404
Brachistinae|NZBRX188-22|NZAC03035537|BOLD:AAV3404
Brachistinae|NZBRX099-22|NZAC03035446|BOLD:AAV3404
Brachistinae|NZBRX228-22|NZAC03035577|BOLD:AAV3404
Brachistinae|NZHYM2109-13|NZAC04086962|BOLD:AAV3404
Pauesia|NZHYM2693-18|NZAC03018364|BOLD:ADO7029
Trioxys sunnysidensis|NZHYM2881-20|NZAC03035175|BOLD:AAU8585
Trioxys sunnysidensis|NZHYM2978-20|NZAC03035247|BOLD:AAU8585
Trioxys sunnysidensis|NZHYM1558-12|NZAC04049134|BOLD:AAU8585
Trioxys sunnysidensis|NZHYM1547-12|NZAC04049339|BOLD:AAU8585
Trioxys sunnysidensis|NZHYM2993-20|NZAC03035259|BOLD:AAU8585
Trioxys sunnysidensis|NZBRX135-22|NZAC03035482|BOLD:AAU8585
Trioxys sunnysidensis|NZHYM1574-12|NZAC04049765|BOLD:AAU8585
Trioxys sunnysidensis|NZHYM1548-12|NZAC04049848|BOLD:AAU8585
Lysiphlebus testaceipes|NZHYM614-10|NZAC04034241|BOLD:AAG1226
Lysiphlebus testaceipes|NZHYM187-10|NZAC04033219|BOLD:AAG1226
Lysiphlebus testaceipes|NZHYM189-10|NZAC04035944|BOLD:AAG1226
Lysiphlebus testaceipes|NZHYM190-10|NZAC04036372|BOLD:AAG1226
Lysiphlebus testaceipes|NZHYM191-10|NZAC04036528|BOLD:AAG1226
Lysiphlebus testaceipes|NZHYM188-10|NZAC04037423|BOLD:AAG1226
Lysiphlebus testaceipes|NZHYM615-10|NZAC04037823|BOLD:AAG1226
Aphidius colemani|NZHYM773-11|NZAC04037302|BOLD:AAC7809
Aphidius colemani|NZHYM1563-12|NZAC04043878|BOLD:AAC7809
Aphidius colemani|NZHYM1562-12|NZAC04048786|BOLD:AAC7809
Aphidius|NZBRX207-22|NZAC03035556|BOLD:ACZ7916
Aphidius rhopalosiphi|NZHYM2988-20|NZAC03035254|BOLD:AFS3452
Aphidius rhopalosiphi|NZHYM1472-12|NZAC04048752|BOLD:AFS3452
Aphidius rhopalosiphi|NZMG371-12|NZAC04049071|BOLD:AFS3452
Aphidius rhopalosiphi|NZHYM2319-13|NZAC04086225|BOLD:AFS3452
Aphidius|NZHYM2269-13|NZAC04086428|BOLD:ABA1058
Metaspathius kuscheli|NZHYM721-11|NZAC04036800|BOLD:AAZ2912
Metaspathius kuscheli|NZHYM3067-20|NZAC03025407|BOLD:AAZ2912
Metaspathius kuscheli|NZHYM723-11|NZAC04037307|BOLD:AAZ2912
Metaspathius kuscheli|NZHYM724-11|NZAC04037569|BOLD:AAZ2912
Rhyssaloides|NZHYM2972-20|NZAC03035242|BOLD:AEE1535
Rhyssaloides|NZBRX027-22|NZAC03030578|BOLD:AEU4033
Rhyssaloides|NZBRX095-22|NZAC03035442|BOLD:AEU4033
Rhyssaloides|NZBRX044-22|NZAC03030594|BOLD:AEU4033
Rhyssaloides|NZBRX147-22|NZAC03035493|BOLD:AEU4033
Rhyssaloides|NZBRX217-22|NZAC03035566|BOLD:AEU4033
Rhyssaloides|NZBRX177-22|NZAC03035526|BOLD:AEU4033
Rhyssaloides|NZBRX018-22|NZAC03030571|BOLD:AEU4033
Rhyssaloides|NZBRX224-22|NZAC03035573|BOLD:AEU4033
Rhyssaloides|NZBRX176-22|NZAC03035525|BOLD:AEU4033
Rhyssaloides|NZBRX267-22|NZAC03035614|BOLD:AEU4033
Doryctomorpha antipoda|NZBRX178-22|NZAC03035527|BOLD:AAX9141
Doryctomorpha antipoda|NZBRX024-22|NZAC03030576|BOLD:AAX9141

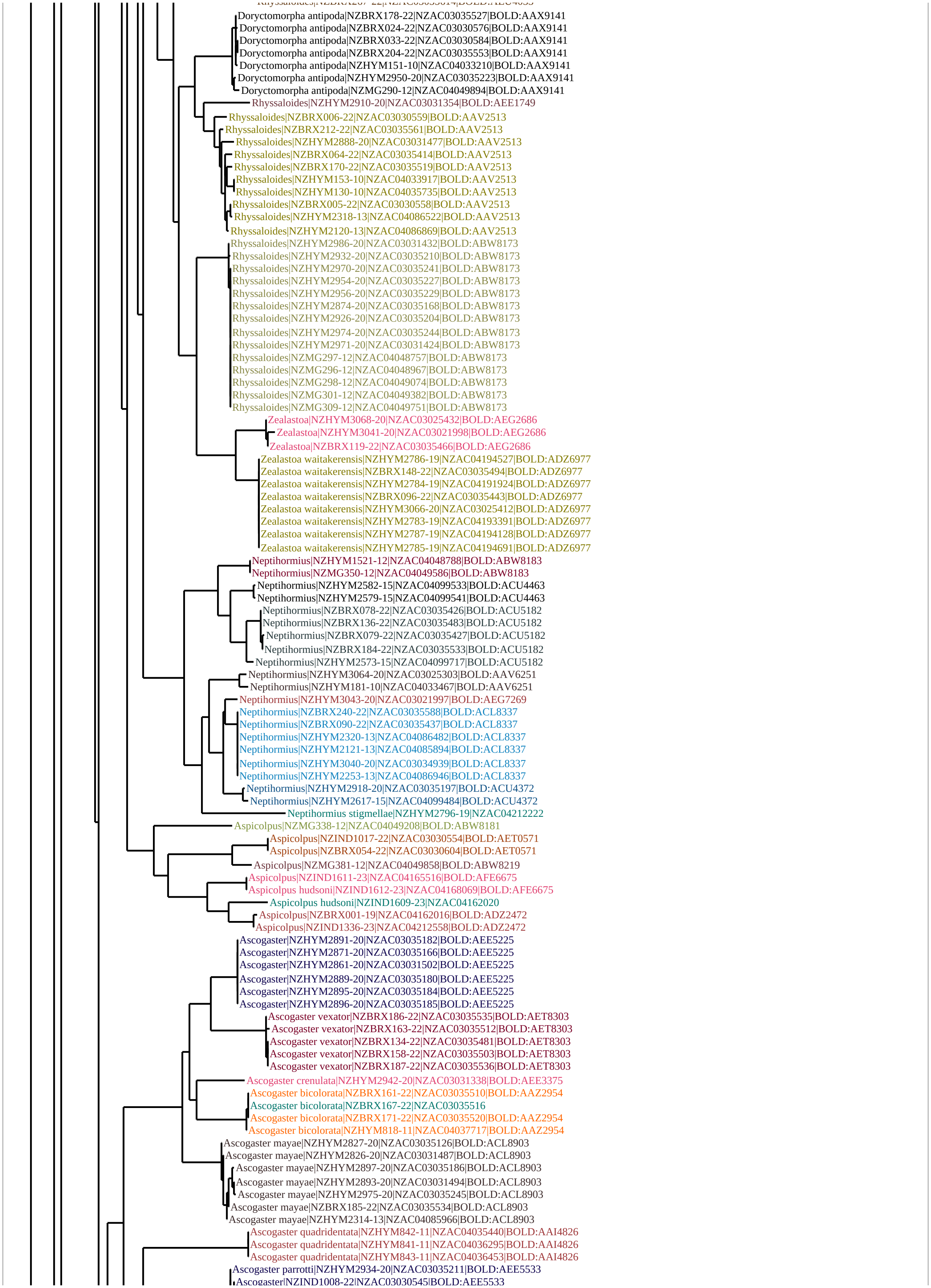
Doryctomorpha antipoda|NZBRX178-22|NZAC03035527|BOLD:AAX9141
Doryctomorpha antipoda|NZBRX024-22|NZAC03030576|BOLD:AAX9141
Doryctomorpha antipoda|NZBRX033-22|NZAC03030584|BOLD:AAX9141
Doryctomorpha antipoda|NZBRX204-22|NZAC03035553|BOLD:AAX9141
Doryctomorpha antipoda|NZHYM151-10|NZAC04033210|BOLD:AAX9141
Doryctomorpha antipoda|NZHYM2950-20|NZAC03035223|BOLD:AAX9141
Doryctomorpha antipoda|NZMG290-12|NZAC04049894|BOLD:AAX9141
Rhyssaloides|NZHYM2910-20|NZAC03031354|BOLD:AEE1749
Rhyssaloides|NZBRX006-22|NZAC03030559|BOLD:AAV2513
Rhyssaloides|NZBRX212-22|NZAC03035561|BOLD:AAV2513
Rhyssaloides|NZHYM2888-20|NZAC03031477|BOLD:AAV2513
Rhyssaloides|NZBRX064-22|NZAC03035414|BOLD:AAV2513
Rhyssaloides|NZBRX170-22|NZAC03035519|BOLD:AAV2513
Rhyssaloides|NZHYM153-10|NZAC04033917|BOLD:AAV2513
Rhyssaloides|NZHYM130-10|NZAC04035735|BOLD:AAV2513
Rhyssaloides|NZBRX005-22|NZAC03030558|BOLD:AAV2513
Rhyssaloides|NZHYM2318-13|NZAC04086522|BOLD:AAV2513
Rhyssaloides|NZHYM2120-13|NZAC04086869|BOLD:AAV2513
Rhyssaloides|NZHYM2986-20|NZAC03031432|BOLD:ABW8173
Rhyssaloides|NZHYM2932-20|NZAC03035210|BOLD:ABW8173
Rhyssaloides|NZHYM2970-20|NZAC03035241|BOLD:ABW8173
Rhyssaloides|NZHYM2954-20|NZAC03035227|BOLD:ABW8173
Rhyssaloides|NZHYM2956-20|NZAC03035229|BOLD:ABW8173
Rhyssaloides|NZHYM2874-20|NZAC03035168|BOLD:ABW8173
Rhyssaloides|NZHYM2926-20|NZAC03035204|BOLD:ABW8173
Rhyssaloides|NZHYM2974-20|NZAC03035244|BOLD:ABW8173
Rhyssaloides|NZHYM2971-20|NZAC03031424|BOLD:ABW8173
Rhyssaloides|NZMG297-12|NZAC04048757|BOLD:ABW8173
Rhyssaloides|NZMG296-12|NZAC04048967|BOLD:ABW8173
Rhyssaloides|NZMG298-12|NZAC04049074|BOLD:ABW8173
Rhyssaloides|NZMG301-12|NZAC04049382|BOLD:ABW8173
Rhyssaloides|NZMG309-12|NZAC04049751|BOLD:ABW8173
Zealastoa|NZHYM3068-20|NZAC03025432|BOLD:AEG2686
Zealastoa|NZHYM3041-20|NZAC03021998|BOLD:AEG2686
Zealastoa|NZBRX119-22|NZAC03035466|BOLD:AEG2686
Zealastoa waitakerensis|NZHYM2786-19|NZAC04194527|BOLD:ADZ6977
Zealastoa waitakerensis|NZBRX148-22|NZAC03035494|BOLD:ADZ6977
Zealastoa waitakerensis|NZHYM2784-19|NZAC04191924|BOLD:ADZ6977
Zealastoa waitakerensis|NZBRX096-22|NZAC03035443|BOLD:ADZ6977
Zealastoa waitakerensis|NZHYM3066-20|NZAC03025412|BOLD:ADZ6977
Zealastoa waitakerensis|NZHYM2783-19|NZAC04193391|BOLD:ADZ6977
Zealastoa waitakerensis|NZHYM2787-19|NZAC04194128|BOLD:ADZ6977
Zealastoa waitakerensis|NZHYM2785-19|NZAC04194691|BOLD:ADZ6977
Neptihormius|NZHYM1521-12|NZAC04048788|BOLD:ABW8183
Neptihormius|NZMG350-12|NZAC04049586|BOLD:ABW8183
Neptihormius|NZHYM2582-15|NZAC04099533|BOLD:ACU4463
Neptihormius|NZHYM2579-15|NZAC04099541|BOLD:ACU4463
Neptihormius|NZBRX078-22|NZAC03035426|BOLD:ACU5182
Neptihormius|NZBRX136-22|NZAC03035483|BOLD:ACU5182
Neptihormius|NZBRX079-22|NZAC03035427|BOLD:ACU5182
Neptihormius|NZBRX184-22|NZAC03035533|BOLD:ACU5182
Neptihormius|NZHYM2573-15|NZAC04099717|BOLD:ACU5182
Neptihormius|NZHYM3064-20|NZAC03025303|BOLD:AAV6251
Neptihormius|NZHYM181-10|NZAC04033467|BOLD:AAV6251
Neptihormius|NZHYM3043-20|NZAC03021997|BOLD:AEG7269
Neptihormius|NZBRX240-22|NZAC03035588|BOLD:ACL8337
Neptihormius|NZBRX090-22|NZAC03035437|BOLD:ACL8337
Neptihormius|NZHYM2320-13|NZAC04086482|BOLD:ACL8337
Neptihormius|NZHYM2121-13|NZAC04085894|BOLD:ACL8337
Neptihormius|NZHYM3040-20|NZAC03034939|BOLD:ACL8337
Neptihormius|NZHYM2253-13|NZAC04086946|BOLD:ACL8337
Neptihormius|NZHYM2918-20|NZAC03035197|BOLD:ACU4372
Neptihormius|NZHYM2617-15|NZAC04099484|BOLD:ACU4372
Neptihormius stigmellae|NZHYM2796-19|NZAC04212222
Aspicolpus|NZMG338-12|NZAC04049208|BOLD:ABW8181
Aspicolpus|NZIND1017-22|NZAC03030554|BOLD:AET0571
Aspicolpus|NZBRX054-22|NZAC03030604|BOLD:AET0571
Aspicolpus|NZMG381-12|NZAC04049858|BOLD:ABW8219
Aspicolpus|NZIND1611-23|NZAC04165516|BOLD:AFE6675
Aspicolpus hudsoni|NZIND1612-23|NZAC04168069|BOLD:AFE6675
Aspicolpus hudsoni|NZIND1609-23|NZAC04162020
Aspicolpus|NZBRX001-19|NZAC04162016|BOLD:ADZ2472
Aspicolpus|NZIND1336-23|NZAC04212558|BOLD:ADZ2472
Ascogaster|NZHYM2891-20|NZAC03035182|BOLD:AEE5225
Ascogaster|NZHYM2871-20|NZAC03035166|BOLD:AEE5225
Ascogaster|NZHYM2861-20|NZAC03031502|BOLD:AEE5225
Ascogaster|NZHYM2889-20|NZAC03035180|BOLD:AEE5225
Ascogaster|NZHYM2895-20|NZAC03035184|BOLD:AEE5225
Ascogaster|NZHYM2896-20|NZAC03035185|BOLD:AEE5225
Ascogaster vexator|NZBRX186-22|NZAC03035535|BOLD:AET8303
Ascogaster vexator|NZBRX163-22|NZAC03035512|BOLD:AET8303
Ascogaster vexator|NZBRX134-22|NZAC03035481|BOLD:AET8303
Ascogaster vexator|NZBRX158-22|NZAC03035503|BOLD:AET8303
Ascogaster vexator|NZBRX187-22|NZAC03035536|BOLD:AET8303
Ascogaster crenulata|NZHYM2942-20|NZAC03031338|BOLD:AEE3375
Ascogaster bicolorata|NZBRX161-22|NZAC03035510|BOLD:AAZ2954
Ascogaster bicolorata|NZBRX167-22|NZAC03035516
Ascogaster bicolorata|NZBRX171-22|NZAC03035520|BOLD:AAZ2954
Ascogaster bicolorata|NZHYM818-11|NZAC04037717|BOLD:AAZ2954
Ascogaster mayae|NZHYM2827-20|NZAC03035126|BOLD:ACL8903
Ascogaster mayae|NZHYM2826-20|NZAC03031487|BOLD:ACL8903
Ascogaster mayae|NZHYM2897-20|NZAC03035186|BOLD:ACL8903
Ascogaster mayae|NZHYM2893-20|NZAC03031494|BOLD:ACL8903
Ascogaster mayae|NZHYM2975-20|NZAC03035245|BOLD:ACL8903
Ascogaster mayae|NZBRX185-22|NZAC03035534|BOLD:ACL8903
Ascogaster mayae|NZHYM2314-13|NZAC04085966|BOLD:ACL8903
Ascogaster quadridentata|NZHYM842-11|NZAC04035440|BOLD:AAI4826
Ascogaster quadridentata|NZHYM841-11|NZAC04036295|BOLD:AAI4826
Ascogaster quadridentata|NZHYM843-11|NZAC04036453|BOLD:AAI4826
Ascogaster parrotti|NZHYM2934-20|NZAC03035211|BOLD:AEE5533
Ascogaster|NZIND1008-22|NZAC03030545|BOLD:AEE5533

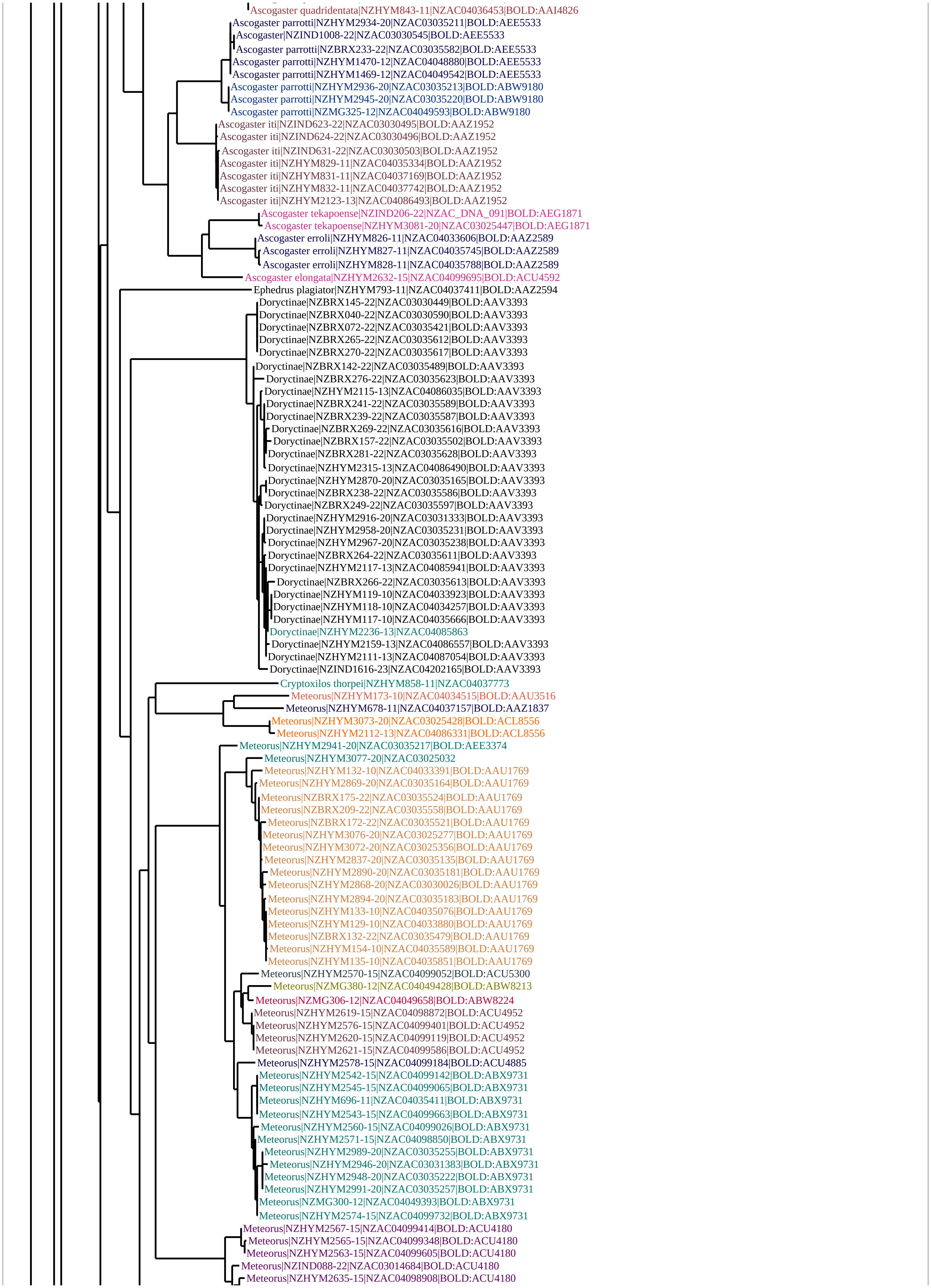
Ascogaster quadridentata|NZHYM843-11|NZAC04036453|BOLD:AAI4826
Ascogaster parrotti|NZHYM2934-20|NZAC03035211|BOLD:AEE5533
Ascogaster|NZIND1008-22|NZAC03030545|BOLD:AEE5533
Ascogaster parrotti|NZBRX233-22|NZAC03035582|BOLD:AEE5533
Ascogaster parrotti|NZHYM1470-12|NZAC04048880|BOLD:AEE5533
Ascogaster parrotti|NZHYM1469-12|NZAC04049542|BOLD:AEE5533
Ascogaster parrotti|NZHYM2936-20|NZAC03035213|BOLD:ABW9180
Ascogaster parrotti|NZHYM2945-20|NZAC03035220|BOLD:ABW9180
Ascogaster parrotti|NZMG325-12|NZAC04049593|BOLD:ABW9180
Ascogaster iti|NZIND623-22|NZAC03030495|BOLD:AAZ1952
Ascogaster iti|NZIND624-22|NZAC03030496|BOLD:AAZ1952
Ascogaster iti|NZIND631-22|NZAC03030503|BOLD:AAZ1952
Ascogaster iti|NZHYM829-11|NZAC04035334|BOLD:AAZ1952
Ascogaster iti|NZHYM831-11|NZAC04037169|BOLD:AAZ1952
Ascogaster iti|NZHYM832-11|NZAC04037742|BOLD:AAZ1952
Ascogaster iti|NZHYM2123-13|NZAC04086493|BOLD:AAZ1952
Ascogaster tekapoense|NZIND206-22|NZAC_DNA_091|BOLD:AEG1871
Ascogaster tekapoense|NZHYM3081-20|NZAC03025447|BOLD:AEG1871
Ascogaster erroli|NZHYM826-11|NZAC04033606|BOLD:AAZ2589
Ascogaster erroli|NZHYM827-11|NZAC04035745|BOLD:AAZ2589
Ascogaster erroli|NZHYM828-11|NZAC04035788|BOLD:AAZ2589
Ascogaster elongata|NZHYM2632-15|NZAC04099695|BOLD:ACU4592
Ephedrus plagiator|NZHYM793-11|NZAC04037411|BOLD:AAZ2594
Doryctinae|NZBRX145-22|NZAC03030449|BOLD:AAV3393
Doryctinae|NZBRX040-22|NZAC03030590|BOLD:AAV3393
Doryctinae|NZBRX072-22|NZAC03035421|BOLD:AAV3393
Doryctinae|NZBRX265-22|NZAC03035612|BOLD:AAV3393
Doryctinae|NZBRX270-22|NZAC03035617|BOLD:AAV3393
Doryctinae|NZBRX142-22|NZAC03035489|BOLD:AAV3393
Doryctinae|NZBRX276-22|NZAC03035623|BOLD:AAV3393
Doryctinae|NZHYM2115-13|NZAC04086035|BOLD:AAV3393
Doryctinae|NZBRX241-22|NZAC03035589|BOLD:AAV3393
Doryctinae|NZBRX239-22|NZAC03035587|BOLD:AAV3393
Doryctinae|NZBRX269-22|NZAC03035616|BOLD:AAV3393
Doryctinae|NZBRX157-22|NZAC03035502|BOLD:AAV3393
Doryctinae|NZBRX281-22|NZAC03035628|BOLD:AAV3393
Doryctinae|NZHYM2315-13|NZAC04086490|BOLD:AAV3393
Doryctinae|NZHYM2870-20|NZAC03035165|BOLD:AAV3393
Doryctinae|NZBRX238-22|NZAC03035586|BOLD:AAV3393
Doryctinae|NZBRX249-22|NZAC03035597|BOLD:AAV3393
Doryctinae|NZHYM2916-20|NZAC03031333|BOLD:AAV3393
Doryctinae|NZHYM2958-20|NZAC03035231|BOLD:AAV3393
Doryctinae|NZHYM2967-20|NZAC03035238|BOLD:AAV3393
Doryctinae|NZBRX264-22|NZAC03035611|BOLD:AAV3393
Doryctinae|NZHYM2117-13|NZAC04085941|BOLD:AAV3393
Doryctinae|NZBRX266-22|NZAC03035613|BOLD:AAV3393
Doryctinae|NZHYM119-10|NZAC04033923|BOLD:AAV3393
Doryctinae|NZHYM118-10|NZAC04034257|BOLD:AAV3393
Doryctinae|NZHYM117-10|NZAC04035666|BOLD:AAV3393
Doryctinae|NZHYM2236-13|NZAC04085863
Doryctinae|NZHYM2159-13|NZAC04086557|BOLD:AAV3393
Doryctinae|NZHYM2111-13|NZAC04087054|BOLD:AAV3393
Doryctinae|NZIND1616-23|NZAC04202165|BOLD:AAV3393
Cryptoxilos thorpei|NZHYM858-11|NZAC04037773
Meteorus|NZHYM173-10|NZAC04034515|BOLD:AAU3516
Meteorus|NZHYM678-11|NZAC04037157|BOLD:AAZ1837
Meteorus|NZHYM3073-20|NZAC03025428|BOLD:ACL8556
Meteorus|NZHYM2112-13|NZAC04086331|BOLD:ACL8556
Meteorus|NZHYM2941-20|NZAC03035217|BOLD:AEE3374
Meteorus|NZHYM3077-20|NZAC03025032
Meteorus|NZHYM132-10|NZAC04033391|BOLD:AAU1769
Meteorus|NZHYM2869-20|NZAC03035164|BOLD:AAU1769
Meteorus|NZBRX175-22|NZAC03035524|BOLD:AAU1769
Meteorus|NZBRX209-22|NZAC03035558|BOLD:AAU1769
Meteorus|NZBRX172-22|NZAC03035521|BOLD:AAU1769
Meteorus|NZHYM3076-20|NZAC03025277|BOLD:AAU1769
Meteorus|NZHYM3072-20|NZAC03025356|BOLD:AAU1769
Meteorus|NZHYM2837-20|NZAC03035135|BOLD:AAU1769
Meteorus|NZHYM2890-20|NZAC03035181|BOLD:AAU1769
Meteorus|NZHYM2868-20|NZAC03030026|BOLD:AAU1769
Meteorus|NZHYM2894-20|NZAC03035183|BOLD:AAU1769
Meteorus|NZHYM133-10|NZAC04035076|BOLD:AAU1769
Meteorus|NZHYM129-10|NZAC04033880|BOLD:AAU1769
Meteorus|NZBRX132-22|NZAC03035479|BOLD:AAU1769
Meteorus|NZHYM154-10|NZAC04035589|BOLD:AAU1769
Meteorus|NZHYM135-10|NZAC04035851|BOLD:AAU1769
Meteorus|NZHYM2570-15|NZAC04099052|BOLD:ACU5300
Meteorus|NZMG380-12|NZAC04049428|BOLD:ABW8213
Meteorus|NZMG306-12|NZAC04049658|BOLD:ABW8224
Meteorus|NZHYM2619-15|NZAC04098872|BOLD:ACU4952
Meteorus|NZHYM2576-15|NZAC04099401|BOLD:ACU4952
Meteorus|NZHYM2620-15|NZAC04099119|BOLD:ACU4952
Meteorus|NZHYM2621-15|NZAC04099586|BOLD:ACU4952
Meteorus|NZHYM2578-15|NZAC04099184|BOLD:ACU4885
Meteorus|NZHYM2542-15|NZAC04099142|BOLD:ABX9731
Meteorus|NZHYM2545-15|NZAC04099065|BOLD:ABX9731
Meteorus|NZHYM696-11|NZAC04035411|BOLD:ABX9731
Meteorus|NZHYM2543-15|NZAC04099663|BOLD:ABX9731
Meteorus|NZHYM2560-15|NZAC04099026|BOLD:ABX9731
Meteorus|NZHYM2571-15|NZAC04098850|BOLD:ABX9731
Meteorus|NZHYM2989-20|NZAC03035255|BOLD:ABX9731
Meteorus|NZHYM2946-20|NZAC03031383|BOLD:ABX9731
Meteorus|NZHYM2948-20|NZAC03035222|BOLD:ABX9731
Meteorus|NZHYM2991-20|NZAC03035257|BOLD:ABX9731
Meteorus|NZMG300-12|NZAC04049393|BOLD:ABX9731
Meteorus|NZHYM2574-15|NZAC04099732|BOLD:ABX9731
Meteorus|NZHYM2567-15|NZAC04099414|BOLD:ACU4180
Meteorus|NZHYM2565-15|NZAC04099348|BOLD:ACU4180
Meteorus|NZHYM2563-15|NZAC04099605|BOLD:ACU4180
Meteorus|NZIND088-22|NZAC03014684|BOLD:ACU4180
Meteorus|NZHYM2635-15|NZAC04098908|BOLD:ACU4180

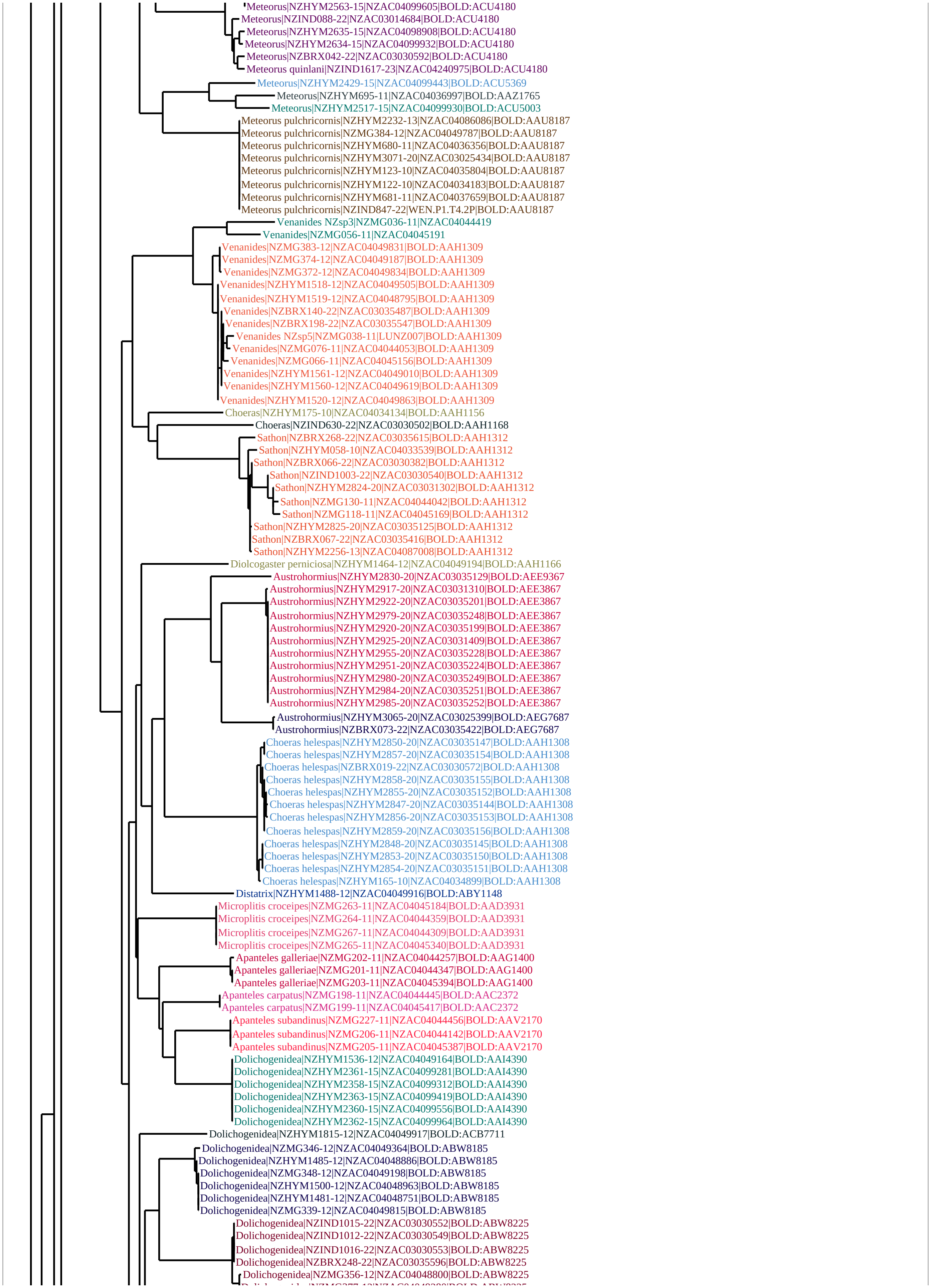
Meteorus|NZHYM2563-15|NZAC04099605|BOLD:ACU4180
Meteorus|NZIND088-22|NZAC03014684|BOLD:ACU4180
Meteorus|NZHYM2635-15|NZAC04098908|BOLD:ACU4180
Meteorus|NZHYM2634-15|NZAC04099932|BOLD:ACU4180
Meteorus|NZBRX042-22|NZAC03030592|BOLD:ACU4180
Meteorus quinlani|NZIND1617-23|NZAC04240975|BOLD:ACU4180
Meteorus|NZHYM2429-15|NZAC04099443|BOLD:ACU5369
Meteorus|NZHYM695-11|NZAC04036997|BOLD:AAZ1765
Meteorus|NZHYM2517-15|NZAC04099930|BOLD:ACU5003
Meteorus pulchricornis|NZHYM2232-13|NZAC04086086|BOLD:AAU8187
Meteorus pulchricornis|NZMG384-12|NZAC04049787|BOLD:AAU8187
Meteorus pulchricornis|NZHYM680-11|NZAC04036356|BOLD:AAU8187
Meteorus pulchricornis|NZHYM3071-20|NZAC03025434|BOLD:AAU8187
Meteorus pulchricornis|NZHYM123-10|NZAC04035804|BOLD:AAU8187
Meteorus pulchricornis|NZHYM122-10|NZAC04034183|BOLD:AAU8187
Meteorus pulchricornis|NZHYM681-11|NZAC04037659|BOLD:AAU8187
Meteorus pulchricornis|NZIND847-22|WEN.P1.T4.2P|BOLD:AAU8187
Venanides NZsp3|NZMG036-11|NZAC04044419
Venanides|NZMG056-11|NZAC04045191
Venanides|NZMG383-12|NZAC04049831|BOLD:AAH1309
Venanides|NZMG374-12|NZAC04049187|BOLD:AAH1309
Venanides|NZMG372-12|NZAC04049834|BOLD:AAH1309
Venanides|NZHYM1518-12|NZAC04049505|BOLD:AAH1309
Venanides|NZHYM1519-12|NZAC04048795|BOLD:AAH1309
Venanides|NZBRX140-22|NZAC03035487|BOLD:AAH1309
Venanides|NZBRX198-22|NZAC03035547|BOLD:AAH1309
Venanides NZsp5|NZMG038-11|LUNZ007|BOLD:AAH1309
Venanides|NZMG076-11|NZAC04044053|BOLD:AAH1309
Venanides|NZMG066-11|NZAC04045156|BOLD:AAH1309
Venanides|NZHYM1561-12|NZAC04049010|BOLD:AAH1309
Venanides|NZHYM1560-12|NZAC04049619|BOLD:AAH1309
Venanides|NZHYM1520-12|NZAC04049863|BOLD:AAH1309
Choeras|NZHYM175-10|NZAC04034134|BOLD:AAH1156
Choeras|NZIND630-22|NZAC03030502|BOLD:AAH1168
Sathon|NZBRX268-22|NZAC03035615|BOLD:AAH1312
Sathon|NZHYM058-10|NZAC04033539|BOLD:AAH1312
Sathon|NZBRX066-22|NZAC03030382|BOLD:AAH1312
Sathon|NZIND1003-22|NZAC03030540|BOLD:AAH1312
Sathon|NZHYM2824-20|NZAC03031302|BOLD:AAH1312
Sathon|NZMG130-11|NZAC04044042|BOLD:AAH1312
Sathon|NZMG118-11|NZAC04045169|BOLD:AAH1312
Sathon|NZHYM2825-20|NZAC03035125|BOLD:AAH1312
Sathon|NZBRX067-22|NZAC03035416|BOLD:AAH1312
Sathon|NZHYM2256-13|NZAC04087008|BOLD:AAH1312
Diolcogaster perniciosa|NZHYM1464-12|NZAC04049194|BOLD:AAH1166
Austrohormius|NZHYM2830-20|NZAC03035129|BOLD:AEE9367
Austrohormius|NZHYM2917-20|NZAC03031310|BOLD:AEE3867
Austrohormius|NZHYM2922-20|NZAC03035201|BOLD:AEE3867
Austrohormius|NZHYM2979-20|NZAC03035248|BOLD:AEE3867
Austrohormius|NZHYM2920-20|NZAC03035199|BOLD:AEE3867
Austrohormius|NZHYM2925-20|NZAC03031409|BOLD:AEE3867
Austrohormius|NZHYM2955-20|NZAC03035228|BOLD:AEE3867
Austrohormius|NZHYM2951-20|NZAC03035224|BOLD:AEE3867
Austrohormius|NZHYM2980-20|NZAC03035249|BOLD:AEE3867
Austrohormius|NZHYM2984-20|NZAC03035251|BOLD:AEE3867
Austrohormius|NZHYM2985-20|NZAC03035252|BOLD:AEE3867
Austrohormius|NZHYM3065-20|NZAC03025399|BOLD:AEG7687
Austrohormius|NZBRX073-22|NZAC03035422|BOLD:AEG7687
Choeras helespas|NZHYM2850-20|NZAC03035147|BOLD:AAH1308
Choeras helespas|NZHYM2857-20|NZAC03035154|BOLD:AAH1308
Choeras helespas|NZBRX019-22|NZAC03030572|BOLD:AAH1308
Choeras helespas|NZHYM2858-20|NZAC03035155|BOLD:AAH1308
Choeras helespas|NZHYM2855-20|NZAC03035152|BOLD:AAH1308
Choeras helespas|NZHYM2847-20|NZAC03035144|BOLD:AAH1308
Choeras helespas|NZHYM2856-20|NZAC03035153|BOLD:AAH1308
Choeras helespas|NZHYM2859-20|NZAC03035156|BOLD:AAH1308
Choeras helespas|NZHYM2848-20|NZAC03035145|BOLD:AAH1308
Choeras helespas|NZHYM2853-20|NZAC03035150|BOLD:AAH1308
Choeras helespas|NZHYM2854-20|NZAC03035151|BOLD:AAH1308
Choeras helespas|NZHYM165-10|NZAC04034899|BOLD:AAH1308
Distatrix|NZHYM1488-12|NZAC04049916|BOLD:ABY1148
Microplitis croceipes|NZMG263-11|NZAC04045184|BOLD:AAD3931
Microplitis croceipes|NZMG264-11|NZAC04044359|BOLD:AAD3931
Microplitis croceipes|NZMG267-11|NZAC04044309|BOLD:AAD3931
Microplitis croceipes|NZMG265-11|NZAC04045340|BOLD:AAD3931
Apanteles galleriae|NZMG202-11|NZAC04044257|BOLD:AAG1400
Apanteles galleriae|NZMG201-11|NZAC04044347|BOLD:AAG1400
Apanteles galleriae|NZMG203-11|NZAC04045394|BOLD:AAG1400
Apanteles carpatus|NZMG198-11|NZAC04044445|BOLD:AAC2372
Apanteles carpatus|NZMG199-11|NZAC04045417|BOLD:AAC2372
Apanteles subandinus|NZMG227-11|NZAC04044456|BOLD:AAV2170
Apanteles subandinus|NZMG206-11|NZAC04044142|BOLD:AAV2170
Apanteles subandinus|NZMG205-11|NZAC04045387|BOLD:AAV2170
Dolichogenidea|NZHYM1536-12|NZAC04049164|BOLD:AAI4390
Dolichogenidea|NZHYM2361-15|NZAC04099281|BOLD:AAI4390
Dolichogenidea|NZHYM2358-15|NZAC04099312|BOLD:AAI4390
Dolichogenidea|NZHYM2363-15|NZAC04099419|BOLD:AAI4390
Dolichogenidea|NZHYM2360-15|NZAC04099556|BOLD:AAI4390
Dolichogenidea|NZHYM2362-15|NZAC04099964|BOLD:AAI4390
Dolichogenidea|NZHYM1815-12|NZAC04049917|BOLD:ACB7711
Dolichogenidea|NZMG346-12|NZAC04049364|BOLD:ABW8185
Dolichogenidea|NZHYM1485-12|NZAC04048886|BOLD:ABW8185
Dolichogenidea|NZMG348-12|NZAC04049198|BOLD:ABW8185
Dolichogenidea|NZHYM1500-12|NZAC04048963|BOLD:ABW8185
Dolichogenidea|NZHYM1481-12|NZAC04048751|BOLD:ABW8185
Dolichogenidea|NZMG339-12|NZAC04049815|BOLD:ABW8185
Dolichogenidea|NZIND1015-22|NZAC03030552|BOLD:ABW8225
Dolichogenidea|NZIND1012-22|NZAC03030549|BOLD:ABW8225
Dolichogenidea|NZIND1016-22|NZAC03030553|BOLD:ABW8225
Dolichogenidea|NZBRX248-22|NZAC03035596|BOLD:ABW8225
Dolichogenidea|NZMG356-12|NZAC04048800|BOLD:ABW8225

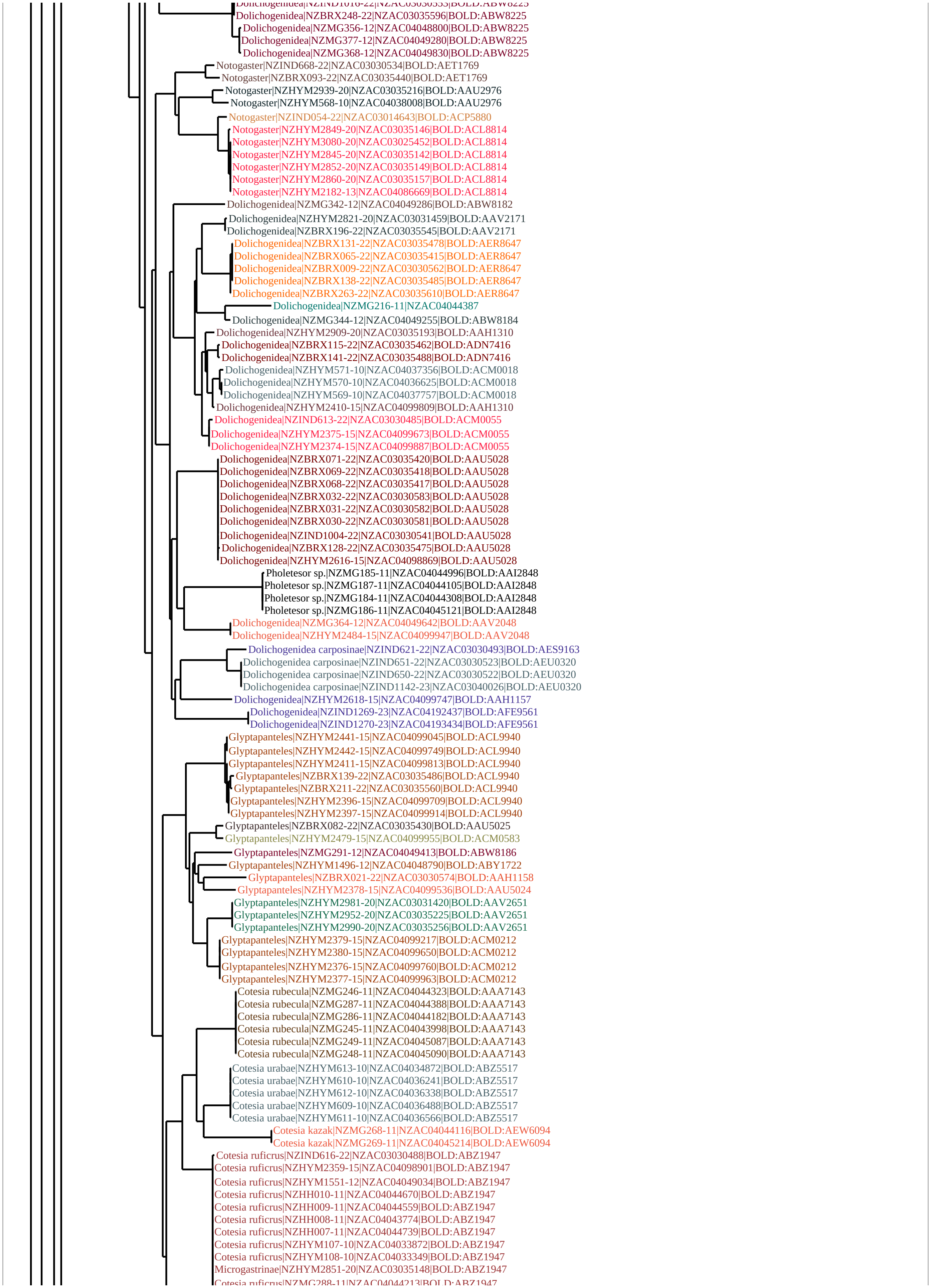
Dolichogenidea|NZIND1018-22|NZAC03030533|BOLD:ABW8225
Dolichogenidea|NZBRX248-22|NZAC03035596|BOLD:ABW8225
Dolichogenidea|NZMG356-12|NZAC04048800|BOLD:ABW8225
Dolichogenidea|NZMG377-12|NZAC04049280|BOLD:ABW8225
Dolichogenidea|NZMG368-12|NZAC04049830|BOLD:ABW8225
Notogaster|NZIND668-22|NZAC03030534|BOLD:AET1769
Notogaster|NZBRX093-22|NZAC03035440|BOLD:AET1769
Notogaster|NZHYM2939-20|NZAC03035216|BOLD:AAU2976
Notogaster|NZHYM568-10|NZAC04038008|BOLD:AAU2976
Notogaster|NZIND054-22|NZAC03014643|BOLD:ACP5880
Notogaster|NZHYM2849-20|NZAC03035146|BOLD:ACL8814
Notogaster|NZHYM3080-20|NZAC03025452|BOLD:ACL8814
Notogaster|NZHYM2845-20|NZAC03035142|BOLD:ACL8814
Notogaster|NZHYM2852-20|NZAC03035149|BOLD:ACL8814
Notogaster|NZHYM2860-20|NZAC03035157|BOLD:ACL8814
Notogaster|NZHYM2182-13|NZAC04086669|BOLD:ACL8814
Dolichogenidea|NZMG342-12|NZAC04049286|BOLD:ABW8182
Dolichogenidea|NZHYM2821-20|NZAC03031459|BOLD:AAV2171
Dolichogenidea|NZBRX196-22|NZAC03035545|BOLD:AAV2171
Dolichogenidea|NZBRX131-22|NZAC03035478|BOLD:AER8647
Dolichogenidea|NZBRX065-22|NZAC03035415|BOLD:AER8647
Dolichogenidea|NZBRX009-22|NZAC03030562|BOLD:AER8647
Dolichogenidea|NZBRX138-22|NZAC03035485|BOLD:AER8647
Dolichogenidea|NZBRX263-22|NZAC03035610|BOLD:AER8647
Dolichogenidea|NZMG216-11|NZAC04044387
Dolichogenidea|NZMG344-12|NZAC04049255|BOLD:ABW8184
Dolichogenidea|NZHYM2909-20|NZAC03035193|BOLD:AAH1310
Dolichogenidea|NZBRX115-22|NZAC03035462|BOLD:ADN7416
Dolichogenidea|NZBRX141-22|NZAC03035488|BOLD:ADN7416
Dolichogenidea|NZHYM571-10|NZAC04037356|BOLD:ACM0018
Dolichogenidea|NZHYM570-10|NZAC04036625|BOLD:ACM0018
Dolichogenidea|NZHYM569-10|NZAC04037757|BOLD:ACM0018
Dolichogenidea|NZHYM2410-15|NZAC04099809|BOLD:AAH1310
Dolichogenidea|NZIND613-22|NZAC03030485|BOLD:ACM0055
Dolichogenidea|NZHYM2375-15|NZAC04099673|BOLD:ACM0055
Dolichogenidea|NZHYM2374-15|NZAC04099887|BOLD:ACM0055
Dolichogenidea|NZBRX071-22|NZAC03035420|BOLD:AAU5028
Dolichogenidea|NZBRX069-22|NZAC03035418|BOLD:AAU5028
Dolichogenidea|NZBRX068-22|NZAC03035417|BOLD:AAU5028
Dolichogenidea|NZBRX032-22|NZAC03030583|BOLD:AAU5028
Dolichogenidea|NZBRX031-22|NZAC03030582|BOLD:AAU5028
Dolichogenidea|NZBRX030-22|NZAC03030581|BOLD:AAU5028
Dolichogenidea|NZIND1004-22|NZAC03030541|BOLD:AAU5028
Dolichogenidea|NZBRX128-22|NZAC03035475|BOLD:AAU5028
Dolichogenidea|NZHYM2616-15|NZAC04098869|BOLD:AAU5028
Pholetesor sp.|NZMG185-11|NZAC04044996|BOLD:AAI2848
Pholetesor sp.|NZMG187-11|NZAC04044105|BOLD:AAI2848
Pholetesor sp.|NZMG184-11|NZAC04044308|BOLD:AAI2848
Pholetesor sp.|NZMG186-11|NZAC04045121|BOLD:AAI2848
Dolichogenidea|NZMG364-12|NZAC04049642|BOLD:AAV2048
Dolichogenidea|NZHYM2484-15|NZAC04099947|BOLD:AAV2048
Dolichogenidea carposinae|NZIND621-22|NZAC03030493|BOLD:AES9163
Dolichogenidea carposinae|NZIND651-22|NZAC03030523|BOLD:AEU0320
Dolichogenidea carposinae|NZIND650-22|NZAC03030522|BOLD:AEU0320
Dolichogenidea carposinae|NZIND1142-23|NZAC03040026|BOLD:AEU0320
Dolichogenidea|NZHYM2618-15|NZAC04099747|BOLD:AAH1157
Dolichogenidea|NZIND1269-23|NZAC04192437|BOLD:AFE9561
Dolichogenidea|NZIND1270-23|NZAC04193434|BOLD:AFE9561
Glyptapanteles|NZHYM2441-15|NZAC04099045|BOLD:ACL9940
Glyptapanteles|NZHYM2442-15|NZAC04099749|BOLD:ACL9940
Glyptapanteles|NZHYM2411-15|NZAC04099813|BOLD:ACL9940
Glyptapanteles|NZBRX139-22|NZAC03035486|BOLD:ACL9940
Glyptapanteles|NZBRX211-22|NZAC03035560|BOLD:ACL9940
Glyptapanteles|NZHYM2396-15|NZAC04099709|BOLD:ACL9940
Glyptapanteles|NZHYM2397-15|NZAC04099914|BOLD:ACL9940
Glyptapanteles|NZBRX082-22|NZAC03035430|BOLD:AAU5025
Glyptapanteles|NZHYM2479-15|NZAC04099955|BOLD:ACM0583
Glyptapanteles|NZMG291-12|NZAC04049413|BOLD:ABW8186
Glyptapanteles|NZHYM1496-12|NZAC04048790|BOLD:ABY1722
Glyptapanteles|NZBRX021-22|NZAC03030574|BOLD:AAH1158
Glyptapanteles|NZHYM2378-15|NZAC04099536|BOLD:AAU5024
Glyptapanteles|NZHYM2981-20|NZAC03031420|BOLD:AAV2651
Glyptapanteles|NZHYM2952-20|NZAC03035225|BOLD:AAV2651
Glyptapanteles|NZHYM2990-20|NZAC03035256|BOLD:AAV2651
Glyptapanteles|NZHYM2379-15|NZAC04099217|BOLD:ACM0212
Glyptapanteles|NZHYM2380-15|NZAC04099650|BOLD:ACM0212
Glyptapanteles|NZHYM2376-15|NZAC04099760|BOLD:ACM0212
Glyptapanteles|NZHYM2377-15|NZAC04099963|BOLD:ACM0212
Cotesia rubecula|NZMG246-11|NZAC04044323|BOLD:AAA7143
Cotesia rubecula|NZMG287-11|NZAC04044388|BOLD:AAA7143
Cotesia rubecula|NZMG286-11|NZAC04044182|BOLD:AAA7143
Cotesia rubecula|NZMG245-11|NZAC04043998|BOLD:AAA7143
Cotesia rubecula|NZMG249-11|NZAC04045087|BOLD:AAA7143
Cotesia rubecula|NZMG248-11|NZAC04045090|BOLD:AAA7143
Cotesia urabae|NZHYM613-10|NZAC04034872|BOLD:ABZ5517
Cotesia urabae|NZHYM610-10|NZAC04036241|BOLD:ABZ5517
Cotesia urabae|NZHYM612-10|NZAC04036338|BOLD:ABZ5517
Cotesia urabae|NZHYM609-10|NZAC04036488|BOLD:ABZ5517
Cotesia urabae|NZHYM611-10|NZAC04036566|BOLD:ABZ5517
Cotesia kazak|NZMG268-11|NZAC04044116|BOLD:AEW6094
Cotesia kazak|NZMG269-11|NZAC04045214|BOLD:AEW6094
Cotesia ruficrus|NZIND616-22|NZAC03030488|BOLD:ABZ1947
Cotesia ruficrus|NZHYM2359-15|NZAC04098901|BOLD:ABZ1947
Cotesia ruficrus|NZHYM1551-12|NZAC04049034|BOLD:ABZ1947
Cotesia ruficrus|NZHH010-11|NZAC04044670|BOLD:ABZ1947
Cotesia ruficrus|NZHH009-11|NZAC04044559|BOLD:ABZ1947
Cotesia ruficrus|NZHH008-11|NZAC04043774|BOLD:ABZ1947
Cotesia ruficrus|NZHH007-11|NZAC04044739|BOLD:ABZ1947
Cotesia ruficrus|NZHYM107-10|NZAC04033872|BOLD:ABZ1947
Cotesia ruficrus|NZHYM108-10|NZAC04033349|BOLD:ABZ1947
Microgastrinae|NZHYM2851-20|NZAC03035148|BOLD:ABZ1947
Cotesia ruficrus|NZMG288-11|NZAC04044213|BOLD:ABZ1947

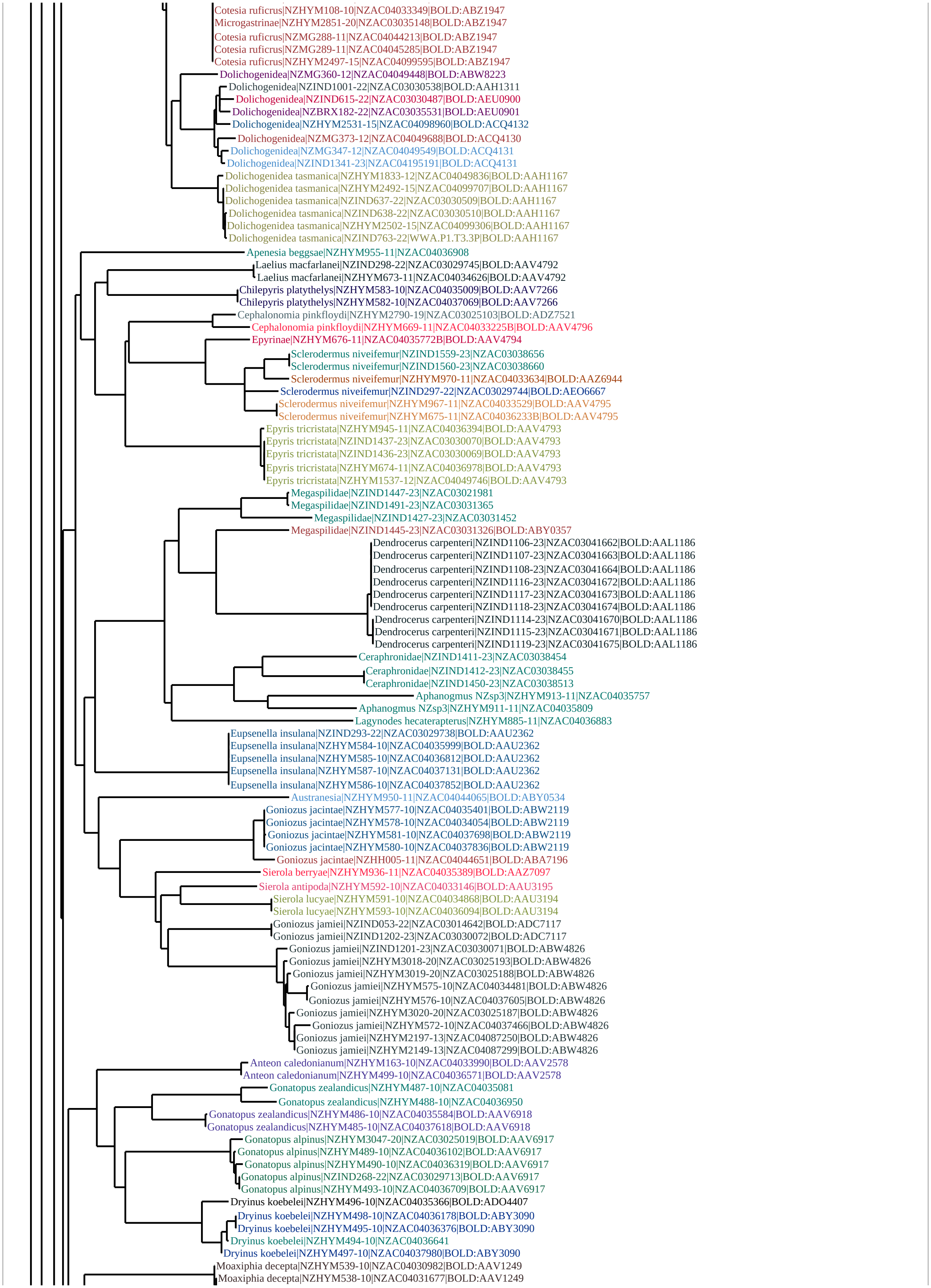
Cotesia ruficrus|NZHYM108-10|NZAC04033349|BOLD:ABZ1947
Microgastrinae|NZHYM2851-20|NZAC03035148|BOLD:ABZ1947
Cotesia ruficrus|NZMG288-11|NZAC04044213|BOLD:ABZ1947
Cotesia ruficrus|NZMG289-11|NZAC04045285|BOLD:ABZ1947
Cotesia ruficrus|NZHYM2497-15|NZAC04099595|BOLD:ABZ1947
Dolichogenidea|NZMG360-12|NZAC04049448|BOLD:ABW8223
Dolichogenidea|NZIND1001-22|NZAC03030538|BOLD:AAH1311
Dolichogenidea|NZIND615-22|NZAC03030487|BOLD:AEU0900
Dolichogenidea|NZBRX182-22|NZAC03035531|BOLD:AEU0901
Dolichogenidea|NZHYM2531-15|NZAC04098960|BOLD:ACQ4132
Dolichogenidea|NZMG373-12|NZAC04049688|BOLD:ACQ4130
Dolichogenidea|NZMG347-12|NZAC04049549|BOLD:ACQ4131
Dolichogenidea|NZIND1341-23|NZAC04195191|BOLD:ACQ4131
Dolichogenidea tasmanica|NZHYM1833-12|NZAC04049836|BOLD:AAH1167
Dolichogenidea tasmanica|NZHYM2492-15|NZAC04099707|BOLD:AAH1167
Dolichogenidea tasmanica|NZIND637-22|NZAC03030509|BOLD:AAH1167
Dolichogenidea tasmanica|NZIND638-22|NZAC03030510|BOLD:AAH1167
Dolichogenidea tasmanica|NZHYM2502-15|NZAC04099306|BOLD:AAH1167
Dolichogenidea tasmanica|NZIND763-22|WWA.P1.T3.3P|BOLD:AAH1167
Apenesia beggsae|NZHYM955-11|NZAC04036908
Laelius macfarlanei|NZIND298-22|NZAC03029745|BOLD:AAV4792
Laelius macfarlanei|NZHYM673-11|NZAC04034626|BOLD:AAV4792
Chilepyris platythelys|NZHYM583-10|NZAC04035009|BOLD:AAV7266
Chilepyris platythelys|NZHYM582-10|NZAC04037069|BOLD:AAV7266
Cephalonomia pinkfloydi|NZHYM2790-19|NZAC03025103|BOLD:ADZ7521
Cephalonomia pinkfloydi|NZHYM669-11|NZAC04033225B|BOLD:AAV4796
Epyrinae|NZHYM676-11|NZAC04035772B|BOLD:AAV4794
Sclerodermus niveifemur|NZIND1559-23|NZAC03038656
Sclerodermus niveifemur|NZIND1560-23|NZAC03038660
Sclerodermus niveifemur|NZHYM970-11|NZAC04033634|BOLD:AAZ6944
Sclerodermus niveifemur|NZIND297-22|NZAC03029744|BOLD:AEO6667
Sclerodermus niveifemur|NZHYM967-11|NZAC04033529|BOLD:AAV4795
Sclerodermus niveifemur|NZHYM675-11|NZAC04036233B|BOLD:AAV4795
Epyris tricristata|NZHYM945-11|NZAC04036394|BOLD:AAV4793
Epyris tricristata|NZIND1437-23|NZAC03030070|BOLD:AAV4793
Epyris tricristata|NZIND1436-23|NZAC03030069|BOLD:AAV4793
Epyris tricristata|NZHYM674-11|NZAC04036978|BOLD:AAV4793
Epyris tricristata|NZHYM1537-12|NZAC04049746|BOLD:AAV4793
Megaspilidae|NZIND1447-23|NZAC03021981
Megaspilidae|NZIND1491-23|NZAC03031365
Megaspilidae|NZIND1427-23|NZAC03031452
Megaspilidae|NZIND1445-23|NZAC03031326|BOLD:ABY0357
Dendrocerus carpenteri|NZIND1106-23|NZAC03041662|BOLD:AAL1186
Dendrocerus carpenteri|NZIND1107-23|NZAC03041663|BOLD:AAL1186
Dendrocerus carpenteri|NZIND1108-23|NZAC03041664|BOLD:AAL1186
Dendrocerus carpenteri|NZIND1116-23|NZAC03041672|BOLD:AAL1186
Dendrocerus carpenteri|NZIND1117-23|NZAC03041673|BOLD:AAL1186
Dendrocerus carpenteri|NZIND1118-23|NZAC03041674|BOLD:AAL1186
Dendrocerus carpenteri|NZIND1114-23|NZAC03041670|BOLD:AAL1186
Dendrocerus carpenteri|NZIND1115-23|NZAC03041671|BOLD:AAL1186
Dendrocerus carpenteri|NZIND1119-23|NZAC03041675|BOLD:AAL1186
Ceraphronidae|NZIND1411-23|NZAC03038454
Ceraphronidae|NZIND1412-23|NZAC03038455
Ceraphronidae|NZIND1450-23|NZAC03038513
Aphanogmus NZsp3|NZHYM913-11|NZAC04035757
Aphanogmus NZsp3|NZHYM911-11|NZAC04035809
Lagynodes hecaterapterus|NZHYM885-11|NZAC04036883
Eupsenella insulana|NZIND293-22|NZAC03029738|BOLD:AAU2362
Eupsenella insulana|NZHYM584-10|NZAC04035999|BOLD:AAU2362
Eupsenella insulana|NZHYM585-10|NZAC04036812|BOLD:AAU2362
Eupsenella insulana|NZHYM587-10|NZAC04037131|BOLD:AAU2362
Eupsenella insulana|NZHYM586-10|NZAC04037852|BOLD:AAU2362
Austranesia|NZHYM950-11|NZAC04044065|BOLD:ABY0534
Goniozus jacintae|NZHYM577-10|NZAC04035401|BOLD:ABW2119
Goniozus jacintae|NZHYM578-10|NZAC04034054|BOLD:ABW2119
Goniozus jacintae|NZHYM581-10|NZAC04037698|BOLD:ABW2119
Goniozus jacintae|NZHYM580-10|NZAC04037836|BOLD:ABW2119
Goniozus jacintae|NZHH005-11|NZAC04044651|BOLD:ABA7196
Sierola berryae|NZHYM936-11|NZAC04035389|BOLD:AAZ7097
Sierola antipoda|NZHYM592-10|NZAC04033146|BOLD:AAU3195
Sierola lucyae|NZHYM591-10|NZAC04034868|BOLD:AAU3194
Sierola lucyae|NZHYM593-10|NZAC04036094|BOLD:AAU3194
Goniozus jamiei|NZIND053-22|NZAC03014642|BOLD:ADC7117
Goniozus jamiei|NZIND1202-23|NZAC03030072|BOLD:ADC7117
Goniozus jamiei|NZIND1201-23|NZAC03030071|BOLD:ABW4826
Goniozus jamiei|NZHYM3018-20|NZAC03025193|BOLD:ABW4826
Goniozus jamiei|NZHYM3019-20|NZAC03025188|BOLD:ABW4826
Goniozus jamiei|NZHYM575-10|NZAC04034481|BOLD:ABW4826
Goniozus jamiei|NZHYM576-10|NZAC04037605|BOLD:ABW4826
Goniozus jamiei|NZHYM3020-20|NZAC03025187|BOLD:ABW4826
Goniozus jamiei|NZHYM572-10|NZAC04037466|BOLD:ABW4826
Goniozus jamiei|NZHYM2197-13|NZAC04087250|BOLD:ABW4826
Goniozus jamiei|NZHYM2149-13|NZAC04087299|BOLD:ABW4826
Anteon caledonianum|NZHYM163-10|NZAC04033990|BOLD:AAV2578
Anteon caledonianum|NZHYM499-10|NZAC04036571|BOLD:AAV2578
Gonatopus zealandicus|NZHYM487-10|NZAC04035081
Gonatopus zealandicus|NZHYM488-10|NZAC04036950
Gonatopus zealandicus|NZHYM486-10|NZAC04035584|BOLD:AAV6918
Gonatopus zealandicus|NZHYM485-10|NZAC04037618|BOLD:AAV6918
Gonatopus alpinus|NZHYM3047-20|NZAC03025019|BOLD:AAV6917
Gonatopus alpinus|NZHYM489-10|NZAC04036102|BOLD:AAV6917
Gonatopus alpinus|NZHYM490-10|NZAC04036319|BOLD:AAV6917
Gonatopus alpinus|NZIND268-22|NZAC03029713|BOLD:AAV6917
Gonatopus alpinus|NZHYM493-10|NZAC04036709|BOLD:AAV6917
Dryinus koebelei|NZHYM496-10|NZAC04035366|BOLD:ADO4407
Dryinus koebelei|NZHYM498-10|NZAC04036178|BOLD:ABY3090
Dryinus koebelei|NZHYM495-10|NZAC04036376|BOLD:ABY3090
Dryinus koebelei|NZHYM494-10|NZAC04036641
Dryinus koebelei|NZHYM497-10|NZAC04037980|BOLD:ABY3090
Moaxiphia decepta|NZHYM539-10|NZAC04030982|BOLD:AAV1249
Moaxiphia decepta|NZHYM538-10|NZAC04031677|BOLD:AAV1249

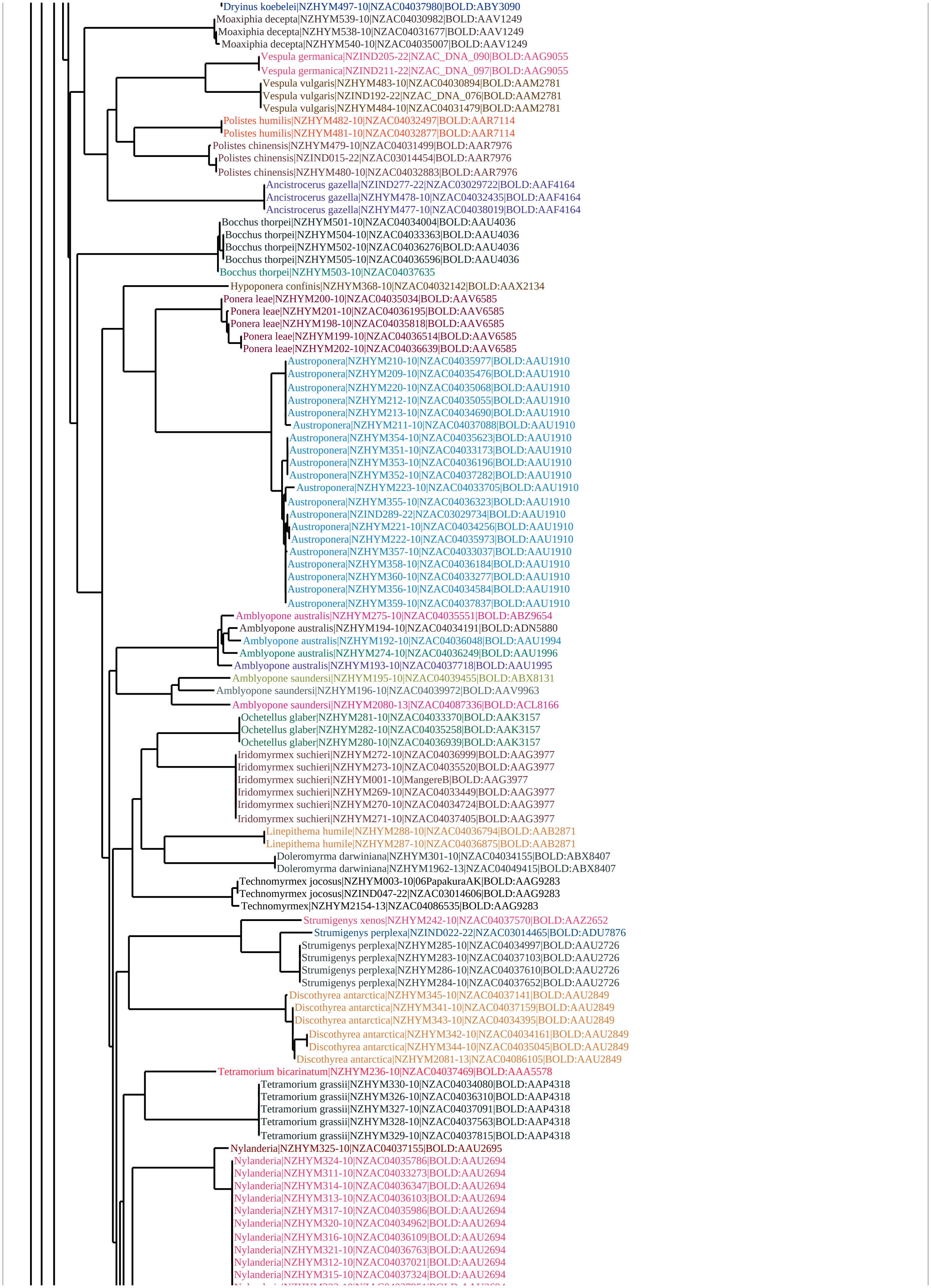
Dryinus koebelei|NZHYM497-10|NZAC04037980|BOLD:ABY3090
Moaxiphia decepta|NZHYM539-10|NZAC04030982|BOLD:AAV1249
Moaxiphia decepta|NZHYM538-10|NZAC04031677|BOLD:AAV1249
Moaxiphia decepta|NZHYM540-10|NZAC04035007|BOLD:AAV1249
Vespula germanica|NZIND205-22|NZAC_DNA_090|BOLD:AAG9055
Vespula germanica|NZIND211-22|NZAC_DNA_097|BOLD:AAG9055
Vespula vulgaris|NZHYM483-10|NZAC04030894|BOLD:AAM2781
Vespula vulgaris|NZIND192-22|NZAC_DNA_076|BOLD:AAM2781
Vespula vulgaris|NZHYM484-10|NZAC04031479|BOLD:AAM2781
Polistes humilis|NZHYM482-10|NZAC04032497|BOLD:AAR7114
Polistes humilis|NZHYM481-10|NZAC04032877|BOLD:AAR7114
Polistes chinensis|NZHYM479-10|NZAC04031499|BOLD:AAR7976
Polistes chinensis|NZIND015-22|NZAC03014454|BOLD:AAR7976
Polistes chinensis|NZHYM480-10|NZAC04032883|BOLD:AAR7976
Ancistrocerus gazella|NZIND277-22|NZAC03029722|BOLD:AAF4164
Ancistrocerus gazella|NZHYM478-10|NZAC04032435|BOLD:AAF4164
Ancistrocerus gazella|NZHYM477-10|NZAC04038019|BOLD:AAF4164
Bocchus thorpei|NZHYM501-10|NZAC04034004|BOLD:AAU4036
Bocchus thorpei|NZHYM504-10|NZAC04033363|BOLD:AAU4036
Bocchus thorpei|NZHYM502-10|NZAC04036276|BOLD:AAU4036
Bocchus thorpei|NZHYM505-10|NZAC04036596|BOLD:AAU4036
Bocchus thorpei|NZHYM503-10|NZAC04037635
Hypoponera confinis|NZHYM368-10|NZAC04032142|BOLD:AAX2134
Ponera leae|NZHYM200-10|NZAC04035034|BOLD:AAV6585
Ponera leae|NZHYM201-10|NZAC04036195|BOLD:AAV6585
Ponera leae|NZHYM198-10|NZAC04035818|BOLD:AAV6585
Ponera leae|NZHYM199-10|NZAC04036514|BOLD:AAV6585
Ponera leae|NZHYM202-10|NZAC04036639|BOLD:AAV6585
Austroponera|NZHYM210-10|NZAC04035977|BOLD:AAU1910
Austroponera|NZHYM209-10|NZAC04035476|BOLD:AAU1910
Austroponera|NZHYM220-10|NZAC04035068|BOLD:AAU1910
Austroponera|NZHYM212-10|NZAC04035055|BOLD:AAU1910
Austroponera|NZHYM213-10|NZAC04034690|BOLD:AAU1910
Austroponera|NZHYM211-10|NZAC04037088|BOLD:AAU1910
Austroponera|NZHYM354-10|NZAC04035623|BOLD:AAU1910
Austroponera|NZHYM351-10|NZAC04033173|BOLD:AAU1910
Austroponera|NZHYM353-10|NZAC04036196|BOLD:AAU1910
Austroponera|NZHYM352-10|NZAC04037282|BOLD:AAU1910
Austroponera|NZHYM223-10|NZAC04033705|BOLD:AAU1910
Austroponera|NZHYM355-10|NZAC04036323|BOLD:AAU1910
Austroponera|NZIND289-22|NZAC03029734|BOLD:AAU1910
Austroponera|NZHYM221-10|NZAC04034256|BOLD:AAU1910
Austroponera|NZHYM222-10|NZAC04035973|BOLD:AAU1910
Austroponera|NZHYM357-10|NZAC04033037|BOLD:AAU1910
Austroponera|NZHYM358-10|NZAC04036184|BOLD:AAU1910
Austroponera|NZHYM360-10|NZAC04033277|BOLD:AAU1910
Austroponera|NZHYM356-10|NZAC04034584|BOLD:AAU1910
Austroponera|NZHYM359-10|NZAC04037837|BOLD:AAU1910
Amblyopone australis|NZHYM275-10|NZAC04035551|BOLD:ABZ9654
Amblyopone australis|NZHYM194-10|NZAC04034191|BOLD:ADN5880
Amblyopone australis|NZHYM192-10|NZAC04036048|BOLD:AAU1994
Amblyopone australis|NZHYM274-10|NZAC04036249|BOLD:AAU1996
Amblyopone australis|NZHYM193-10|NZAC04037718|BOLD:AAU1995
Amblyopone saundersi|NZHYM195-10|NZAC04039455|BOLD:ABX8131
Amblyopone saundersi|NZHYM196-10|NZAC04039972|BOLD:AAV9963
Amblyopone saundersi|NZHYM2080-13|NZAC04087336|BOLD:ACL8166
Ochetellus glaber|NZHYM281-10|NZAC04033370|BOLD:AAK3157
Ochetellus glaber|NZHYM282-10|NZAC04035258|BOLD:AAK3157
Ochetellus glaber|NZHYM280-10|NZAC04036939|BOLD:AAK3157
Iridomyrmex suchieri|NZHYM272-10|NZAC04036999|BOLD:AAG3977
Iridomyrmex suchieri|NZHYM273-10|NZAC04035520|BOLD:AAG3977
Iridomyrmex suchieri|NZHYM001-10|MangereB|BOLD:AAG3977
Iridomyrmex suchieri|NZHYM269-10|NZAC04033449|BOLD:AAG3977
Iridomyrmex suchieri|NZHYM270-10|NZAC04034724|BOLD:AAG3977
Iridomyrmex suchieri|NZHYM271-10|NZAC04037405|BOLD:AAG3977
Linepithema humile|NZHYM288-10|NZAC04036794|BOLD:AAB2871
Linepithema humile|NZHYM287-10|NZAC04036875|BOLD:AAB2871
Doleromyrma darwiniana|NZHYM301-10|NZAC04034155|BOLD:ABX8407
Doleromyrma darwiniana|NZHYM1962-13|NZAC04049415|BOLD:ABX8407
Technomyrmex jocosus|NZHYM003-10|06PapakuraAK|BOLD:AAG9283
Technomyrmex jocosus|NZIND047-22|NZAC03014606|BOLD:AAG9283
Technomyrmex|NZHYM2154-13|NZAC04086535|BOLD:AAG9283
Strumigenys xenos|NZHYM242-10|NZAC04037570|BOLD:AAZ2652
Strumigenys perplexa|NZIND022-22|NZAC03014465|BOLD:ADU7876
Strumigenys perplexa|NZHYM285-10|NZAC04034997|BOLD:AAU2726
Strumigenys perplexa|NZHYM283-10|NZAC04037103|BOLD:AAU2726
Strumigenys perplexa|NZHYM286-10|NZAC04037610|BOLD:AAU2726
Strumigenys perplexa|NZHYM284-10|NZAC04037652|BOLD:AAU2726
Discothyrea antarctica|NZHYM345-10|NZAC04037141|BOLD:AAU2849
Discothyrea antarctica|NZHYM341-10|NZAC04037159|BOLD:AAU2849
Discothyrea antarctica|NZHYM343-10|NZAC04034395|BOLD:AAU2849
Discothyrea antarctica|NZHYM342-10|NZAC04034161|BOLD:AAU2849
Discothyrea antarctica|NZHYM344-10|NZAC04035045|BOLD:AAU2849
Discothyrea antarctica|NZHYM2081-13|NZAC04086105|BOLD:AAU2849
Tetramorium bicarinatum|NZHYM236-10|NZAC04037469|BOLD:AAA5578
Tetramorium grassii|NZHYM330-10|NZAC04034080|BOLD:AAP4318
Tetramorium grassii|NZHYM326-10|NZAC04036310|BOLD:AAP4318
Tetramorium grassii|NZHYM327-10|NZAC04037091|BOLD:AAP4318
Tetramorium grassii|NZHYM328-10|NZAC04037563|BOLD:AAP4318
Tetramorium grassii|NZHYM329-10|NZAC04037815|BOLD:AAP4318
Nylanderia|NZHYM325-10|NZAC04037155|BOLD:AAU2695
Nylanderia|NZHYM324-10|NZAC04035786|BOLD:AAU2694
Nylanderia|NZHYM311-10|NZAC04033273|BOLD:AAU2694
Nylanderia|NZHYM314-10|NZAC04036347|BOLD:AAU2694
Nylanderia|NZHYM313-10|NZAC04036103|BOLD:AAU2694
Nylanderia|NZHYM317-10|NZAC04035986|BOLD:AAU2694
Nylanderia|NZHYM320-10|NZAC04034962|BOLD:AAU2694
Nylanderia|NZHYM316-10|NZAC04036109|BOLD:AAU2694
Nylanderia|NZHYM321-10|NZAC04036763|BOLD:AAU2694
Nylanderia|NZHYM312-10|NZAC04037021|BOLD:AAU2694
Nylanderia|NZHYM315-10|NZAC04037324|BOLD:AAU2694

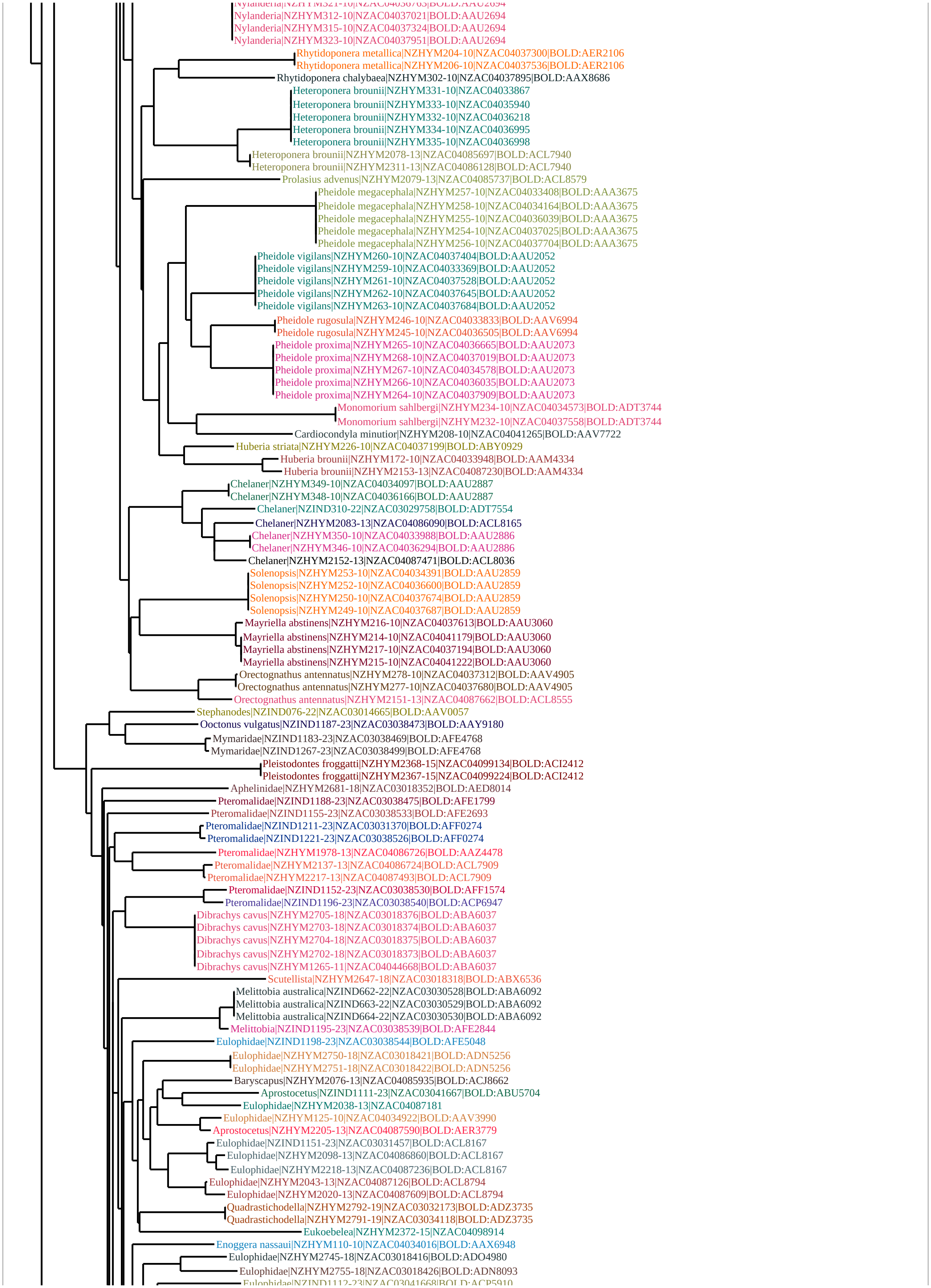
Nylanderia|NZHYM312-10|NZAC04037021|BOLD:AAU2694
Nylanderia|NZHYM315-10|NZAC04037324|BOLD:AAU2694
Nylanderia|NZHYM323-10|NZAC04037951|BOLD:AAU2694
Rhytidoponera metallica|NZHYM204-10|NZAC04037300|BOLD:AER2106
Rhytidoponera metallica|NZHYM206-10|NZAC04037536|BOLD:AER2106
Rhytidoponera chalybaea|NZHYM302-10|NZAC04037895|BOLD:AAX8686
Heteroponera brounii|NZHYM331-10|NZAC04033867
Heteroponera brounii|NZHYM333-10|NZAC04035940
Heteroponera brounii|NZHYM332-10|NZAC04036218
Heteroponera brounii|NZHYM334-10|NZAC04036995
Heteroponera brounii|NZHYM335-10|NZAC04036998
Heteroponera brounii|NZHYM2078-13|NZAC04085697|BOLD:ACL7940
Heteroponera brounii|NZHYM2311-13|NZAC04086128|BOLD:ACL7940
Prolasius advenus|NZHYM2079-13|NZAC04085737|BOLD:ACL8579
Pheidole megacephala|NZHYM257-10|NZAC04033408|BOLD:AAA3675
Pheidole megacephala|NZHYM258-10|NZAC04034164|BOLD:AAA3675
Pheidole megacephala|NZHYM255-10|NZAC04036039|BOLD:AAA3675
Pheidole megacephala|NZHYM254-10|NZAC04037025|BOLD:AAA3675
Pheidole megacephala|NZHYM256-10|NZAC04037704|BOLD:AAA3675
Pheidole vigilans|NZHYM260-10|NZAC04037404|BOLD:AAU2052
Pheidole vigilans|NZHYM259-10|NZAC04033369|BOLD:AAU2052
Pheidole vigilans|NZHYM261-10|NZAC04037528|BOLD:AAU2052
Pheidole vigilans|NZHYM262-10|NZAC04037645|BOLD:AAU2052
Pheidole vigilans|NZHYM263-10|NZAC04037684|BOLD:AAU2052
Pheidole rugosula|NZHYM246-10|NZAC04033833|BOLD:AAV6994
Pheidole rugosula|NZHYM245-10|NZAC04036505|BOLD:AAV6994
Pheidole proxima|NZHYM265-10|NZAC04036665|BOLD:AAU2073
Pheidole proxima|NZHYM268-10|NZAC04037019|BOLD:AAU2073
Pheidole proxima|NZHYM267-10|NZAC04034578|BOLD:AAU2073
Pheidole proxima|NZHYM266-10|NZAC04036035|BOLD:AAU2073
Pheidole proxima|NZHYM264-10|NZAC04037909|BOLD:AAU2073
Monomorium sahlbergi|NZHYM234-10|NZAC04034573|BOLD:ADT3744
Monomorium sahlbergi|NZHYM232-10|NZAC04037558|BOLD:ADT3744
Cardiocondyla minutior|NZHYM208-10|NZAC04041265|BOLD:AAV7722
Huberia striata|NZHYM226-10|NZAC04037199|BOLD:ABY0929
Huberia brounii|NZHYM172-10|NZAC04033948|BOLD:AAM4334
Huberia brounii|NZHYM2153-13|NZAC04087230|BOLD:AAM4334
Chelaner|NZHYM349-10|NZAC04034097|BOLD:AAU2887
Chelaner|NZHYM348-10|NZAC04036166|BOLD:AAU2887
Chelaner|NZIND310-22|NZAC03029758|BOLD:ADT7554
Chelaner|NZHYM2083-13|NZAC04086090|BOLD:ACL8165
Chelaner|NZHYM350-10|NZAC04033988|BOLD:AAU2886
Chelaner|NZHYM346-10|NZAC04036294|BOLD:AAU2886
Chelaner|NZHYM2152-13|NZAC04087471|BOLD:ACL8036
Solenopsis|NZHYM253-10|NZAC04034391|BOLD:AAU2859
Solenopsis|NZHYM252-10|NZAC04036600|BOLD:AAU2859
Solenopsis|NZHYM250-10|NZAC04037674|BOLD:AAU2859
Solenopsis|NZHYM249-10|NZAC04037687|BOLD:AAU2859
Mayriella abstinens|NZHYM216-10|NZAC04037613|BOLD:AAU3060
Mayriella abstinens|NZHYM214-10|NZAC04041179|BOLD:AAU3060
Mayriella abstinens|NZHYM217-10|NZAC04037194|BOLD:AAU3060
Mayriella abstinens|NZHYM215-10|NZAC04041222|BOLD:AAU3060
Orectognathus antennatus|NZHYM278-10|NZAC04037312|BOLD:AAV4905
Orectognathus antennatus|NZHYM277-10|NZAC04037680|BOLD:AAV4905
Orectognathus antennatus|NZHYM2151-13|NZAC04087662|BOLD:ACL8555
Stephanodes|NZIND076-22|NZAC03014665|BOLD:AAV0057
Ooctonus vulgatus|NZIND1187-23|NZAC03038473|BOLD:AAY9180
Mymaridae|NZIND1183-23|NZAC03038469|BOLD:AFE4768
Mymaridae|NZIND1267-23|NZAC03038499|BOLD:AFE4768
Pleistodontes froggatti|NZHYM2368-15|NZAC04099134|BOLD:ACI2412
Pleistodontes froggatti|NZHYM2367-15|NZAC04099224|BOLD:ACI2412
Aphelinidae|NZHYM2681-18|NZAC03018352|BOLD:AED8014
Pteromalidae|NZIND1188-23|NZAC03038475|BOLD:AFE1799
Pteromalidae|NZIND1155-23|NZAC03038533|BOLD:AFE2693
Pteromalidae|NZIND1211-23|NZAC03031370|BOLD:AFF0274
Pteromalidae|NZIND1221-23|NZAC03038526|BOLD:AFF0274
Pteromalidae|NZHYM1978-13|NZAC04086726|BOLD:AAZ4478
Pteromalidae|NZHYM2137-13|NZAC04086724|BOLD:ACL7909
Pteromalidae|NZHYM2217-13|NZAC04087493|BOLD:ACL7909
Pteromalidae|NZIND1152-23|NZAC03038530|BOLD:AFF1574
Pteromalidae|NZIND1196-23|NZAC03038540|BOLD:ACP6947
Dibrachys cavus|NZHYM2705-18|NZAC03018376|BOLD:ABA6037
Dibrachys cavus|NZHYM2703-18|NZAC03018374|BOLD:ABA6037
Dibrachys cavus|NZHYM2704-18|NZAC03018375|BOLD:ABA6037
Dibrachys cavus|NZHYM2702-18|NZAC03018373|BOLD:ABA6037
Dibrachys cavus|NZHYM1265-11|NZAC04044668|BOLD:ABA6037
Scutellista|NZHYM2647-18|NZAC03018318|BOLD:ABX6536
Melittobia australica|NZIND662-22|NZAC03030528|BOLD:ABA6092
Melittobia australica|NZIND663-22|NZAC03030529|BOLD:ABA6092
Melittobia australica|NZIND664-22|NZAC03030530|BOLD:ABA6092
Melittobia|NZIND1195-23|NZAC03038539|BOLD:AFE2844
Eulophidae|NZIND1198-23|NZAC03038544|BOLD:AFE5048
Eulophidae|NZHYM2750-18|NZAC03018421|BOLD:ADN5256
Eulophidae|NZHYM2751-18|NZAC03018422|BOLD:ADN5256
Baryscapus|NZHYM2076-13|NZAC04085935|BOLD:ACJ8662
Aprostocetus|NZIND1111-23|NZAC03041667|BOLD:ABU5704
Eulophidae|NZHYM2038-13|NZAC04087181
Eulophidae|NZHYM125-10|NZAC04034922|BOLD:AAV3990
Aprostocetus|NZHYM2205-13|NZAC04087590|BOLD:AER3779
Eulophidae|NZIND1151-23|NZAC03031457|BOLD:ACL8167
Eulophidae|NZHYM2098-13|NZAC04086860|BOLD:ACL8167
Eulophidae|NZHYM2218-13|NZAC04087236|BOLD:ACL8167
Eulophidae|NZHYM2043-13|NZAC04087126|BOLD:ACL8794
Eulophidae|NZHYM2020-13|NZAC04087609|BOLD:ACL8794
Quadrastichodella|NZHYM2792-19|NZAC03032173|BOLD:ADZ3735
Quadrastichodella|NZHYM2791-19|NZAC03034118|BOLD:ADZ3735
Eukoebelea|NZHYM2372-15|NZAC04098914
Enoggera nassaui|NZHYM110-10|NZAC04034016|BOLD:AAX6948
Eulophidae|NZHYM2745-18|NZAC03018416|BOLD:ADO4980
Eulophidae|NZHYM2755-18|NZAC03018426|BOLD:ADN8093

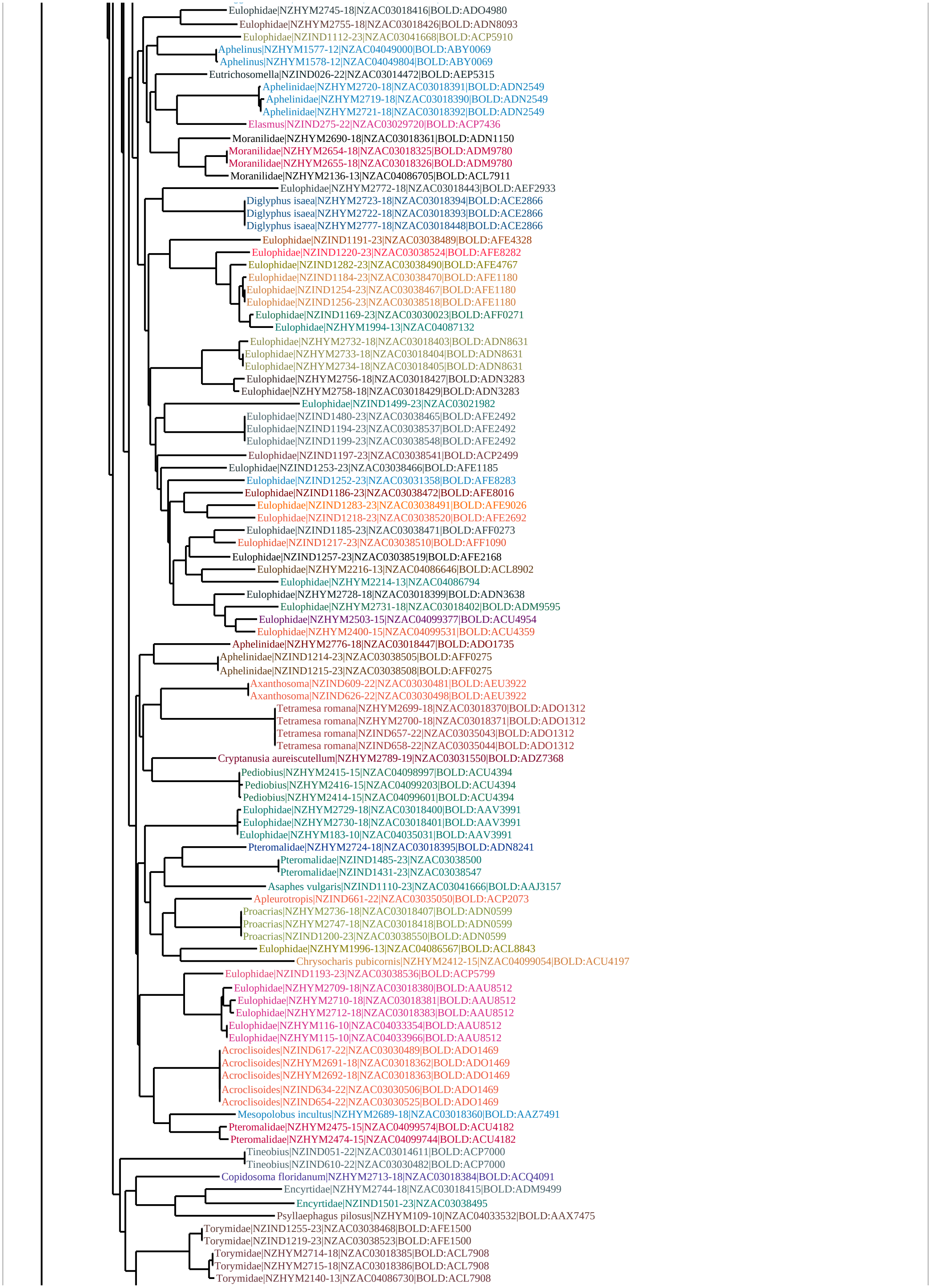
Eulophidae|NZHYM2745-18|NZAC03018416|BOLD:ADO4980
Eulophidae|NZHYM2755-18|NZAC03018426|BOLD:ADN8093
Eulophidae|NZIND1112-23|NZAC03041668|BOLD:ACP5910
Aphelinus|NZHYM1577-12|NZAC04049000|BOLD:ABY0069
Aphelinus|NZHYM1578-12|NZAC04049804|BOLD:ABY0069
Eutrichosomella|NZIND026-22|NZAC03014472|BOLD:AEP5315
Aphelinidae|NZHYM2720-18|NZAC03018391|BOLD:ADN2549
Aphelinidae|NZHYM2719-18|NZAC03018390|BOLD:ADN2549
Aphelinidae|NZHYM2721-18|NZAC03018392|BOLD:ADN2549
Elasmus|NZIND275-22|NZAC03029720|BOLD:ACP7436
Moranilidae|NZHYM2690-18|NZAC03018361|BOLD:ADN1150
Moranilidae|NZHYM2654-18|NZAC03018325|BOLD:ADM9780
Moranilidae|NZHYM2655-18|NZAC03018326|BOLD:ADM9780
Moranilidae|NZHYM2136-13|NZAC04086705|BOLD:ACL7911
Eulophidae|NZHYM2772-18|NZAC03018443|BOLD:AEF2933
Diglyphus isaea|NZHYM2723-18|NZAC03018394|BOLD:ACE2866
Diglyphus isaea|NZHYM2722-18|NZAC03018393|BOLD:ACE2866
Diglyphus isaea|NZHYM2777-18|NZAC03018448|BOLD:ACE2866
Eulophidae|NZIND1191-23|NZAC03038489|BOLD:AFE4328
Eulophidae|NZIND1220-23|NZAC03038524|BOLD:AFE8282
Eulophidae|NZIND1282-23|NZAC03038490|BOLD:AFE4767
Eulophidae|NZIND1184-23|NZAC03038470|BOLD:AFE1180
Eulophidae|NZIND1254-23|NZAC03038467|BOLD:AFE1180
Eulophidae|NZIND1256-23|NZAC03038518|BOLD:AFE1180
Eulophidae|NZIND1169-23|NZAC03030023|BOLD:AFF0271
Eulophidae|NZHYM1994-13|NZAC04087132
Eulophidae|NZHYM2732-18|NZAC03018403|BOLD:ADN8631
Eulophidae|NZHYM2733-18|NZAC03018404|BOLD:ADN8631
Eulophidae|NZHYM2734-18|NZAC03018405|BOLD:ADN8631
Eulophidae|NZHYM2756-18|NZAC03018427|BOLD:ADN3283
Eulophidae|NZHYM2758-18|NZAC03018429|BOLD:ADN3283
Eulophidae|NZIND1499-23|NZAC03021982
Eulophidae|NZIND1480-23|NZAC03038465|BOLD:AFE2492
Eulophidae|NZIND1194-23|NZAC03038537|BOLD:AFE2492
Eulophidae|NZIND1199-23|NZAC03038548|BOLD:AFE2492
Eulophidae|NZIND1197-23|NZAC03038541|BOLD:ACP2499
Eulophidae|NZIND1253-23|NZAC03038466|BOLD:AFE1185
Eulophidae|NZIND1252-23|NZAC03031358|BOLD:AFE8283
Eulophidae|NZIND1186-23|NZAC03038472|BOLD:AFE8016
Eulophidae|NZIND1283-23|NZAC03038491|BOLD:AFE9026
Eulophidae|NZIND1218-23|NZAC03038520|BOLD:AFE2692
Eulophidae|NZIND1185-23|NZAC03038471|BOLD:AFF0273
Eulophidae|NZIND1217-23|NZAC03038510|BOLD:AFF1090
Eulophidae|NZIND1257-23|NZAC03038519|BOLD:AFE2168
Eulophidae|NZHYM2216-13|NZAC04086646|BOLD:ACL8902
Eulophidae|NZHYM2214-13|NZAC04086794
Eulophidae|NZHYM2728-18|NZAC03018399|BOLD:ADN3638
Eulophidae|NZHYM2731-18|NZAC03018402|BOLD:ADM9595
Eulophidae|NZHYM2503-15|NZAC04099377|BOLD:ACU4954
Eulophidae|NZHYM2400-15|NZAC04099531|BOLD:ACU4359
Aphelinidae|NZHYM2776-18|NZAC03018447|BOLD:ADO1735
Aphelinidae|NZIND1214-23|NZAC03038505|BOLD:AFF0275
Aphelinidae|NZIND1215-23|NZAC03038508|BOLD:AFF0275
Axanthosoma|NZIND609-22|NZAC03030481|BOLD:AEU3922
Axanthosoma|NZIND626-22|NZAC03030498|BOLD:AEU3922
Tetramesa romana|NZHYM2699-18|NZAC03018370|BOLD:ADO1312
Tetramesa romana|NZHYM2700-18|NZAC03018371|BOLD:ADO1312
Tetramesa romana|NZIND657-22|NZAC03035043|BOLD:ADO1312
Tetramesa romana|NZIND658-22|NZAC03035044|BOLD:ADO1312
Cryptanusia aureiscutellum|NZHYM2789-19|NZAC03031550|BOLD:ADZ7368
Pediobius|NZHYM2415-15|NZAC04098997|BOLD:ACU4394
Pediobius|NZHYM2416-15|NZAC04099203|BOLD:ACU4394
Pediobius|NZHYM2414-15|NZAC04099601|BOLD:ACU4394
Eulophidae|NZHYM2729-18|NZAC03018400|BOLD:AAV3991
Eulophidae|NZHYM2730-18|NZAC03018401|BOLD:AAV3991
Eulophidae|NZHYM183-10|NZAC04035031|BOLD:AAV3991
Pteromalidae|NZHYM2724-18|NZAC03018395|BOLD:ADN8241
Pteromalidae|NZIND1485-23|NZAC03038500
Pteromalidae|NZIND1431-23|NZAC03038547
Asaphes vulgaris|NZIND1110-23|NZAC03041666|BOLD:AAJ3157
Apleurotropis|NZIND661-22|NZAC03035050|BOLD:ACP2073
Proacrias|NZHYM2736-18|NZAC03018407|BOLD:ADN0599
Proacrias|NZHYM2747-18|NZAC03018418|BOLD:ADN0599
Proacrias|NZIND1200-23|NZAC03038550|BOLD:ADN0599
Eulophidae|NZHYM1996-13|NZAC04086567|BOLD:ACL8843
Chrysocharis pubicornis|NZHYM2412-15|NZAC04099054|BOLD:ACU4197
Eulophidae|NZIND1193-23|NZAC03038536|BOLD:ACP5799
Eulophidae|NZHYM2709-18|NZAC03018380|BOLD:AAU8512
Eulophidae|NZHYM2710-18|NZAC03018381|BOLD:AAU8512
Eulophidae|NZHYM2712-18|NZAC03018383|BOLD:AAU8512
Eulophidae|NZHYM116-10|NZAC04033354|BOLD:AAU8512
Eulophidae|NZHYM115-10|NZAC04033966|BOLD:AAU8512
Acroclisoides|NZIND617-22|NZAC03030489|BOLD:ADO1469
Acroclisoides|NZHYM2691-18|NZAC03018362|BOLD:ADO1469
Acroclisoides|NZHYM2692-18|NZAC03018363|BOLD:ADO1469
Acroclisoides|NZIND634-22|NZAC03030506|BOLD:ADO1469
Acroclisoides|NZIND654-22|NZAC03030525|BOLD:ADO1469
Mesopolobus incultus|NZHYM2689-18|NZAC03018360|BOLD:AAZ7491
Pteromalidae|NZHYM2475-15|NZAC04099574|BOLD:ACU4182
Pteromalidae|NZHYM2474-15|NZAC04099744|BOLD:ACU4182
Tineobius|NZIND051-22|NZAC03014611|BOLD:ACP7000
Tineobius|NZIND610-22|NZAC03030482|BOLD:ACP7000
Copidosoma floridanum|NZHYM2713-18|NZAC03018384|BOLD:ACQ4091
Encyrtidae|NZHYM2744-18|NZAC03018415|BOLD:ADM9499
Encyrtidae|NZIND1501-23|NZAC03038495
Psyllaephagus pilosus|NZHYM109-10|NZAC04033532|BOLD:AAX7475
Torymidae|NZIND1255-23|NZAC03038468|BOLD:AFE1500
Torymidae|NZIND1219-23|NZAC03038523|BOLD:AFE1500
Torymidae|NZHYM2714-18|NZAC03018385|BOLD:ACL7908
Torymidae|NZHYM2715-18|NZAC03018386|BOLD:ACL7908
Torymidae|NZHYM2140-13|NZAC04086730|BOLD:ACL7908

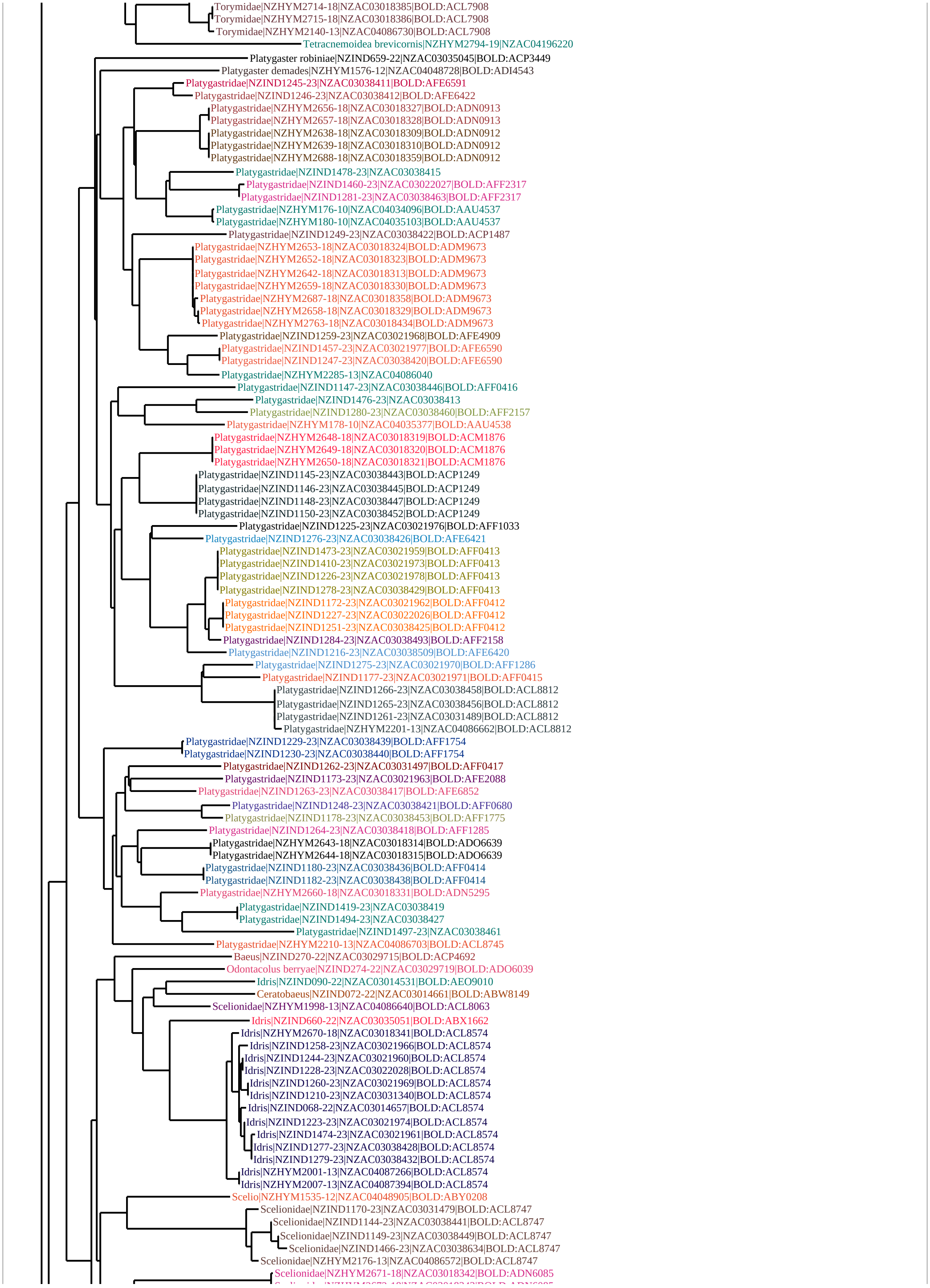
Torymidae|NZHYM2714-18|NZAC03018385|BOLD:ACL7908
Torymidae|NZHYM2715-18|NZAC03018386|BOLD:ACL7908
Torymidae|NZHYM2140-13|NZAC04086730|BOLD:ACL7908
Tetracnemoidea brevicornis|NZHYM2794-19|NZAC04196220
Platygaster robiniae|NZIND659-22|NZAC03035045|BOLD:ACP3449
Platygaster demades|NZHYM1576-12|NZAC04048728|BOLD:ADI4543
Platygastridae|NZIND1245-23|NZAC03038411|BOLD:AFE6591
Platygastridae|NZIND1246-23|NZAC03038412|BOLD:AFE6422
Platygastridae|NZHYM2656-18|NZAC03018327|BOLD:ADN0913
Platygastridae|NZHYM2657-18|NZAC03018328|BOLD:ADN0913
Platygastridae|NZHYM2638-18|NZAC03018309|BOLD:ADN0912
Platygastridae|NZHYM2639-18|NZAC03018310|BOLD:ADN0912
Platygastridae|NZHYM2688-18|NZAC03018359|BOLD:ADN0912
Platygastridae|NZIND1478-23|NZAC03038415
Platygastridae|NZIND1460-23|NZAC03022027|BOLD:AFF2317
Platygastridae|NZIND1281-23|NZAC03038463|BOLD:AFF2317
Platygastridae|NZHYM176-10|NZAC04034096|BOLD:AAU4537
Platygastridae|NZHYM180-10|NZAC04035103|BOLD:AAU4537
Platygastridae|NZIND1249-23|NZAC03038422|BOLD:ACP1487
Platygastridae|NZHYM2653-18|NZAC03018324|BOLD:ADM9673
Platygastridae|NZHYM2652-18|NZAC03018323|BOLD:ADM9673
Platygastridae|NZHYM2642-18|NZAC03018313|BOLD:ADM9673
Platygastridae|NZHYM2659-18|NZAC03018330|BOLD:ADM9673
Platygastridae|NZHYM2687-18|NZAC03018358|BOLD:ADM9673
Platygastridae|NZHYM2658-18|NZAC03018329|BOLD:ADM9673
Platygastridae|NZHYM2763-18|NZAC03018434|BOLD:ADM9673
Platygastridae|NZIND1259-23|NZAC03021968|BOLD:AFE4909
Platygastridae|NZIND1457-23|NZAC03021977|BOLD:AFE6590
Platygastridae|NZIND1247-23|NZAC03038420|BOLD:AFE6590
Platygastridae|NZHYM2285-13|NZAC04086040
Platygastridae|NZIND1147-23|NZAC03038446|BOLD:AFF0416
Platygastridae|NZIND1476-23|NZAC03038413
Platygastridae|NZIND1280-23|NZAC03038460|BOLD:AFF2157
Platygastridae|NZHYM178-10|NZAC04035377|BOLD:AAU4538
Platygastridae|NZHYM2648-18|NZAC03018319|BOLD:ACM1876
Platygastridae|NZHYM2649-18|NZAC03018320|BOLD:ACM1876
Platygastridae|NZHYM2650-18|NZAC03018321|BOLD:ACM1876
Platygastridae|NZIND1145-23|NZAC03038443|BOLD:ACP1249
Platygastridae|NZIND1146-23|NZAC03038445|BOLD:ACP1249
Platygastridae|NZIND1148-23|NZAC03038447|BOLD:ACP1249
Platygastridae|NZIND1150-23|NZAC03038452|BOLD:ACP1249
Platygastridae|NZIND1225-23|NZAC03021976|BOLD:AFF1033
Platygastridae|NZIND1276-23|NZAC03038426|BOLD:AFE6421
Platygastridae|NZIND1473-23|NZAC03021959|BOLD:AFF0413
Platygastridae|NZIND1410-23|NZAC03021973|BOLD:AFF0413
Platygastridae|NZIND1226-23|NZAC03021978|BOLD:AFF0413
Platygastridae|NZIND1278-23|NZAC03038429|BOLD:AFF0413
Platygastridae|NZIND1172-23|NZAC03021962|BOLD:AFF0412
Platygastridae|NZIND1227-23|NZAC03022026|BOLD:AFF0412
Platygastridae|NZIND1251-23|NZAC03038425|BOLD:AFF0412
Platygastridae|NZIND1284-23|NZAC03038493|BOLD:AFF2158
Platygastridae|NZIND1216-23|NZAC03038509|BOLD:AFE6420
Platygastridae|NZIND1275-23|NZAC03021970|BOLD:AFF1286
Platygastridae|NZIND1177-23|NZAC03021971|BOLD:AFF0415
Platygastridae|NZIND1266-23|NZAC03038458|BOLD:ACL8812
Platygastridae|NZIND1265-23|NZAC03038456|BOLD:ACL8812
Platygastridae|NZIND1261-23|NZAC03031489|BOLD:ACL8812
Platygastridae|NZHYM2201-13|NZAC04086662|BOLD:ACL8812
Platygastridae|NZIND1229-23|NZAC03038439|BOLD:AFF1754
Platygastridae|NZIND1230-23|NZAC03038440|BOLD:AFF1754
Platygastridae|NZIND1262-23|NZAC03031497|BOLD:AFF0417
Platygastridae|NZIND1173-23|NZAC03021963|BOLD:AFE2088
Platygastridae|NZIND1263-23|NZAC03038417|BOLD:AFE6852
Platygastridae|NZIND1248-23|NZAC03038421|BOLD:AFF0680
Platygastridae|NZIND1178-23|NZAC03038453|BOLD:AFF1775
Platygastridae|NZIND1264-23|NZAC03038418|BOLD:AFF1285
Platygastridae|NZHYM2643-18|NZAC03018314|BOLD:ADO6639
Platygastridae|NZHYM2644-18|NZAC03018315|BOLD:ADO6639
Platygastridae|NZIND1180-23|NZAC03038436|BOLD:AFF0414
Platygastridae|NZIND1182-23|NZAC03038438|BOLD:AFF0414
Platygastridae|NZHYM2660-18|NZAC03018331|BOLD:ADN5295
Platygastridae|NZIND1419-23|NZAC03038419
Platygastridae|NZIND1494-23|NZAC03038427
Platygastridae|NZIND1497-23|NZAC03038461
Platygastridae|NZHYM2210-13|NZAC04086703|BOLD:ACL8745
Baeus|NZIND270-22|NZAC03029715|BOLD:ACP4692
Odontacolus berryae|NZIND274-22|NZAC03029719|BOLD:ADO6039
Idris|NZIND090-22|NZAC03014531|BOLD:AEO9010
Ceratobaeus|NZIND072-22|NZAC03014661|BOLD:ABW8149
Scelionidae|NZHYM1998-13|NZAC04086640|BOLD:ACL8063
Idris|NZIND660-22|NZAC03035051|BOLD:ABX1662
Idris|NZHYM2670-18|NZAC03018341|BOLD:ACL8574
Idris|NZIND1258-23|NZAC03021966|BOLD:ACL8574
Idris|NZIND1244-23|NZAC03021960|BOLD:ACL8574
Idris|NZIND1228-23|NZAC03022028|BOLD:ACL8574
Idris|NZIND1260-23|NZAC03021969|BOLD:ACL8574
Idris|NZIND1210-23|NZAC03031340|BOLD:ACL8574
Idris|NZIND068-22|NZAC03014657|BOLD:ACL8574
Idris|NZIND1223-23|NZAC03021974|BOLD:ACL8574
Idris|NZIND1474-23|NZAC03021961|BOLD:ACL8574
Idris|NZIND1277-23|NZAC03038428|BOLD:ACL8574
Idris|NZIND1279-23|NZAC03038432|BOLD:ACL8574
Idris|NZHYM2001-13|NZAC04087266|BOLD:ACL8574
Idris|NZHYM2007-13|NZAC04087394|BOLD:ACL8574
Scelio|NZHYM1535-12|NZAC04048905|BOLD:ABY0208
Scelionidae|NZIND1170-23|NZAC03031479|BOLD:ACL8747
Scelionidae|NZIND1144-23|NZAC03038441|BOLD:ACL8747
Scelionidae|NZIND1149-23|NZAC03038449|BOLD:ACL8747
Scelionidae|NZIND1466-23|NZAC03038634|BOLD:ACL8747
Scelionidae|NZHYM2176-13|NZAC04086572|BOLD:ACL8747
Scelionidae|NZHYM2671-18|NZAC03018342|BOLD:ADN6085

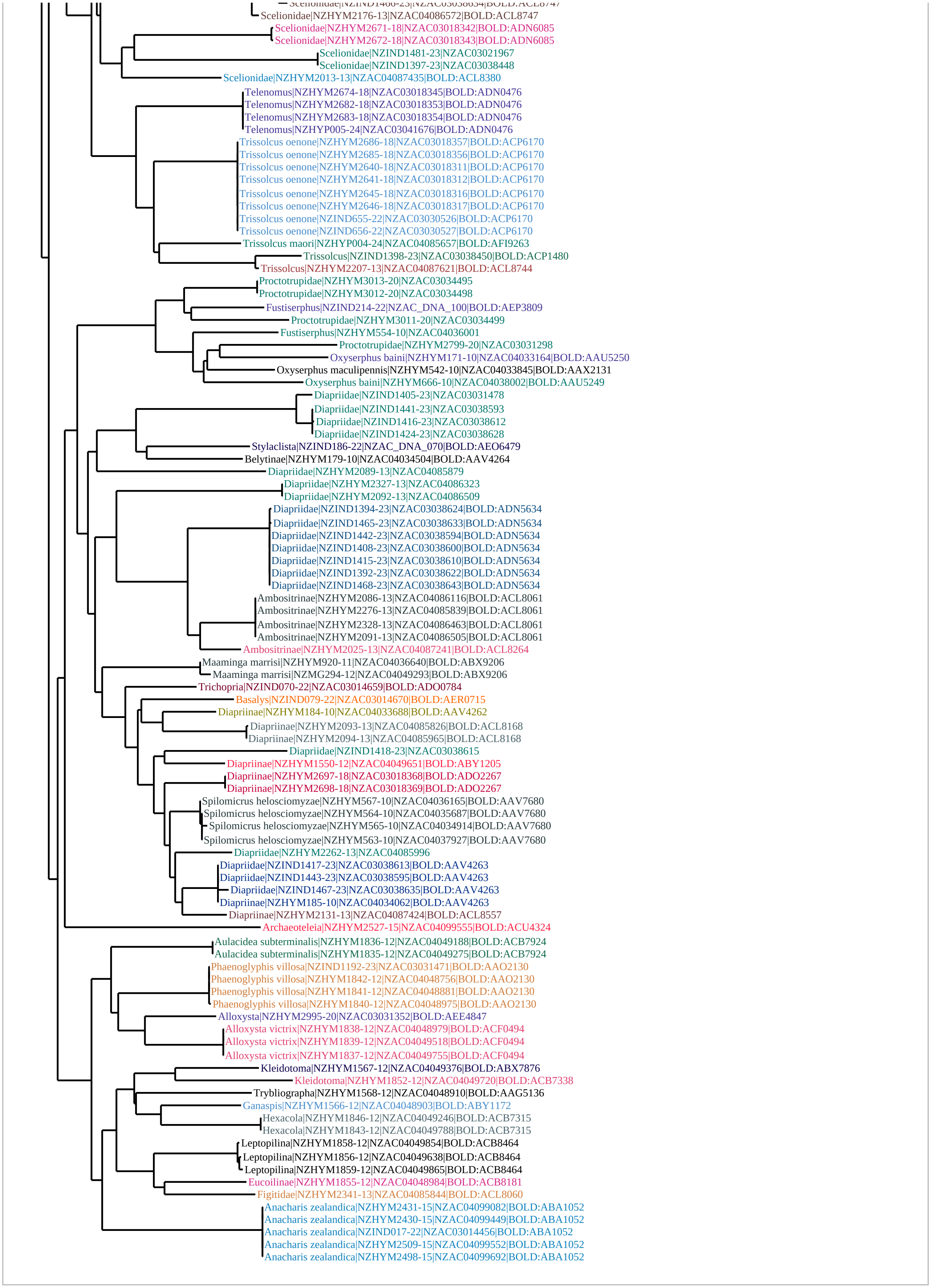
Scelionidae|NZIND1466-23|NZAC03038634|BOLD:ACL8747
Scelionidae|NZHYM2176-13|NZAC04086572|BOLD:ACL8747
Scelionidae|NZHYM2671-18|NZAC03018342|BOLD:ADN6085
Scelionidae|NZHYM2672-18|NZAC03018343|BOLD:ADN6085
Scelionidae|NZIND1481-23|NZAC03021967
Scelionidae|NZIND1397-23|NZAC03038448
Scelionidae|NZHYM2013-13|NZAC04087435|BOLD:ACL8380
Telenomus|NZHYM2674-18|NZAC03018345|BOLD:ADN0476
Telenomus|NZHYM2682-18|NZAC03018353|BOLD:ADN0476
Telenomus|NZHYM2683-18|NZAC03018354|BOLD:ADN0476
Telenomus|NZHYP005-24|NZAC03041676|BOLD:ADN0476
Trissolcus oenone|NZHYM2686-18|NZAC03018357|BOLD:ACP6170
Trissolcus oenone|NZHYM2685-18|NZAC03018356|BOLD:ACP6170
Trissolcus oenone|NZHYM2640-18|NZAC03018311|BOLD:ACP6170
Trissolcus oenone|NZHYM2641-18|NZAC03018312|BOLD:ACP6170
Trissolcus oenone|NZHYM2645-18|NZAC03018316|BOLD:ACP6170
Trissolcus oenone|NZHYM2646-18|NZAC03018317|BOLD:ACP6170
Trissolcus oenone|NZIND655-22|NZAC03030526|BOLD:ACP6170
Trissolcus oenone|NZIND656-22|NZAC03030527|BOLD:ACP6170
Trissolcus maori|NZHYP004-24|NZAC04085657|BOLD:AFI9263
Trissolcus|NZIND1398-23|NZAC03038450|BOLD:ACP1480
Trissolcus|NZHYM2207-13|NZAC04087621|BOLD:ACL8744
Proctotrupidae|NZHYM3013-20|NZAC03034495
Proctotrupidae|NZHYM3012-20|NZAC03034498
Fustiserphus|NZIND214-22|NZAC_DNA_100|BOLD:AEP3809
Proctotrupidae|NZHYM3011-20|NZAC03034499
Fustiserphus|NZHYM554-10|NZAC04036001
Proctotrupidae|NZHYM2799-20|NZAC03031298
Oxyserphus baini|NZHYM171-10|NZAC04033164|BOLD:AAU5250
Oxyserphus maculipennis|NZHYM542-10|NZAC04033845|BOLD:AAX2131
Oxyserphus baini|NZHYM666-10|NZAC04038002|BOLD:AAU5249
Diapriidae|NZIND1405-23|NZAC03031478
Diapriidae|NZIND1441-23|NZAC03038593
Diapriidae|NZIND1416-23|NZAC03038612
Diapriidae|NZIND1424-23|NZAC03038628
Stylaclista|NZIND186-22|NZAC_DNA_070|BOLD:AEO6479
Belytinae|NZHYM179-10|NZAC04034504|BOLD:AAV4264
Diapriidae|NZHYM2089-13|NZAC04085879
Diapriidae|NZHYM2327-13|NZAC04086323
Diapriidae|NZHYM2092-13|NZAC04086509
Diapriidae|NZIND1394-23|NZAC03038624|BOLD:ADN5634
Diapriidae|NZIND1465-23|NZAC03038633|BOLD:ADN5634
Diapriidae|NZIND1442-23|NZAC03038594|BOLD:ADN5634
Diapriidae|NZIND1408-23|NZAC03038600|BOLD:ADN5634
Diapriidae|NZIND1415-23|NZAC03038610|BOLD:ADN5634
Diapriidae|NZIND1392-23|NZAC03038622|BOLD:ADN5634
Diapriidae|NZIND1468-23|NZAC03038643|BOLD:ADN5634
Ambositrinae|NZHYM2086-13|NZAC04086116|BOLD:ACL8061
Ambositrinae|NZHYM2276-13|NZAC04085839|BOLD:ACL8061
Ambositrinae|NZHYM2328-13|NZAC04086463|BOLD:ACL8061
Ambositrinae|NZHYM2091-13|NZAC04086505|BOLD:ACL8061
Ambositrinae|NZHYM2025-13|NZAC04087241|BOLD:ACL8264
Maaminga marrisi|NZHYM920-11|NZAC04036640|BOLD:ABX9206
Maaminga marrisi|NZMG294-12|NZAC04049293|BOLD:ABX9206
Trichopria|NZIND070-22|NZAC03014659|BOLD:ADO0784
Basalys|NZIND079-22|NZAC03014670|BOLD:AER0715
Diapriinae|NZHYM184-10|NZAC04033688|BOLD:AAV4262
Diapriinae|NZHYM2093-13|NZAC04085826|BOLD:ACL8168
Diapriinae|NZHYM2094-13|NZAC04085965|BOLD:ACL8168
Diapriidae|NZIND1418-23|NZAC03038615
Diapriinae|NZHYM1550-12|NZAC04049651|BOLD:ABY1205
Diapriinae|NZHYM2697-18|NZAC03018368|BOLD:ADO2267
Diapriinae|NZHYM2698-18|NZAC03018369|BOLD:ADO2267
Spilomicrus helosciomyzae|NZHYM567-10|NZAC04036165|BOLD:AAV7680
Spilomicrus helosciomyzae|NZHYM564-10|NZAC04035687|BOLD:AAV7680
Spilomicrus helosciomyzae|NZHYM565-10|NZAC04034914|BOLD:AAV7680
Spilomicrus helosciomyzae|NZHYM563-10|NZAC04037927|BOLD:AAV7680
Diapriidae|NZHYM2262-13|NZAC04085996
Diapriidae|NZIND1417-23|NZAC03038613|BOLD:AAV4263
Diapriidae|NZIND1443-23|NZAC03038595|BOLD:AAV4263
Diapriidae|NZIND1467-23|NZAC03038635|BOLD:AAV4263
Diapriinae|NZHYM185-10|NZAC04034062|BOLD:AAV4263
Diapriinae|NZHYM2131-13|NZAC04087424|BOLD:ACL8557
Archaeoteleia|NZHYM2527-15|NZAC04099555|BOLD:ACU4324
Aulacidea subterminalis|NZHYM1836-12|NZAC04049188|BOLD:ACB7924
Aulacidea subterminalis|NZHYM1835-12|NZAC04049275|BOLD:ACB7924
Phaenoglyphis villosa|NZIND1192-23|NZAC03031471|BOLD:AAO2130
Phaenoglyphis villosa|NZHYM1842-12|NZAC04048756|BOLD:AAO2130
Phaenoglyphis villosa|NZHYM1841-12|NZAC04048881|BOLD:AAO2130
Phaenoglyphis villosa|NZHYM1840-12|NZAC04048975|BOLD:AAO2130
Alloxysta|NZHYM2995-20|NZAC03031352|BOLD:AEE4847
Alloxysta victrix|NZHYM1838-12|NZAC04048979|BOLD:ACF0494
Alloxysta victrix|NZHYM1839-12|NZAC04049518|BOLD:ACF0494
Alloxysta victrix|NZHYM1837-12|NZAC04049755|BOLD:ACF0494
Kleidotoma|NZHYM1567-12|NZAC04049376|BOLD:ABX7876
Kleidotoma|NZHYM1852-12|NZAC04049720|BOLD:ACB7338
Trybliographa|NZHYM1568-12|NZAC04048910|BOLD:AAG5136
Ganaspis|NZHYM1566-12|NZAC04048903|BOLD:ABY1172
Hexacola|NZHYM1846-12|NZAC04049246|BOLD:ACB7315
Hexacola|NZHYM1843-12|NZAC04049788|BOLD:ACB7315
Leptopilina|NZHYM1858-12|NZAC04049854|BOLD:ACB8464
Leptopilina|NZHYM1856-12|NZAC04049638|BOLD:ACB8464
Leptopilina|NZHYM1859-12|NZAC04049865|BOLD:ACB8464
Eucoilinae|NZHYM1855-12|NZAC04048984|BOLD:ACB8181
Figitidae|NZHYM2341-13|NZAC04085844|BOLD:ACL8060
Anacharis zealandica|NZHYM2431-15|NZAC04099082|BOLD:ABA1052
Anacharis zealandica|NZHYM2430-15|NZAC04099449|BOLD:ABA1052
Anacharis zealandica|NZIND017-22|NZAC03014456|BOLD:ABA1052
Anacharis zealandica|NZHYM2509-15|NZAC04099552|BOLD:ABA1052
Anacharis zealandica|NZHYM2498-15|NZAC04099692|BOLD:ABA1052
